# Evaluation of Dihydropyridine Derivatives as Aldose Reductase Inhibitors: Enzymatic Screening, Molecular Docking and Ex Vivo Assessment of a Selected Compound in a Lens Model

**DOI:** 10.3390/ijms27219642

**Published:** 2026-10-28

**Authors:** Katia Lizbeth Alonso-Hurtado, Tania Méndez-Pérez, Zurisaddai Hernández-Gallegos, Marcos Cajero-Juárez, Pedro Navarro-Santos, José J. Virgen-Ortíz, Mauro M. Martínez-Pacheco

**Affiliations:** 1 Instituto de Investigaciones Químico Biológicas, Universidad Michoacana de San Nicolás de Hidalgo, Morelia 58040, Mexico; 2 Instituto de Investigaciones Agrícolas y Forestales, Universidad Michoacana de San Nicolás de Hidalgo, Morelia 58040, Mexico; 3 Centro de Investigación en Alimentación y Desarrollo A.C., Morelia 58341, Mexico; jose.virgen@ciad.mx

**Keywords:** aldose reductase, 1,4-dihydropyridines, molecular docking, enzyme kinetics, diabetic cataracts

## Abstract

Aldose reductase (AR) is a key enzyme in the polyol pathway and has been widely implicated in the development of diabetic complications, including cataract formation. In this work, a series of structurally modified 1,4-dihydropyridine (DHP) derivatives were evaluated as potential AR inhibitors. The compounds were obtained with high purity and subjected to enzymatic screening, exhibiting IC_50_ values ranging from 1.11 ± 0.07 to 55.34 ± 0.06 µM. The unsubstituted DHP scaffold exhibited intrinsic activity, whereas substitution at the 4-phenyl ring modulated inhibition in a nonlinear manner, reflecting the combined influence of steric and electronic factors on enzyme interactions. Kinetic analysis revealed mixed-type inhibition, indicating that the inhibitory behavior of the DHP derivatives cannot be explained solely by simple competition with the substrate. Molecular docking using the AR–NADP complex identified favorable binding poses and interactions with residues involved in ligand recognition and stabilization, although no direct correlation was observed between predicted docking scores and experimental inhibitory potency. A representative compound (DHP3, IC_50_ = 1.11 ± 0.07 µM) was elected for ex vivo evaluation in a rabbit lens model under hyperglycemic conditions. Treatment reduced glucose-induced lens opacity by 89.1%, estimated by image-based analysis, and decreased sorbitol accumulation by 57.48% relative to hyperglycemic control, indicating modulation of the polyol pathway at the tissue level. In contrast, no significant changes were detected in calcium content. These findings support the potential of DHP derivatives as a scaffold for aldose reductase inhibition and highlight their ability to attenuate early alterations associated with hyperglycemia in lens tissue. Further studies are required to expand biological validation and optimize their inhibitory profile.

## 1. Introduction

Diabetes mellitus (DM) is a global health concern with a rapidly increasing prevalence, and its chronic complications significantly impair quality of life [1]. Cataract remains one of the leading causes of blindness worldwide, particularly among individuals with prolonged hyperglycemia [2]. The development of diabetic cataract is strongly associated with metabolic alterations in the lens, where sustained glucose levels activate biochemical pathways that compromise lens transparency [3]. A key mechanism involved in cataract formation under hyperglycemic conditions is the activation of the polyol pathway [4]. In this pathway, aldose reductase (AR; AKR1B1, EC 1.1.1.21) catalyzes the NADPH-dependent reduction in glucose to sorbitol [5]. Due to the limited activity of sorbitol dehydrogenase in the lens, sorbitol accumulates intracellularly, leading to osmotic stress, cellular swelling, and structural damage [6]. In addition, NADPH consumption reduces the availability of this cofactor for antioxidant defense systems, thereby exacerbating oxidative stress and promoting protein aggregation within the lens [7]. Recent studies indicate that dysregulation of AR and the polyol pathway contributes to oxidative stress and cellular dysfunction in diabetic complications [8,9]. Others comprehensive reviews to identify AKR1B1 as one of the most promising therapeutic targets for diabetes-associated complications, while emphasizing the need for novel inhibitors with improved efficacy, selectivity, and pharmacokinetic properties [10]. However, despite extensive research, many AR inhibitors have shown limited success in biological systems, mainly due to poor bioavailability, ionization at physiological pH and off-target effects that may lead to adverse pharmacological outcomes [11,12]. Recent analyses of the aldose reductase inhibitor field indicate that these limitations remain the major obstacle to successful clinical translation, highlighting the need for alternative chemical scaffolds [13]. These limitations have driven the search for alternative scaffolds capable of modulating AR activity. Heterocyclic compounds, particularly 1,4-dihydropyridine (DHPs), have attracted considerable attention due to their broad spectrum of biological activities and their structural versatility, which allows modulation of physicochemical and pharmacological properties through substitution at the aromatic ring [14]. Although widely recognized as voltage-gated calcium channel blockers, their pharmacological properties are strongly influenced by their chemical structure, especially substitution at the 4-phenyl ring, which can modulate interactions with biological targets [15]. Recent evidence indicates that several 1,4-dihydropyridine calcium channel blockers may also inhibit aldose reductase. Türkeş et al. reported that nifedipine, nitrendipine, isradipine, nilvadipine, and amlodipine inhibited aldose reductase isolated from sheep kidney, supporting the interaction of these calcium channel blockers with aldose reductase [16]. More recently, Devi et al. demonstrated that nifedipine inhibits recombinant human aldose reductase with an IC_50_ of 2.5 µM and delays cataract progression in streptozotocin-induced diabetic rats [17]. Nevertheless, these studies have primarily focused on individual clinically used calcium channel blockers rather than on structurally related 4-phenyl-1,4-dihydropyridine specifically designed to evaluate the influence of aromatic substitution on aldose reductase inhibition. Furthermore, comprehensive studies integrating enzymatic inhibition, inhibition kinetics, molecular docking, and biological validation in diabetic cataract models remain scarce [3]. Therefore, the present study investigated a structurally related series of Hantzsch-type 4-phenyl-1,4-dihydropyridines as potential inhibitors of recombinant human aldose reductase by integrating enzymatic inhibition, inhibition kinetics, molecular docking, and ex vivo validation in rabbit lens model of diabetic cataract. Structural variations at the 4-phenyl ring included the introduction of halogen and electron-modulating substituents to explore their effect on inhibitory activity. The compounds were assessed through enzymatic inhibition assays, kinetic analysis and molecular docking to characterize enzyme-ligand interactions. Additionally, a representative compound was selected for preliminary evaluation in an ex vivo rabbit lens model under hyperglycemic conditions. Lens opacity and sorbitol accumulation were used as indicators of alterations associated with polyol pathway activation, while calcium levels were determined as a complementary parameter of lens homeostasis. This approach provides insight into the inhibitory potential of 1,4-dihydropyridine derivatives against aldose reductase and their ability to modulate early events associated with diabetic cataract formation.

## 2. Results

### 2.1. Synthesis and Structural Characterization of DHP Derivatives

A series of 1,4-dihydropyridine derivatives (DHP1–DHP9) was synthesized through a modified Hantzsch reaction. A series of previously reported 1,4-dihydropyridine derivatives (DHP1–DHP9) was synthesized using established Hantzsch-type procedures described by Hernández-Gallegos et al. [18] and Noyola and Hernández-Gallegos [19]. The series comprised derivatives bearing different substituents at the 4-phenyl ring, including halogenated derivatives as well as compounds bearing electron-donating and strongly electron-withdrawing substituents.

The chemical structures and substitution patterns of the synthesized compounds are shown in Figure 1. The introduced substitutions did not interfere with the formation of the dihydropyridine scaffold, allowing successful synthesis of all target derivatives. The structural identity of the synthesized derivatives was assessed by ^1^H and ^13^C nuclear magnetic resonance (NMR) spectroscopy. The spectroscopic data were consistent with the expected 1,4-dihydropyridine structures and their corresponding substitution patterns. Complete ^1^H and ^13^C NMR data and spectra for DHP1-DHP9 are provided in the Supplementary Materials (Figures S1–S18).

The chemical structures, substituent groups, and purity of the nine synthesized 1,4-dihydropyridine derivatives are summarized in Table 1. All compounds share the characteristics of the 1,4-dihydropyridine core, bearing a phenyl ring with substituents in the meta and para positions. The purity of the compounds ranged from 94.23% to 99.29% as determined by HPLC analysis (Figures S19–S27), indicating that all derivatives were suitable for subsequent biological evaluation. The selected substitution patterns were designed to modulate the electronic and steric properties of the phenyl ring, which may influence interactions with biological targets. These structural features were considered relevant for assessing the inhibitory potential of the synthesized compounds against aldose reductase.

### 2.2. In Vitro Aldose Reductase Inhibition

The inhibitory activity of the synthesized DHP derivatives against aldose reductase was evaluated in vitro. The compounds exhibited different inhibition profiles depending on the substituents present at the 4-phenyl ring. The obtained IC_50_ values are summarized in Table 2. Several halogen-substituted derivatives exhibited lower IC_50_ values compared with the unsubstituted scaffold, although no clear linear relationship between substitution pattern and inhibitory potency was observed. Among the evaluated derivatives, DHP3 exhibited the lowest IC_50_ value under the experimental conditions employed.

### 2.3. Kinetic Analysis of AR Inhibition

**Figure 2:**
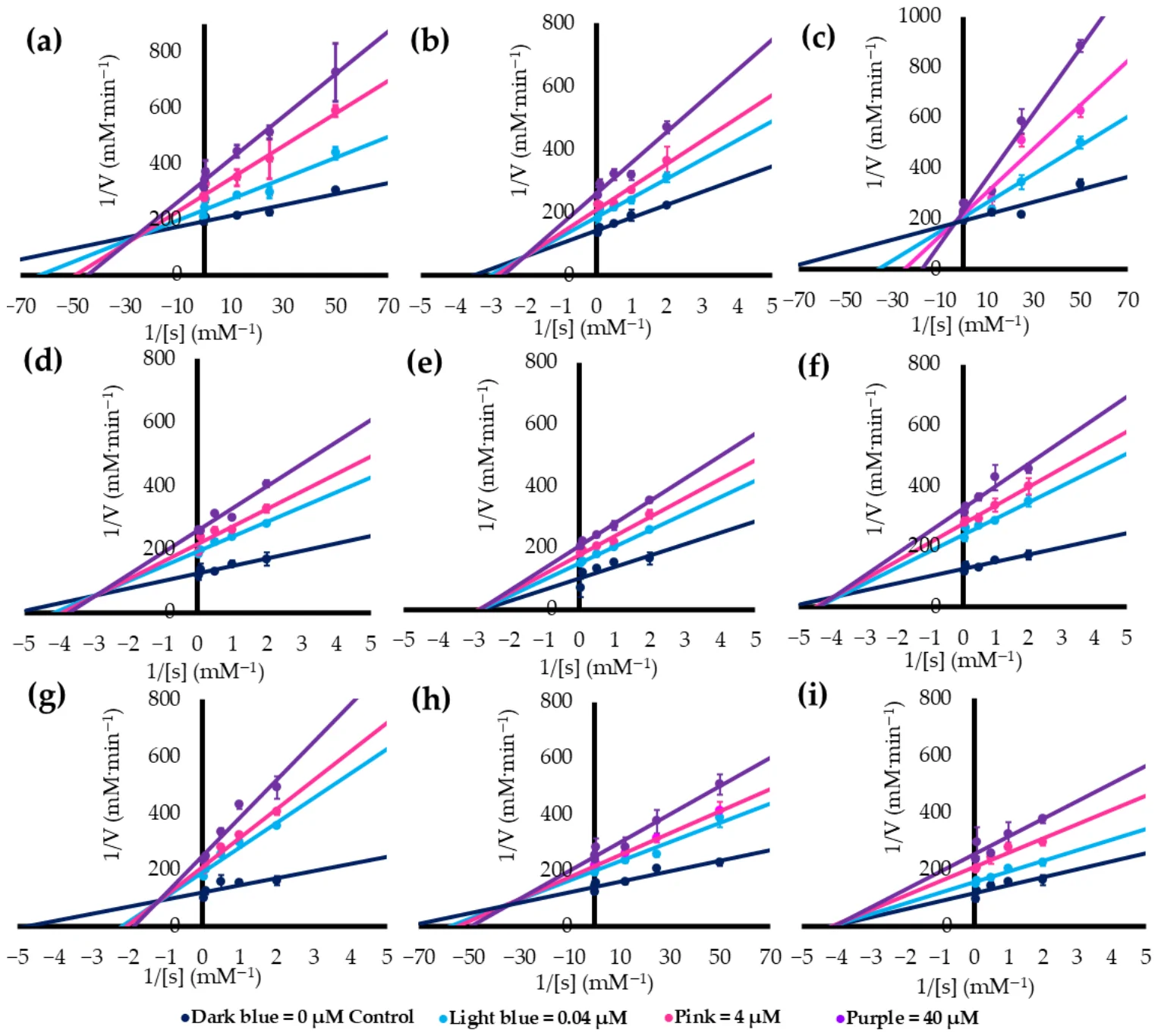
In vitro inhibition of aldose reductase (AR) by DHP1–DHP9. Lineweaver–Burk plots were used to determine the inhibition type and estimate kinetic parameters. Panels (**a**–**i**) correspond to DHP1–DHP9, respectively. For DHP1, DHP3, and DHP8, enzyme activity was evaluated using final DL-glyceraldehyde concentrations of 0.02, 0.04, 0.08, 2.0, 4.0, and 20.0 mM. For the remaining derivatives, final DL-glyceraldehyde concentrations of 0.5, 1.0, 2.0, 10.0, and 20.0 mM were used. NADPH was maintained at a constant final concentration of 0.20 mM in all assays. Colors indicate inhibitor concentrations as follows: dark blue, 0 µM (control); light blue, 0.04 µM; pink, 4 µM; and purple, 40 µM. Data points are presented as mean ± SEM from three independent experiments (*n* = 3). Error bars represent the standard error of the mean (SEM); when smaller than the corresponding symbols, they may not be visible. Lines represent linear regression fits of the reciprocal kinetic data.

The inhibition mechanisms of DHP1–DHP9 against aldose reductase were evaluated by Lineweaver–Burk kinetic analysis using different DL-glyceraldehyde concentrations. hAR basal enzyme kinetic parameters were ^app^Km_gly_ = 0.37 mM and ^app^Vmax_gly_ = 0.01 mM·min^−1^. The corresponding double-reciprocal plots are presented in Figure 2, while the calculated kinetic parameters are summarized in Table 2. Differences in inhibition behaviour were observed among the synthesized derivatives depending on the substitution pattern at the 4-phenyl ring. Kinetic profiles consistent with mixed and non-competitive inhibition were observed for the evaluated compounds. Variations in Ki and Ki′ values suggested differences in affinity toward the free enzyme and the enzyme–substrate complex.

### 2.4. Molecular Docking Studies

**Figure 3:**
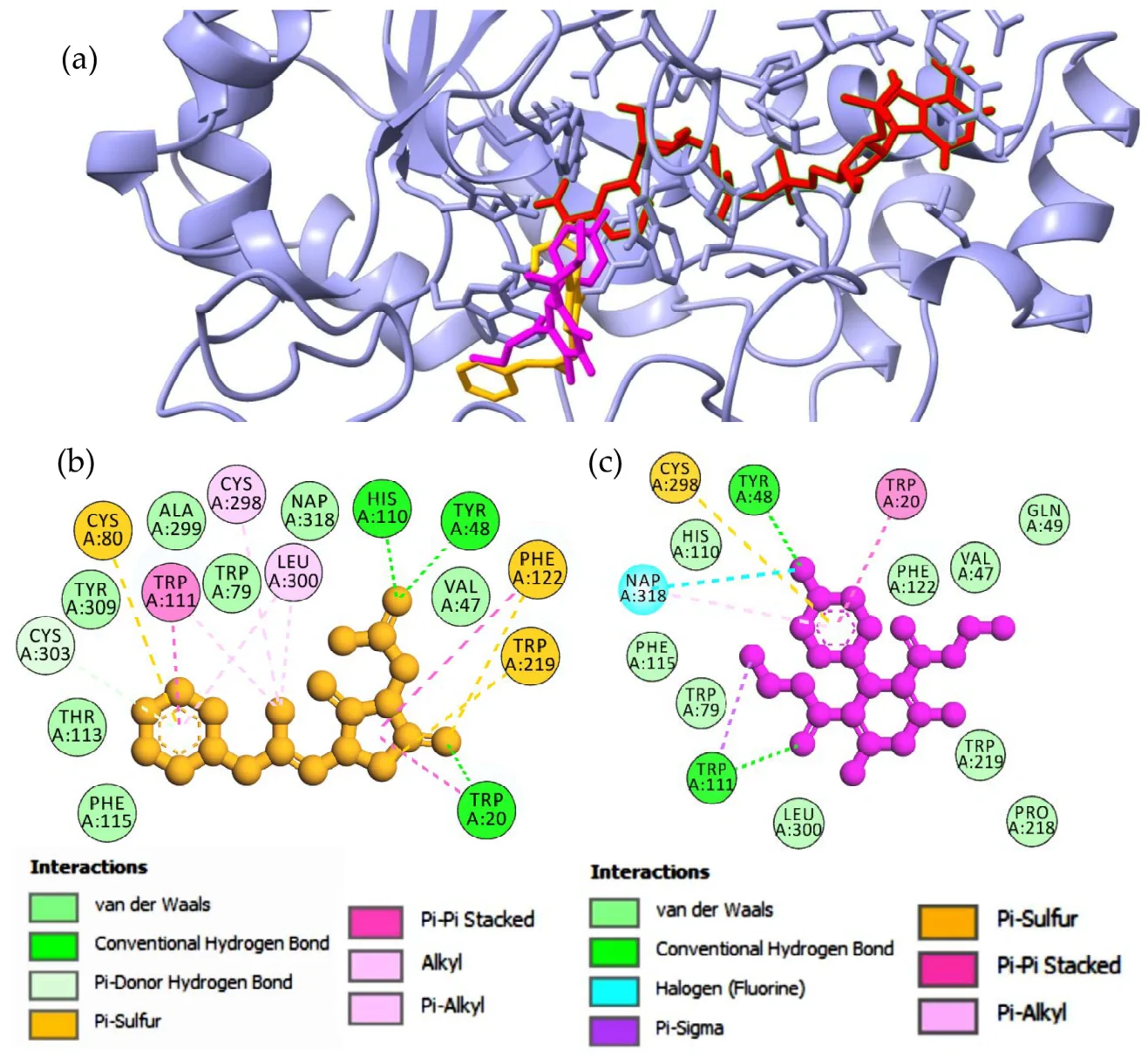
Molecular docking analysis of selected ligands with human aldose reductase (AR). Docking calculations were performed using the AR–NADP binary complex derived from PDB ID 1PWL. (**a**) Comparative representation of the predicted binding poses of epalrestat and DHP3 within the AR active site. The protein is shown as a light-purple ribbon, the NADP cofactor in red, epalrestat in yellow, and DHP3 in magenta. (**b**) Two-dimensional interaction diagram of the best-ranked docking pose of epalrestat, showing the principal predicted interactions with surrounding amino acid residues and the NADP cofactor. (**c**) Two-dimensional interaction diagram of the best-ranked docking pose of DHP3, showing the principal predicted interactions with surrounding amino acid residues and the NADP cofactor. Interaction types are indicated according to the color legends shown in each panel. Abbreviations: AR, aldose reductase; DHP3, 1,4-dihydropyridine derivative 3; NADP, nicotinamide adenine dinucleotide phosphate.

The comparative binding poses of DHP3 and epalrestat within the AR active site are shown in Figure 3, whereas the individual two-dimensional interaction profiles of DHP1–DHP9 are presented in Figure S28. Differences in the interaction patterns were observed among the DHP derivatives, indicating that substitution at the 4-phenyl ring influenced the predicted contacts established within the binding pocket. Importantly, the docking scores did not correlate directly with the experimentally determined inhibitory potency. DHP3, which exhibited the strongest experimental inhibition, did not show the most favorable predicted binding energy, suggesting that differences in inhibitory activity cannot be explained solely by docking scores and may also depend on the specific interaction geometry and other structural or dynamic factors not fully captured by the docking model.

The docking protocol was validated by redocking co-crystallized inhibitor minalrestat into the unoccupied active site of the AR–NADP complex. The best-ranked redocked pose closely reproduced the crystallographic binding orientation, with an RMSD of 0.41 Å (Figure S29), supporting the reliability of the revised docking protocol. The predicted binding affinities and key residue interactions of the evaluated DHP derivatives with AR are summarized in Table 3.

### 2.5. Ex Vivo Evaluation of Lens Transparency Under Hyperglycemic Conditions

Representative images of isolated rabbit lenses cultured under hyperglycemic conditions revealed a marked loss of transparency compared with the normoglycemic control group. Lenses incubated for 6 days with 50 mM glucose showed evident opacification that impaired visualization of the underlying mesh grid. A similar opaque appearance was observed in the DMSO-treated group. In contrast, lenses treated with epalrestat (4 µM) or DHP3 (4 µM) retained a greater degree of transparency under hyperglycemic conditions. Among the evaluated treatments, the DHP3-treated lenses exhibited greater preservation of transparency relative to the untreated hyperglycemic group. These observations were further supported by quantitative image analysis.

### 2.6. Image Processing for Lens Opacity Analysis

**Figure 4:**
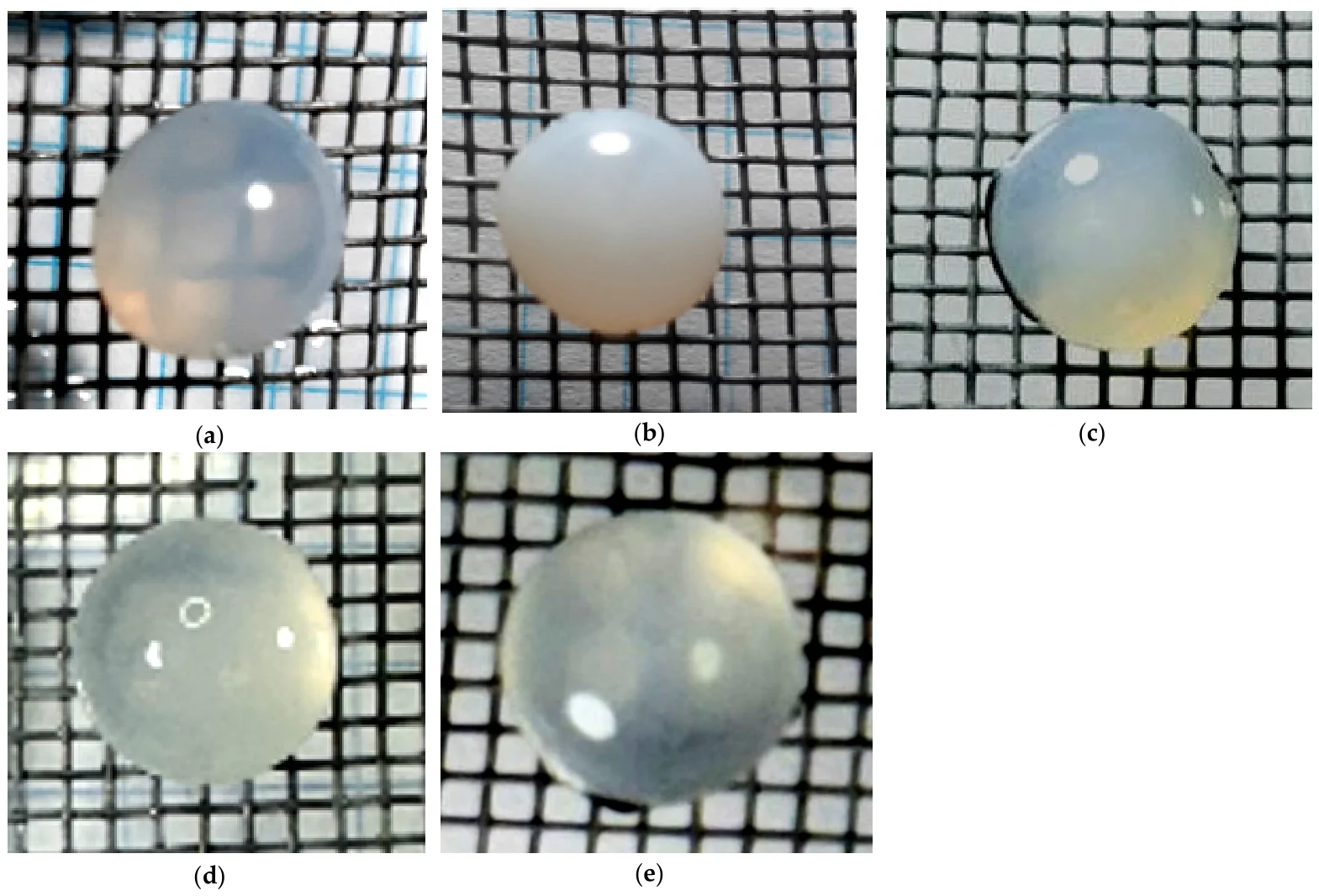
Representative images of lenses after 6 days of culture under different experimental conditions in an ex vivo model. (**a**) 5 mM glucose (control); (**b**) 50 mM glucose; (**c**) 50 mM glucose + DMSO; (**d**) 50 mM glucose + EPL 4 µM; (**e**) 50 mM glucose + DHP3 4 µM. Lenses exposed to high glucose showed increased opacity, whereas treatment with the evaluated compound and the reference inhibitor preserved lens transparency. Abbreviations: DMSO, dimethyl sulfoxide; EPL, epalrestat; DHP3, 1,4-dihydropyridine derivative 3.

To facilitate the visualization of opacity differences among experimental groups, representative lens images were processed in grayscale (Figure 4). Under these conditions, lenses with greater opacity displayed increased white regions, whereas more transparent lenses appeared predominantly dark. The hyperglycemic and DMSO-treated groups exhibited a larger proportion of white areas compared with the normoglycemic control group. In contrast, lenses treated with epalrestat or DHP3 showed darker image profiles, consistent with lower apparent opacity.

### 2.7. Quantitative Analysis of Lens Opacity

**Figure 5:**
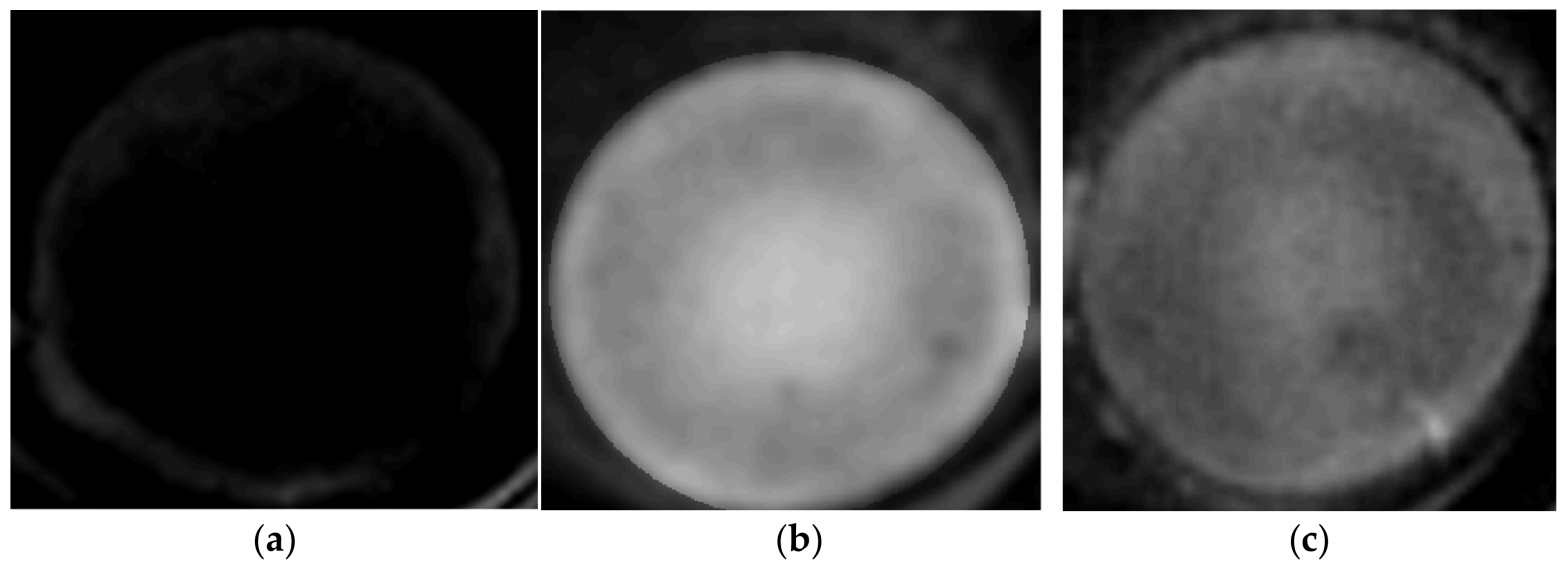

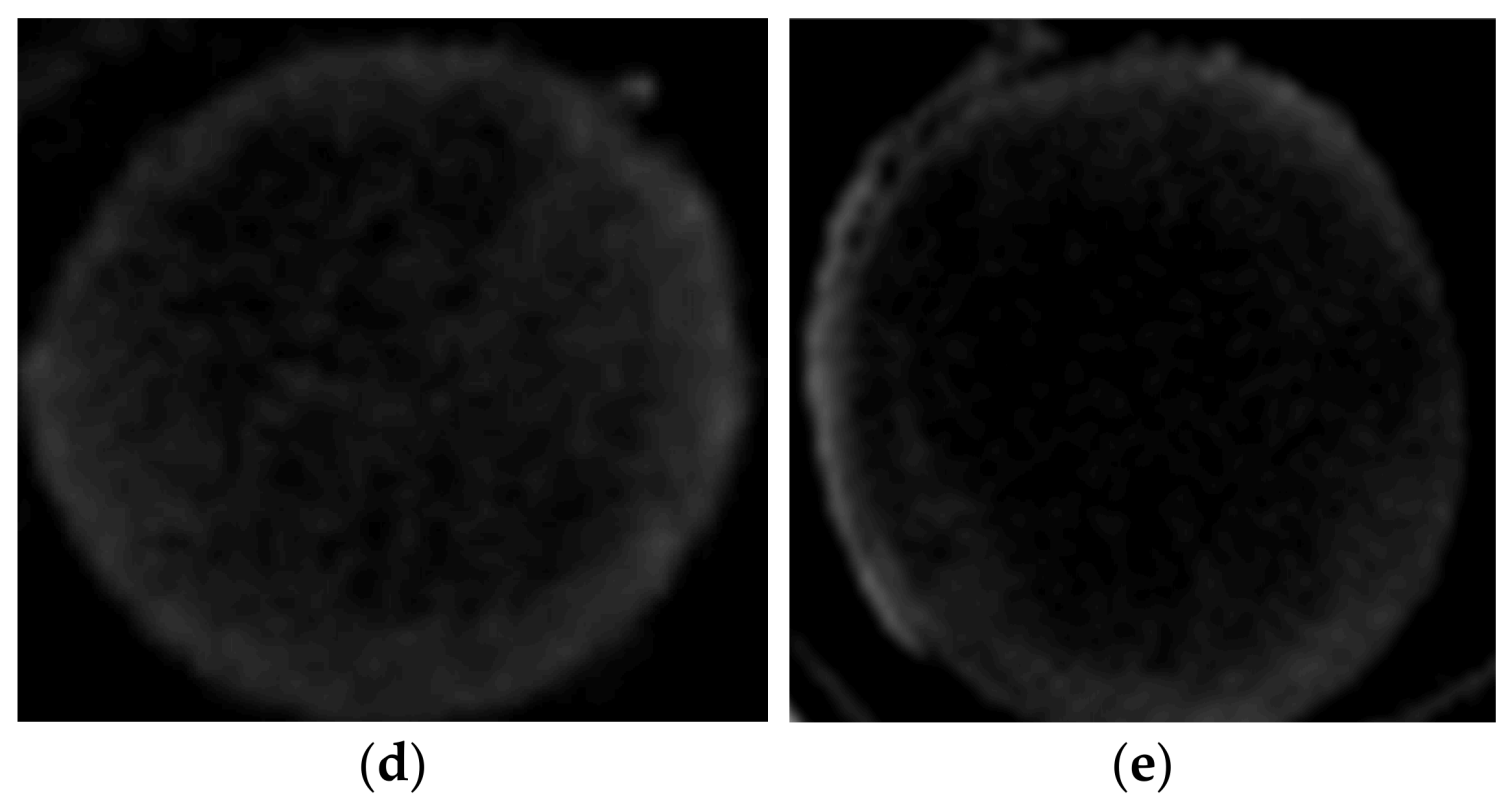
Representative 8-bit grayscale images generated in ImageJ for the quantitative analysis of lens opacity. (**a**) 5 mM glucose; (**b**) 50 mM glucose; (**c**) 50 mM glucose + DMSO; (**d**) 50 mM glucose + EPL 4 µM; (**e**) 50 mM glucose + DHP3 4 µM. Abbreviations: DMSO, dimethyl sulfoxide; EPL, epalrestat; DHP3, 1,4-dihydropyridine derivative 3.

**Figure 6:**
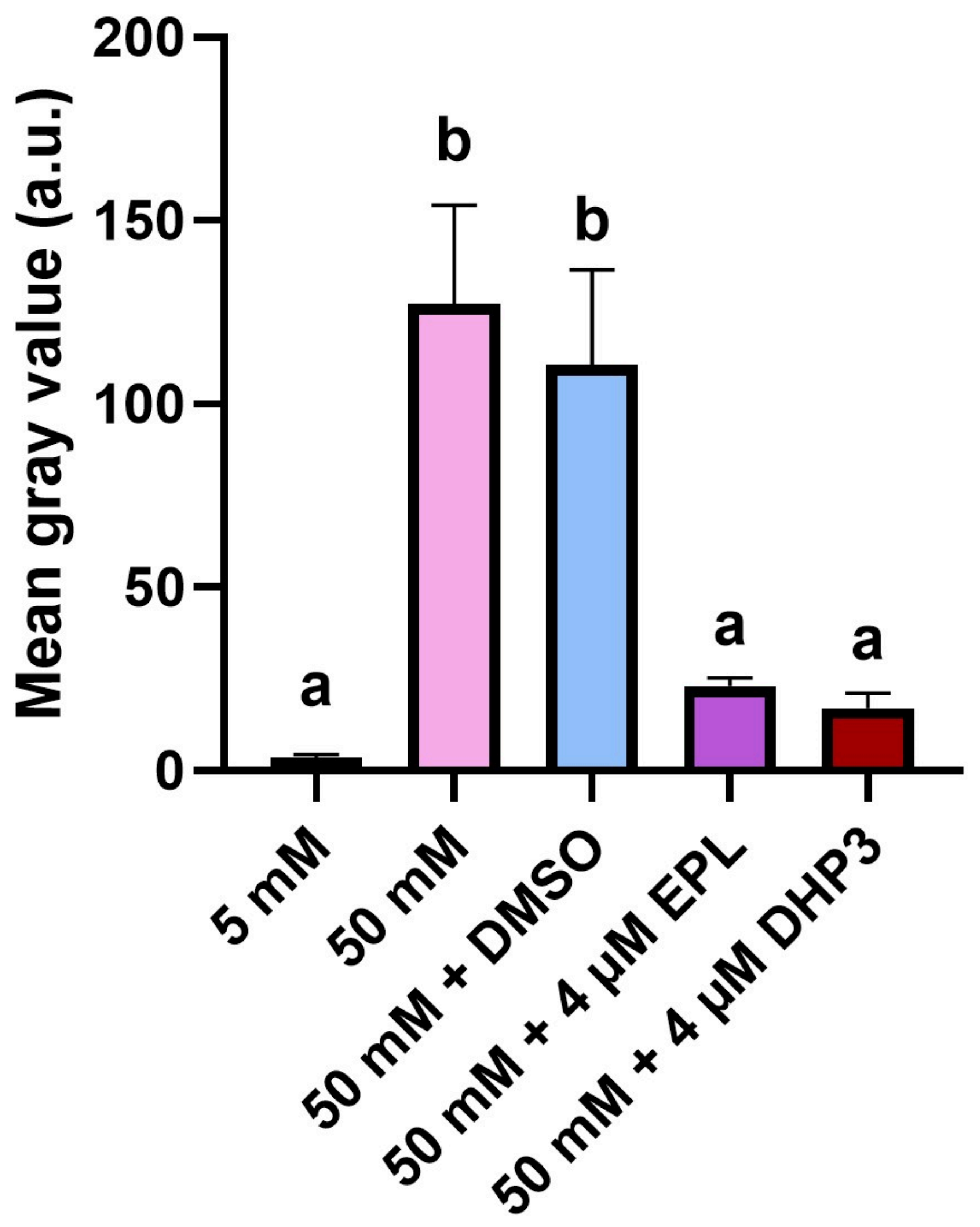
Quantification of lens opacity based on mean gray value (a.u.) after 6 days of culture under different experimental conditions. Hyperglycemic conditions were induced with 50 mM glucose. Higher mean gray values indicate greater lens opacity. Data are expressed as mean ± SD (*n* = 3). Statistical analysis was performed using one-way ANOVA followed by Tukey’s post hoc test. Different letters indicate statistically significant differences (*p* < 0.05). Given the limited number of biological replicates, the statistical comparisons are presented as exploratory. Abbreviations: DMSO, dimethyl sulfoxide; EPL, epalrestat; DHP3, 1,4-dihydropyridine derivative 3; a.u., arbitrary units.

Quantitative image analysis revealed differences in opacity values among the evaluated groups (Figure 5). Quantitative image analysis revealed differences in opacity values among the evaluated groups (Figure 6). Lenses incubated for 6 days with 50 mM glucose showed higher opacity values compared with the normoglycemic control group, whereas treatment with epalrestat or DHP3 reduced the measured opacity under hyperglycemic conditions. Among the evaluated treatments, the DHP3-treated group exhibited opacity values closer to those observed in the normoglycemic group.

### 2.8. Sorbitol Levels in Rabbit Lenses Under Hyperglycemic Conditions

**Figure 7:**
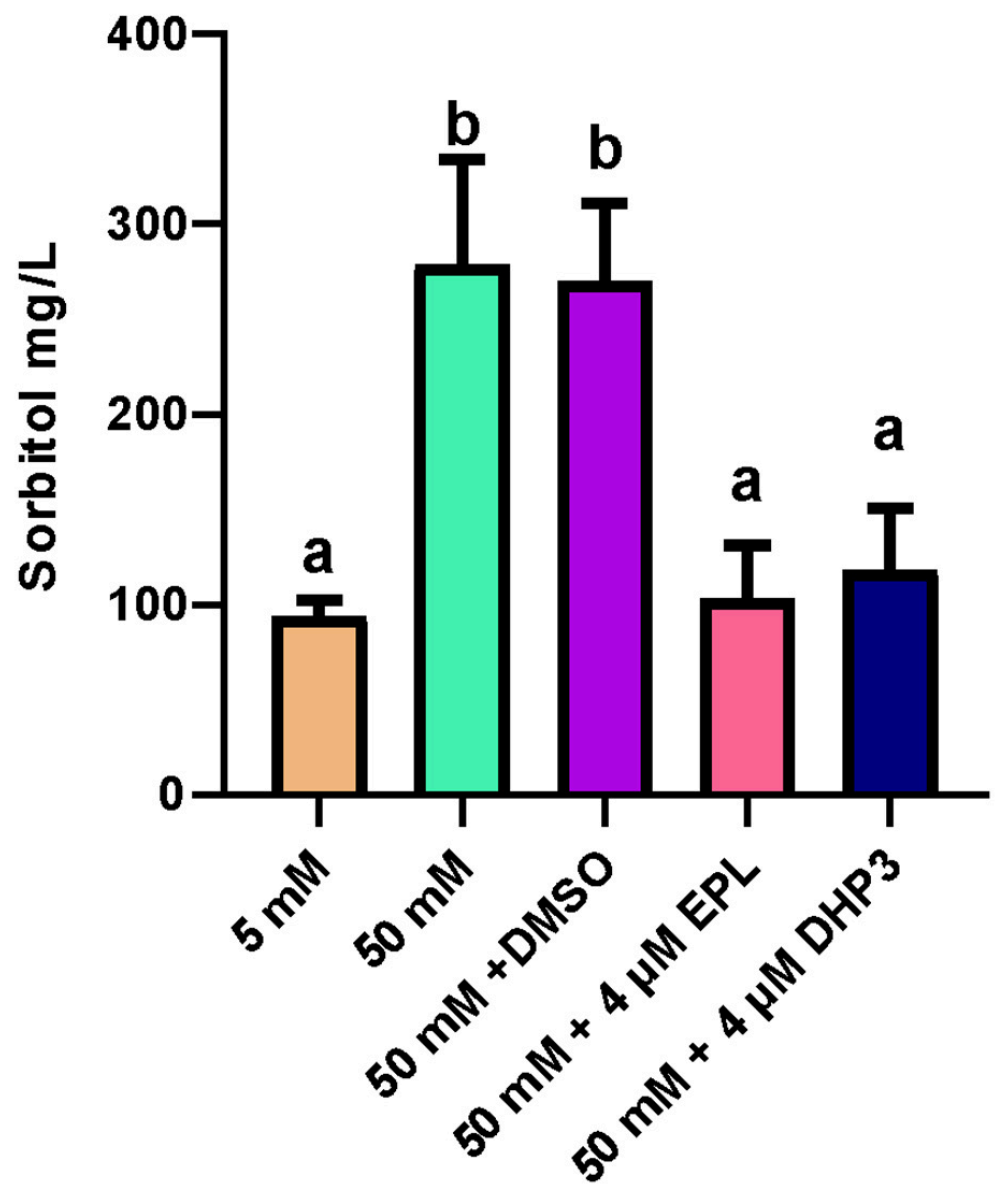
Effect of treatments on sorbitol levels in lenses after 6 days of culture under different experimental conditions. Hyperglycemic conditions were induced with 50 mM glucose. Data are expressed as mean ± SD (*n* = 3). Statistical analysis was performed using one-way ANOVA followed by Tukey’s post hoc test. Different letters indicate statistically significant differences (*p* < 0.05). Given the limited number of biological replicates, the statistical comparisons are presented as exploratory. Abbreviations: DMSO, dimethyl sulfoxide; EPL, epalrestat; DHP3, 1,4-dihydropyridine derivative 3.

Sorbitol levels in rabbit lenses differed among the evaluated experimental conditions (Figure 7). Lenses incubated for 6 days with 50 mM glucose showed increased sorbitol concentration compared with the normoglycemic control group (5 mM glucose). Similar values were observed in the DMSO-treated group. In contrast, lenses treated with epalrestat or *para*-fluorinated derivative DHP3 exhibited lower sorbitol levels under hyperglycemic conditions. Treatment with DHP3 at 4 µM reduced sorbitol accumulation by approximately 57.48% relative to the hyperglycemic control group (50 mM glucose). Among the evaluated treatments, the DHP3-treated group showed sorbitol values closer to those observed in the normoglycemic group.

### 2.9. Total Calcium Levels in Rabbit Lenses

**Figure 8:**
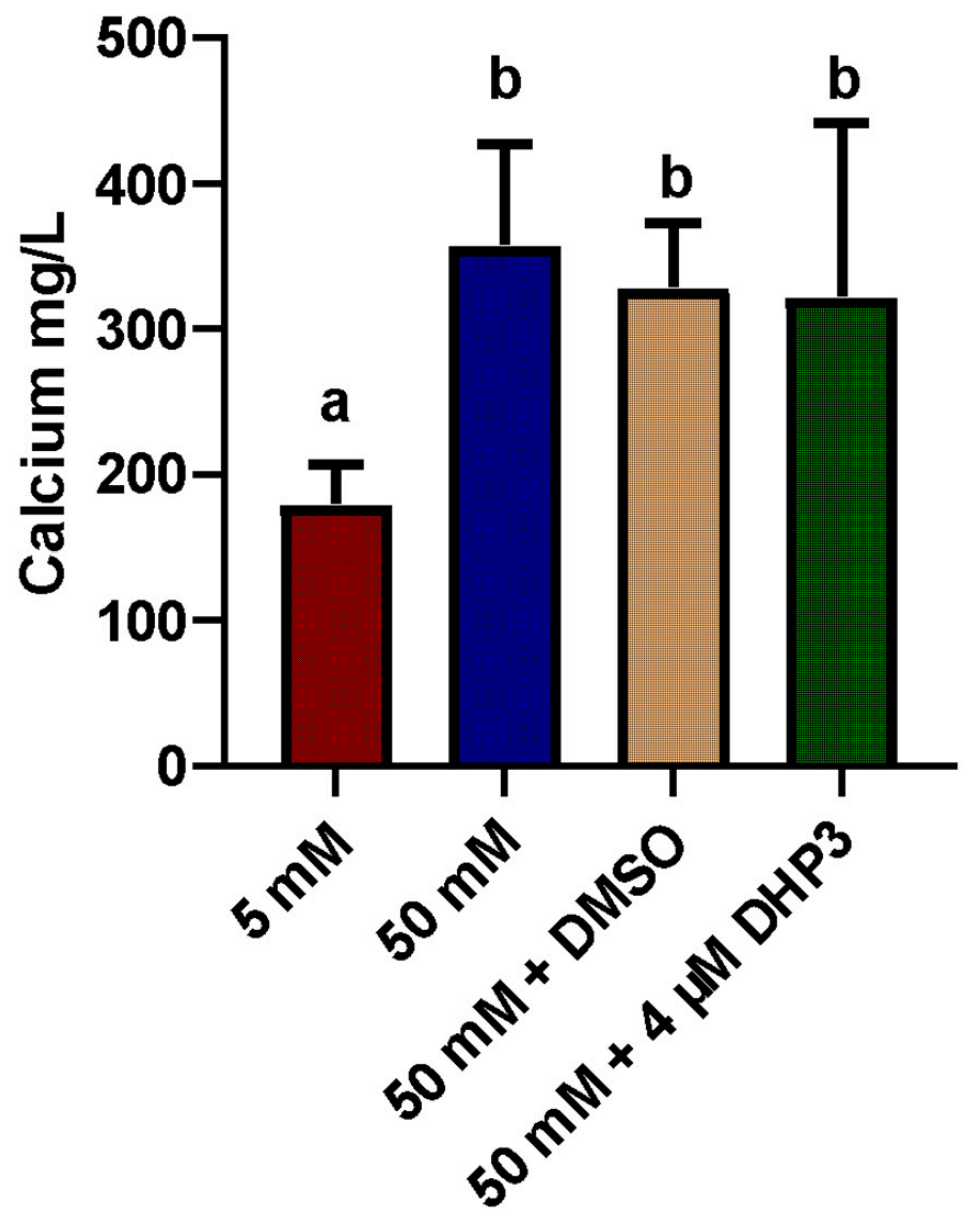
Effect of the 1,4-dihydropyridine derivative 3 DHP3 on calcium levels under experimental conditions. Data are expressed as mean ± SD (*n* = 3). Different letters indicate statistically significant differences (*p* < 0.05). Given the limited number of biological replicates, the statistical comparisons are presented as exploratory. Abbreviations: DMSO, dimethyl sulfoxide; DHP3, 1,4-dihydropyridine derivative 3.

Total calcium levels in rabbit lenses are shown in Figure 8. Lenses incubated under hyperglycemic conditions (50 mM glucose) exhibited higher calcium levels compared with the normoglycemic control group (5 mM glucose). Similar calcium values were observed in the DMSO and DHP3 treated groups under hyperglycemic conditions. Treatment with DHP3 at 4 µM resulted in a 2.07% reduction in total lens calcium relative to the hyperglycemic control group; however, this difference was not statistically significant. The mean calcium values were 328.94 mg/L in the hyperglycemic control group and 322.13 mg/L in the DHP3-treated group.

## 3. Discussion

Aldose reductase plays a key role in the polyol pathway, and its overactivation has been associated with diabetic complications such as cataract formation [14]. Despite significant efforts, the development of potent and selective aldose reductase inhibitors remains a challenging task due to the structural flexibility of its active site. In this study, we report a series of rationally designed 1,4-dihydropyridine derivatives evaluated as aldose reductase inhibitors through enzymatic assays, molecular docking and ex vivo lens model studies. Our results show that structural modifications on the aromatic ring significantly influence inhibitory potency, with fluorine-substituted derivatives exhibiting the highest activity. These findings support the potential of the DHP scaffold as a promising platform for the development of new agents targeting diabetic cataract. A more detailed analysis of the structure–activity relationship (SAR) revealed that the inhibitory activity of the synthesized 1,4-dihydropyridine derivatives is strongly influenced by both the electronic nature and steric properties of the substituents on the aromatic ring [17,20]. The unsubstituted compound (DHP1) exhibited moderate inhibitory activity (IC_50_ = 2.70 µM), serving as a reference for comparison. Notably, the introduction of a fluorine atom at the para position significantly enhanced inhibitory potency (IC_50_ = 1.11 µM), suggesting that small electron-withdrawing substituents favour enzyme binding. In contrast, bulkier substituents such as bromine led to reduced activity, particularly in the meta position (IC_50_ = 7.27 µM), likely due to steric hindrance and suboptimal orientation within the active site. Similarly, the introduction of an electron-donating methyl group at the meta position (DHP2) resulted in lower inhibition potency (IC_50_ = 31.32 µM) compared with DHP1, suggesting that inhibitory activity is influenced not only by the electronic nature of the substituent but also by its position and steric properties. These results highlight the combined influence of steric and electronic factors in determining aldose reductase inhibitory activity [20].

To further elucidate the inhibitory behavior of the synthesized compounds, kinetic studies were performed, and the inhibition mechanisms were analyzed using Lineweaver–Burk plots and corresponding Ki and Ki′ values. The reference compound (DHP1) exhibited a mixed-type inhibition pattern, as indicated by the intersection of the double-reciprocal plots and the different values of Ki (5.11 µM) and Ki′ (8.21 µM), suggesting that the inhibitor can bind both to the free enzyme and to the enzyme–substrate complex, with a slight preference for the free enzyme.

Among the substituted derivatives, the para-fluoride compound displayed a pronounced difference between Ki and Ki′ values (3.6 µM vs. 507.20 µM), indicating a predominantly mixed-type inhibition behavior and a preferential interaction with the free enzyme. This suggests that the presence of a small electron-withdrawing substituent favors access to or interaction within the catalytic site. In contrast, derivatives bearing bulkier substituents such as bromine showed nearly identical Ki and Ki′ values, consistent with a non-competitive inhibition mechanism, likely reflecting steric constraints that limit access to the active site and promote binding to alternative regions of the enzyme [19].

Similarly, meta-substituted derivatives predominantly exhibited non-competitive or mixed inhibition profiles, indicating that substitution at this position may alter ligand orientation and favor interactions outside the catalytic site. Overall, these findings demonstrate that structural modifications not only influence inhibitory potency but also modulate the mechanism of enzyme inhibition, highlighting the sensitivity of aldose reductase to both steric and positional effects. The inhibition patterns identified from the kinetic analyses provide a consistent basis for proposing the inhibition models of the synthesized compounds.

Although nonlinear regression is currently regarded as the preferred method for estimating enzyme kinetic parameters because it minimizes the bias introduced by reciprocal data transformations and enables the estimation of confidence intervals, Lineweaver-Burk plots continue to provide a useful graphical approach for distinguishing enzyme inhibition mechanisms. Therefore, the inhibition patterns described in this study were established using Lineweaver-Burk plots and secondary replots, and the reported Ki and Ki’ values should be interpreted considering the methodological limitations inherent to double-reciprocal analyses [21]. The apparent basal kinetic parameters determined under the present assay conditions were ^app^Km_gly_ = 0.37 mM and ^app^Vmax_gly_ = 0.01 mM·min^−1^. The apparent Km obtained for DL-glyceraldehyde was higher than values previously reported for human aldose reductase under other experimental conditions [22]. This difference may reflect methodological and assay-dependent factors, including enzyme source, substrate concentration range, cofactor concentration, pH, and ionic composition. In particular, sulfate ions have been reported to modulate aldose reductase activity and apparent kinetic parameters [23]. Therefore, the Km reported here should be interpreted as an apparent parameter specific to the experimental conditions employed, which included 50 mM Li_2_SO_4_, rather than as an intrinsic physiological constant of AKR1B1 [10]. Another relevant aspect for the further characterization of these compounds is selectivity within the AKR1B subfamily. AKR1B1 was selected as the primary target of this study because of its established involvement in the polyol pathway and its high expression in the human lens. AKR1B10 is also expressed in ocular tissues but shows a distinct distribution, with prominent expression in the cornea and lower expression in the lens compared with AKR1B1 [24]. Nevertheless, given the high structural similarity between AKR1B1 and AKR1B10 and the potential for cross-inhibition by aldose reductase inhibitors, selectivity between these enzymes remains an important consideration in the development of new AKR1B1 inhibitors [25,26]. Since the present study evaluated recombinant human AKR1B1 only, the selectivity of the DHP derivatives toward AKR1B1 over AKR1B10 remains to be experimentally determined [27]. This aspect may be particularly relevant for the future development of these compounds for topical ocular administration, where exposure of other ocular tissues, including the cornea, may occur. Future studies addressing AKR1B1/AKR1B10 selectivity will therefore be important to better define the pharmacological profile of these compounds. The predicted docking scores of the DHP derivatives ranged from −6.8 to −7.8 kcal/mol (−28.45 to −32.63 kJ/mol), indicating that all compounds were able to adopt energetically favorable poses within the aldose reductase binding cavity. However, no direct relationship was observed between the predicted docking scores and the experimentally determined IC_50_ values. DHP3, the para-fluorinated derivative and the most potent inhibitor in the enzymatic assay, exhibited the least favorable docking score within the DHP series (−6.8 kcal/mol). Conversely, several derivatives with lower experimental inhibitory potency displayed more negative predicted docking scores.

This lack of direct correlation should be interpreted considering the intrinsic limitations of conventional molecular docking. AutoDock Vina scoring functions provide an approximate estimation of ligand–receptor interactions within a largely rigid protein environment and are primarily intended to identify plausible binding poses rather than to quantitatively reproduce experimental inhibitory potency. Experimental IC_50_ values are additionally influenced by factors that are not fully represented in docking calculations, including protein flexibility, solvent effects, entropic contributions, ligand desolvation, and the kinetic behavior of the enzyme–inhibitor system. Therefore, the docking results obtained in the present study are better interpreted as qualitative structural evidence describing the possible binding orientation and interaction patterns of the DHP derivatives rather than as quantitative predictors of inhibitory potency [28]. The structural relevance of the interactions observed for the halogenated DHP derivatives should also be considered in the context of the architecture of the aldose reductase active site. The AR binding cavity comprises functionally distinct regions, including the conserved anion-binding region and the more flexible specificity pocket. Tyr48, His110, and Trp111 are located within the catalytic region close to the nicotinamide moiety of the cofactor, whereas Trp111 and Thr113 also contribute to regions involved in inhibitor recognition. Tyr48 plays a key catalytic role as a proton donor, while His110 contributes to substrate positioning within the active site. Consequently, interactions involving these residues may be particularly relevant for the orientation and stabilization of inhibitors within catalytically important regions of the enzyme [29]. Despite exhibiting the least favorable docking score within the DHP series, DHP3 established two predicted hydrogen-bond interactions with Tyr48 and Trp111, both located within the aldose reductase active-site region. These interactions may favor the orientation and stabilization of DHP3 within the binding pocket and may contribute to its experimentally observed inhibitory potency. DHP7, bearing a meta-chloro substituent, also displayed predicted hydrogen-bond interactions involving His110 and Trp111. In contrast, the other halogenated derivatives did not reproduce the same interaction pattern with these residues in their best-ranked poses. No prominent interaction with Thr113 was identified among the selected poses. These observations indicate that the effects of halogen substitution are dependent not only on the identity of the halogen but also on its position on the aromatic ring.

From a structural perspective, halogen substitution may influence ligand recognition by modifying hydrophobicity, polarizability, van der Waals complementarity, and the electronic distribution of the substituted aromatic ring. Such effects may alter the orientation of the DHP scaffold within the binding pocket and consequently determine which residues participate in ligand stabilization. The distinct interaction patterns observed for the para-fluorinated DHP3 and meta-chloro DHP7 are consistent with this interpretation. Nevertheless, the present docking analysis does not provide sufficient geometric evidence to assign classical halogen bonds to the evaluated derivatives. Therefore, the contribution of fluorine, chlorine, and bromine is interpreted primarily in terms of their influence on local ligand–protein complementarity, binding geometry, and non-covalent interaction networks rather than as direct evidence of specific halogen-bond formation.

The kinetic and docking results should therefore be considered complementary rather than directly equivalent. Several DHP derivatives exhibited mixed or non-competitive inhibition patterns, indicating that their kinetic behavior cannot be explained solely by simple competition with the substrate. However, because the revised docking calculations were performed within the validated AR–NADP active-site region, they do not directly demonstrate binding to peripheral or allosteric sites. Instead, the docking analysis shows that structurally related DHP derivatives can establish distinct interaction networks within the binding pocket. These findings suggest that inhibitory potency is determined by a combination of substituent position, steric and electronic properties, specific residue interactions, and enzyme–inhibitor kinetic behavior rather than by docking score alone [30].

The ex vivo lens model provided further evidence of the biological relevance of the evaluated compounds [31]. Visual inspection of the lenses revealed clear differences in transparency among the treatment groups. Lenses exposed to the control conditions exhibited increased opacity, consistent with protein aggregation and cataract formation, whereas those treated with the para-fluorinated derivative (DHP3) maintained a noticeably higher degree of transparency. This effect was comparable to that observed with the reference compound, EPL, suggesting a protective role of DHP3 against lens opacification.

These observations were further supported by quantitative image analysis using ImageJ, which confirmed that the transparency levels of lenses treated with DHP3 were similar to those of the EPL-treated group. This ex vivo system reflects a more complex biological environment than in vitro enzymatic assays, in which factors such as compound diffusion, tissue interactions, and the local microenvironment can influence activity. Therefore, DHP3′s ability to preserve lens transparency supports its potential as a promising candidate for preventing cataract formation associated with diabetes.

In correlation with the visual and kinetic findings, sorbitol levels in lens tissue were significantly reduced in the presence of the para-fluorinated derivative (DHP3), reaching values comparable to those observed with the reference inhibitor, epalrestat. Given that sorbitol accumulation is a hallmark of aldose reductase overactivation within the polyol pathway [16,17], its reduction strongly supports effective enzyme inhibition under ex vivo conditions. The decrease in sorbitol content suggests that DHP3 mitigates osmotic stress within the lens, thereby preventing water influx, protein aggregation, and subsequent loss of transparency [14]. This biochemical evidence complements the observed preservation of lens clarity and reinforces the proposed mechanism of action of DHP3 as an aldose reductase inhibitor. The ex vivo experiments provided evidence under the evaluated conditions. Given the limited number of biological replicates (*n* = 3 per group) and the evaluation of DHP3 at a single concentration, the statistical analysis should be considered exploratory. Further studies with larger sample sizes and concentration–response designs will be important to confirm and extend these findings.

This in vitro study guided us toward the initial selection of DHPs with aldose reductase inhibitory properties and cataract prevention potential. However, a mechanistic understanding of this catalytic and physiological process, as well as the disposition of drug-like DHPs and their potential pleiotropic deleterious effects, is of paramount interest for the advancement of toxicological evaluations. A prediction of key properties associated with ADME parameters was made (see Tables S1–S5). Preliminary ADME analysis indicated that the DHPs tested in this work exhibit parameters characteristic of a compound with drug-like activity. In addition to the predicted ADME properties, the metabolic behavior of the 1,4-dihydropyridine scaffold should also be considered. Previous studies have shown that DHP derivatives can undergo microsomal oxidative metabolism mediated by cytochrome P450 enzymes, particularly CYP3A4, and that some 1,4-dihydropyridines can also inhibit CYP-dependent reactions [32]. Related DHP derivatives previously reported by Charlton et al. were also shown to undergo microsomal oxidation, with structural substitution influencing their oxidation behavior [33]. Therefore, experimental evaluation of microsomal stability, CYP-mediated metabolism, and potential CYP inhibition will be important for the subsequent pharmacological characterization of the compounds evaluated in the present study.

Regarding total calcium content, levels remained essentially unchanged among the experimental groups included in the calcium analysis (50 mM, 50 mM + DMSO, and 50 mM + DHP3). This indicates that the protective effect observed with DHP3 was not accompanied by detectable changes in total lens calcium content under the evaluated conditions. However, the possibility that DHP3 exerts an additional, untargeted mechanism in lens cells that acts synergistically with its inhibitory effect on aldose reductase should be explored. Calcium imbalance has been implicated in lens opacification through activation of proteolytic enzymes and structural disruption [34,35].

The absence of significant changes suggests that DHP3 does not exert its effects through calcium-dependent pathways. Instead, this observation further supports that its primary mechanism of action is linked to modulation of the polyol pathway via aldose reductase inhibition, rather than secondary ionic disturbances. Nevertheless, oxidative stress is another major contributor to diabetic cataract formation and was not evaluated in the present study. Therefore, future studies should investigate oxidative stress biomarkers, including reactive oxygen species (ROS), glutathione (GSH), lipid peroxidation, and protein oxidation, to determine whether DHP3 also exerts protective effects through modulation of oxidative damage.

## 4. Materials and Methods

### 4.1. Chemicals and Reagents

All chemicals and reagents were purchased from Sigma Chemical Co. (St. Louis, MO, USA). TheD-Sorbitol/Xylitol Assay Kit was purchased from Megazyme International Ireland Ltd., Wicklow, Ireland.

### 4.2. Synthesis of DHP Derivatives

1,4-Dihydropyridine derivatives (DHP1–DHP9) were synthesized through a modified Hantzsch reaction according to previously reported procedures [18,19,36,37]. Briefly, substituted benzaldehydes, ethyl 3-aminocrotonate, and ethyl acetoacetate were mixed at a 1:1:1 molar ratio in isopropanol (5 mL) in a 50 mL round-bottom flask. The reaction mixture was refluxed under magnetic stirring for 20–24 h. After completion, the mixture was cooled to room temperature until precipitation occurred. The resulting solids were filtered, washed with cold methanol, recrystallized from methanol, and dried at room temperature.

### 4.3. Nuclear Magnetic Resonance Spectroscopy

The synthesized 1,4-dihydropyridine derivatives (DHP1–DHP9) were characterized by ^1^H and ^13^C nuclear magnetic resonance (NMR) spectroscopy. NMR spectra were acquired on a Varian Mercury 300 spectrometer (Varian, Inc., Palo Alto, CA, USA) equipped with a 5-mm inverse probe at 25 °C, using CDCl_3_ as the deuterated solvent. ^1^H and ^13^C NMR spectra were recorded at 300 and 75 MHz, respectively. Chemical shifts (δ) are reported in parts per million (ppm), and coupling constants (J) are reported in hertz (Hz). Complete ^1^H and ^13^C NMR spectra and the corresponding spectroscopic data for DHP1-DHP9 are provided in the Supplementary Materials (see Figures S1–S18).

### 4.4. Purity Analysis by HPLC-DAD

Purity analysis was performed by HPLC-DAD using an Agilent Technologies 1200 Infinity Series system (Agilent Technologies, Inc., Santa Clara, CA, USA) equipped with a quaternary pump, autosampler, and diode array detector. Chromatographic separation was achieved using an Ascentis Express C18 analytical column (250 × 4.6 mm, 5 µm; Supelco, Bellefonte, PA, USA) maintained at 35 °C. The mobile phase consisted of methanol and 1% aqueous formic acid under isocratic elution conditions at a flow rate of 0.8 mL min^−1^. The injection volume was 2 µL, and detection was carried out at 235–238 nm, corresponding to the absorption maximum of the 1,4-dihydropyridine chromophore. UV spectra were recorded between 200 and 400 nm to assess peak purity. Samples were prepared in methanol, protected from light, and filtered through 0.22 µm membrane filters prior to injection. Purity values ranged from 94.23% to 99.3%. Detailed spectroscopic characterization data are provided in the Supplementary Materials (Figures S19–S27).

### 4.5. Aldose Reductase Activity Assay

Aldose reductase activity was determined spectrophotometrically by monitoring the oxidation of NADPH at 340 nm, as previously described by Hayman and Kinoshita [38]. Recombinant human aldose reductase (hrAR; SRP6371, Sigma-Aldrich, St. Louis, MO, USA; purity ≥ 95% by SDS-PAGE; specific activity: 166 U/mg protein) was used in all experiments. The enzyme was stored at −80 °C until use and thawed immediately before each experiment. The reaction mixture (final volume, 1 mL) contained 0.10 M potassium phosphate buffer (pH 6.8), 2 mM NADPH, 50 mM lithium sulfate, and 100 mM DL-glyceraldehyde and recombinant human aldose reductase at a final concentration of 0.00016 mg/mL, corresponding to 0.026 U/mL. The corresponding final concentrations during spectrophotometric monitoring were approximately 65 mM potassium phosphate buffer, 15 mM lithium sulfate, 0.20 mM NADPH, and 20 mM DL-glyceraldehyde. Assays were performed at 37 °C. After a 3 min pre-incubation, the reaction was initiated by the addition of glyceraldehyde. The decrease in absorbance at 340 nm was recorded for 3 min using a UV–visible spectrophotometer (Cary 50 Bio, Varian, Inc., Palo Alto, CA, USA). Initial reaction rates (V_0_) were calculated from the linear portion of the absorbance–time curve (ΔAbs/min). When required, rates were converted to µmol NADPH min^−1^ using an extinction coefficient of 6.22 mM^−1^ cm^−1^. DHP compounds were prepared in DMSO and added to the reaction mixture to achieve the desired final inhibitor concentrations, while maintaining a constant DMSO concentration of 1% (*v*/*v*) in all assays. Stock solutions of the DHP derivatives were freshly prepared in DMSO on the day of each experiment and diluted immediately before use. Control reactions containing 1% (*v*/*v*) DMSO without inhibitor were included to account for solvent effects. A reaction mixture without enzyme was used as a blank.

### 4.6. Determination of IC_50_

The inhibitory activity of 1,4-dihydropyridine derivatives against aldose reductase was evaluated by determining the half-maximal inhibitory concentration (IC_50_). Enzyme activity was measured under the assay conditions described above using inhibitor concentrations ranging from 0.04 to 40 µM for most compounds. Percentage inhibition was calculated relative to the control reaction, defined as 100% enzyme activity in the presence of 1% DMSO without inhibitor. Dose–response curves were fitted by nonlinear regression analysis using Microsoft Excel (version 2610; Microsoft Corporation, Redmond, WA, USA). IC_50_ values are expressed as mean ± SD from three independent experiments, with each condition measured in technical triplicate.

### 4.7. Enzyme Kinetics and Inhibition Analysis

Enzyme kinetics and inhibition studies were performed under the same experimental conditions described for the aldose reductase activity assay. Substrate concentrations were used to obtain the baseline AR parameters, and the baseline Km value is included within this concentration range. Substrate saturation experiments were carried out using DL-glyceraldehyde concentrations of 0.02, 0.04, 0.08, 2.0, 4.0, and 20.0 mM for DHP1, DHP3, and DHP8, and 0.5, 1.0, 2.0, 10.0, and 20.0 mM for the remaining 1,4-dihydropyridine derivatives, in the absence and presence of the corresponding inhibitor. Inhibitor concentrations were selected according to the inhibitory potency of each compound to allow appropriate kinetic evaluation. Double-reciprocal Lineweaver–Burk plots (1/V versus 1/[S]) were constructed for graphical interpretation of inhibition behavior. Kinetic measurements were performed in three independent experiments (*n* = 3) for each substrate and inhibitor concentration, and reciprocal reaction-rate values (1/V) are presented as mean ± standard error of the mean (SEM). Linear regression analysis was performed using Microsoft Excel to estimate apparent Km and Vmax values. Inhibition patterns were interpreted from the Lineweaver–Burk plots obtained at different inhibitor concentrations.

### 4.8. Determination of Ki

Inhibition constants (Ki) were calculated according to the method described by Segel [39]. Secondary plots of the slopes obtained from the Lineweaver–Burk analysis versus inhibitor concentration were constructed, and Ki values were estimated from the corresponding linear regressions.

### 4.9. Molecular Docking Analysis

Molecular docking analysis was performed using AutoDock Vina (version 1.1.2) [40]. The crystal structure of human aldose reductase complexed with the nicotinamide cofactor and the co-crystallized inhibitor minalrestat was retrieved from the Protein Data Bank (PDB ID: 1PWL). The co-crystallized minalrestat ligand was removed from the active site, whereas the NADP cofactor was retained, generating the AR–NADP binary complex used as the receptor for docking calculations.

The 1,4-dihydropyridine derivatives were constructed using the Maestro Elements software package (Schrödinger Release 2020–3). Prior to docking, the molecular geometries of the DHP derivatives were optimized at the B3LYP/6–311G+(d,p) level of theory using Gaussian 16 software. Vibrational frequency calculations were performed to confirm that the optimized structures corresponded to energy minimum. Ligands were subsequently prepared according to their protonation states at pH 7.4. Epalrestat was included as a reference aldose reductase inhibitor.

Docking calculations were performed using a search space centered at X = 17.38, Y = 10.61, and Z = −11.96, with grid-box dimensions of 10.50 × 12.00 × 9.00 Å. The same receptor, search space, and docking parameters were used for all evaluated ligands. AutoDock Vina generated multiple binding poses for each ligand, and the best-ranked pose, corresponding to the most favorable (most negative) predicted docking score, was selected for subsequent analysis. Three-dimensional visualization and comparison of selected ligand poses within the AR binding site were performed using UCSF ChimeraX version 1.12. Protein–ligand interaction patterns, including hydrogen-bond, hydrophobic, π-mediated, van der Waals, and other predicted contacts with residues surrounding the binding pocket, were analyzed and represented as two-dimensional interaction diagrams using Discovery Studio Visualizer (version 19.1.0.18287) [41].

The docking protocol was validated by redocking the co-crystallized inhibitor minalrestat into the unoccupied active site of the AR–NADP binary complex. Minalrestat was extracted directly from the 1PWL crystal structure and prepared for docking. Redocking was performed using the same receptor, search space, and docking parameters applied to the evaluated compounds. The crystallographic pose and the best-ranked redocked pose were exported as independent structure files and compared using UCSF ChimeraX version 1.12. The root-mean-square deviation (RMSD) between the corresponding ligand atoms was subsequently calculated.

### 4.10. Ex Vivo Lens Isolation

The anticataract assay was performed as previously described by Beltrán-Sánchez et al. [42]. Ten New Zealand rabbits (three months old) were obtained from a commercial meat production facility. Prior to slaughter, animal transportation and handling were conducted in accordance with the Mexican Official Standard NOM-051-ZOO-1995 [43], which establishes requirements for the humane treatment of animals during transportation and with the Guide for the Care and Use of Agricultural Animals in Research and Teaching. Slaughter and tissue collection were performed at the slaughterhouse of the Faculty of Veterinary Medicine of the Universidad Michoacana de San Nicolás de Hidalgo, following the Mexican standard NOM-033-SAG/ZOO-2014 [44] and the guidelines of the U.S. National Research Council [45]. Immediately after death, eyes (*n* = 20) were enucleated from the carcasses under aseptic conditions using sterile dissecting instruments. The eyeballs were placed in a phosphate buffer (pH 7.0) at 4 °C and transported to the laboratory. Lenses were carefully isolated under aseptic conditions in a laminar flow hood. For the experimental assays, three independent lenses were used per group (*n* = 3), each obtained from a different animal. Lenses were randomly assigned to the corresponding treatment groups.

### 4.11. Lens Culture and Cataract Induction

Lens were cultured in Medium 199 (Sigma-Aldrich, M3769; Sigma-Aldrich, St. Louis, MO, USA) supplemented with 1% penicillin–streptomycin, 0.50 mg/mL neomycin, 2.50 µg/mL amphotericin B, and sodium bicarbonate as a pH buffer. Lenses were assigned to the following experimental groups: (i) control group, (ii) 50 mM glucose-treated group to induce experimental cataract, (iii) 4 µM epalrestat as a positive control, and (iv) 4 µM DHP3-treated group. All lenses were incubated at 37 °C in a humidified atmosphere of 5% CO_2_ and 95% air for 6 days. The culture medium was renewed every 48 h. At the end of the incubation period, lens images were acquired using a Gel Doc 200 imaging system (Bio-Rad Laboratories, Hercules, CA, USA) under white light illumination with a uniform white background. All images were acquired under identical exposure conditions. Images were standardized for size and analyzed using ImageJ software (version 1.53t; National Institutes of Health, Bethesda, MD, USA). Lens opacity was quantified by measuring pixel intensity from the processed images. All experiments were performed using three independent lenses per group (*n* = 3), each obtained from different animals.

### 4.12. Lens Opacity Quantification by Image Analysis

Lens opacity was quantified using ImageJ software (version 1.53t; National Institutes of Health, Bethesda, MD, USA). Images were converted to 8-bit grayscale. A circular region of interest (ROI) encompassing the entire lens was defined according to standardized selection criteria for all images. The mean gray value (pixel intensity) within the ROI was measured as a quantitative parameter of lens opacity. All images were acquired under identical illumination, exposure, and black background conditions, and the same image-processing workflow was applied to all images to ensure comparability.

### 4.13. Sorbitol Assay

Sorbitol levels in rabbit lenses were determined using a D-Sorbitol/Xylitol Assay Kit (Megazyme International Ireland Ltd., Wicklow, Ireland), following the manufacturer’s instructions. Briefly, lenses were homogenized in a phosphate buffer containing a protease inhibitor cocktail and centrifuged at 13,000 rpm for 5 min at 4 °C. The supernatant was collected and used for sorbitol quantification according to the kit protocol.

### 4.14. Calcium Quantification in Rabbit Lenses

After 6 days of treatment, rabbit lenses were individually collected and washed three times with Ca^2+^- and Mg^2+^-free phosphate-buffered saline (PBS) to remove residual culture medium. Each lens was gently blotted dry and weighed to obtain fresh tissue weight. Lenses were mechanically homogenized using a Potter–Elvehjem homogenizer and transferred to acid-washed digestion tubes. Acid digestion was performed using nitric acid and hydrogen peroxide, followed by incubation at 90 °C for 6 h until complete tissue digestion was achieved. After cooling to room temperature, samples were diluted to a final volume of 5 mL with ultrapure water. Calcium concentration was determined by atomic absorption spectrometry using an external calibration curve prepared with calcium standards. Results were expressed as mg/L for graphical representation. When required, calcium content was also calculated per lens and normalized to fresh tissue weight (µg Ca/mg fresh weight). Blank digestions were included in each analytical run to control for background contamination. All measurements were performed in triplicate (*n* = 3).

### 4.15. Statistical Analysis

The comparison between the treatments for various analytical endpoints was analyzed using one-way ANOVA followed by Tukey’s post hoc test, and the results are presented as mean ± SD. For the ex vivo experiments, *n* = 3 represents three independent biological replicates (rabbit lenses obtained from different animals). For each lens, nine technical measurements were obtained and averaged to generate a single biological replicate value used for statistical analysis. For the enzyme kinetic analyses, experiments were performed on three independent occasions (*n* = 3), and the reciprocal reaction-rate values (1/V) presented in the Lineweaver–Burk plots are expressed as mean ± standard error of the mean (SEM). The normality of residuals from the one-way ANOVA models was assessed using the Shapiro–Wilk test. Statistical significance was defined as a *p*-value ≤ 0.05. IC_50_ for DHPs and controls standards was calculated by dose–response curves. All the statistical analysis and scientific graphing were carried out by Statistics 7.0, whereas the Shapiro–Wilk normality test was performed using GraphPad Prism 8.0.

## 5. Conclusions

The present study demonstrates that structural modifications of the 1,4-dihydropyridine scaffold significantly influence both the inhibitory potency and mechanism against aldose reductase. Among the evaluated compounds, the para-fluorinated derivative (DHP3) exhibited the most promising profile, combining effective enzymatic inhibition, a defined kinetic mechanism, and a protective effect in the ex vivo lens model comparable to epalrestat. The reduction in sorbitol levels, together with the preservation of lens transparency and the absence of alterations in calcium homeostasis, supports a mechanism primarily associated with modulation of the polyol pathway. These findings highlight the potential of fluorinated DHP derivatives as promising candidates for further development in the prevention of diabetic cataract.

## Figures and Tables

**Figure 1:**
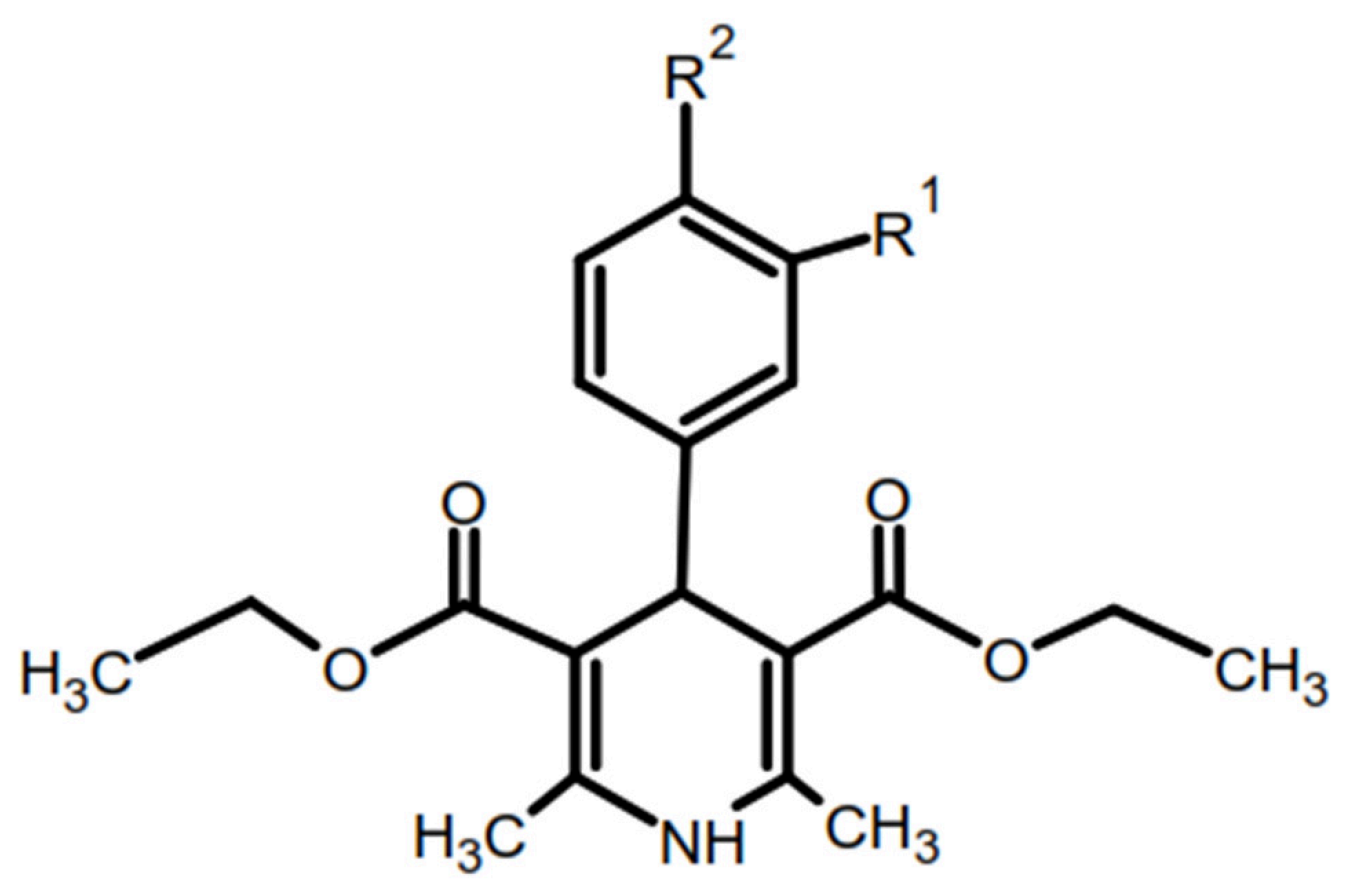
Chemical structure of 1,4-dihydropyridine derivatives (DHP1–DHP9).

**Table 1:** Structure and physical data of 1,4-dihydropyridine derivatives (DHPs).

DHP	R1	R2	Purity (%)
1	H	H	99.10
2	CH_3_	H	98.90
3	H	F	98.87
4	H	Cl	99.29
5	NO_2_	H	98.09
6	H	Br	94.23
7	Cl	H	99.02
8	OCH_3_	H	98.89
9	Br	H	98.08

**Table 2:** The half-maximal inhibitory concentration (IC_50_), inhibition constant (Ki) values, and inhibition types for 1,4-dihydropyridine derivatives (DHP1–DHP9).

DHP	R	IC_50_ (µM)	K_i_ (µM)	K_i’_ (µM)	Type of Inhibition
1	H	2.70 ± 0.32	5.11 ± 1.97	8.21 ± 3.58	Mixed
2	m-CH_3_	31.32 ± 0.12	11.17 ± 0.77	26.59 ± 4.56	Mixed
3	p-F	1.11 ± 0.07	3.6 ± 0.66	507.20 ± 189.78	Mixed
4	p-Cl	22.59 ± 0.05	39.38 ± 7.34	46.15 ± 19.96	Mixed
5	m-NO_2_	55.34 ± 0.06	10.14 ± 2.14		Non-competitive
6	p-Br	4.20 ± 0.76	13.42 ± 7.54		Non-competitive
7	m-Cl	2.44 ± 0.08	5.90 ± 2.03	24.10 ± 2.15	Mixed
8	m-OCH_3_	33.20 ± 0.11	77.35 ± 41.05	95.36 ± 48.11	Mixed
9	m-Br	7.27 ± 4.56	16.12 ± 7.48		Non-competitive

IC_50_ values are expressed as mean ± SD from three independent experiments, with each condition measured in technical triplicate.

**Table 3:** Molecular docking binding energies and key residue interactions of DHPs with aldose reductase. AutoDock Vina (version 1.1.2) binding affinity values are reported in their original units (kcal/mol) and converted to kJ/mol (1 kcal/mol = 4.184 kJ/mol; kcal/mol values were multiplied by 4.184).

DHP	AutoDock Vina Binding Affinity (kcal/mol)	Converted Binding Affinity (kJ/mol)	H-Bonds	Key Residues
1	−7.5	−31.38	-	-
2	−7.8	−32.63	1	Cys 298
3	−6.8	−28.45	2	Tyr 48, Trp 111
4	−7.2	−30.12	-	-
5	−7.4	−30.96	-	-
6	−7.4	−30.96	-	-
7	−7.5	−31.38	2	His 110, Trp 111
8	−7.3	−30.54	-	-
9	−7.7	−32.21	-	-
EPL	−9.8	−41.00	3	Trp 20, Tyr 48, His 110

## Data Availability

The original contributions presented in this study are included in the article/Supplementary Material. Further inquiries can be directed to the corresponding authors.

## Supplementary Materials

The following supporting information can be downloaded at: https://www.mdpi.com/article/10.3390/ijms27219642/s1.

## Abbreviations

The following abbreviations are used in this manuscript:

DHP	Dihydropyridine Multidisciplinary Digital Publishing Institute
IC_50_	Half maximal inhibitory concentration
^13^CNMR	Carbon Nuclear Magnetic Resonance
^1^HNMR	Hydrogen Nuclear Magnetic Resonance
DMSO	Dimethyl sulfoxide
Ki	Inhibition constant
EPL	Epalrestat
a.u.	Arbitrary units
Å	Angstrom
