# Supplementary material for: Evaluation of Dihydropyridine Derivatives as Aldose Reductase Inhibitors: Enzymatic Screening, Molecular Docking and Ex Vivo Assessment of a Selected Compound in a Lens Model
Source: Int J Mol Sci. 2026 Oct 28;27(21):9642. doi: 10.3390/ijms27219642 (PMC13762734; doi:10.3390/ijms27219642)
Supplement: Supplementary file 1 [file ijms-27-09642-s001.zip › ijms-4599155-supplementary.pdf]

Supplementary Material

Evaluation of Dihydropyridine Derivatives as Aldose Reductase Inhibitors: Enzymatic Screening, Molecular Docking and Ex Vivo Assessment of a Selected Compound in a Lens Model

Table of Contents

Figure	Caption	Page
**S1**	DHP1: [Diethyl 2,6-dimethyl-4-phenyl-1,4-dihydropyridine-3,5-dicarboxylate] ^{1}H NMR spectrum	1
**S2**	DHP1: [Diethyl 2,6-dimethyl-4-phenyl-1,4-dihydropyridine-3,5-dicarboxylate] ^{13}C NMR spectrum	2
**S3**	DHP2: [Diethyl 2,6-dimethyl-4-(3-methylphenyl)-1,4-dihydropyridine-3,5-dicarboxylate] ^{1}H NMR spectrum	3
**S4**	DHP2: [Diethyl 2,6-dimethyl-4-(3-methylphenyl)-1,4-dihydropyridine-3,5-dicarboxylate] ^{13}C NMR spectrum	4
**S5**	DHP3: [Diethyl 2,6-dimethyl-4-(4-fluorophenyl)-1,4-dihydropyridine-3,5-dicarboxylate] ^{1}H NMR spectrum	5
**S6**	DHP3: [Diethyl 2,6-dimethyl-4-(4-fluorophenyl)-1,4-dihydropyridine-3,5-dicarboxylate] ^{13}C NMR spectrum	6
**S7**	DHP4: [Diethyl 2,6-dimethyl-4-(4-chlorophenyl)-1,4-dihydropyridine-3,5-dicarboxylate] ^{1}H NMR spectrum	7
**S8**	DHP4: [Diethyl 2,6-dimethyl-4-(4-chlorophenyl)-1,4-dihydropyridine-3,5-dicarboxylate] ^{13}C NMR spectrum	8
**S9**	DHP5: [Diethyl 2,6-dimethyl-4-(3-nitrophenyl)-1,4-dihydropyridine-3,5-dicarboxylate] ^{1}H NMR spectrum	9
**S10**	DHP5: [Diethyl 2,6-dimethyl-4-(3-nitrophenyl)-1,4-dihydropyridine-3,5-dicarboxylate] ^{13}C NMR spectrum	10
**S11**	DHP6: [Diethyl 2,6-dimethyl-4-(4-bromophenyl)-1,4-dihydropyridine-3,5-dicarboxylate] ^{1}H NMR spectrum	11
**S12**	DHP6: [Diethyl 2,6-dimethyl-4-(4-bromophenyl)-1,4-dihydropyridine-3,5-dicarboxylate] ^{13}C NMR spectrum	12
**S13**	DHP7: [Diethyl 2,6-dimethyl-4-(3-chlorolphenyl)-1,4-dihydropyridine-3,5-dicarboxylate] ^{1}H NMR spectrum	13
**S14**	DHP7: [Diethyl 2,6-dimethyl-4-(3-chlorolphenyl)-1,4-dihydropyridine-3,5-dicarboxylate] ^{13}C NMR spectrum	14
**S15**	DHP8: [Diethyl 2,6-dimethyl-4-(3-methoxyphenyl)-1,4-dihydropyridine-3,5-dicarboxylate] ^{1}H NMR spectrum	15
**S16**	DHP8: [Diethyl 2,6-dimethyl-4-(3-methoxyphenyl)-1,4-dihydropyridine-3,5-dicarboxylate] ^{13}C NMR spectrum	16
**S17**	DHP9: [Diethyl 2,6-dimethyl-4-(3-bromophenyl)-1,4-dihydropyridine-3,5-dicarboxylate] ^{1}H NMR spectrum	17
**S18**	DHP9: [Diethyl 2,6-dimethyl-4-(3-bromophenyl)-1,4-dihydropyridine-3,5-dicarboxylate] ^{13}C NMR spectrum	18
**S19**	DHP1: [Diethyl 2,6-dimethyl-4-phenyl-1,4-dihydropyridine-3,5-dicarboxylate] HPLC chromatogram	19

**S20**	DHP2: [Diethyl 2,6-dimethyl-4-(3-methylphenyl)-1,4-dihydropyridine-3,5-dicarboxylate] HPLC chromatogram	20
**S21**	DHP3: [Diethyl 2,6-dimethyl-4-(4-fluorophenyl)-1,4-dihydropyridine-3,5-dicarboxylate] HPLC chromatogram	21
**S22**	DHP4: [Diethyl 2,6-dimethyl-4-(4-chlorophenyl)-1,4-dihydropyridine-3,5-dicarboxylate] HPLC chromatogram	22
**S23**	DHP5: [Diethyl 2,6-dimethyl-4-(3-nitrophenyl)-1,4-dihydropyridine-3,5-dicarboxylate] HPLC chromatogram	23
**S24**	DHP6: [Diethyl 2,6-dimethyl-4-(4-bromophenyl)-1,4-dihydropyridine-3,5-dicarboxylate] HPLC chromatogram	24
**S25**	DHP7: [Diethyl 2,6-dimethyl-4-(3-chlorolphenyl)-1,4-dihydropyridine-3,5-dicarboxylate] HPLC chromatogram	25
**S26**	DHP8: [Diethyl 2,6-dimethyl-4-(3-methoxyphenyl)-1,4-dihydropyridine-3,5-dicarboxylate] HPLC chromatogram	26
**S27**	DHP9: [Diethyl 2,6-dimethyl-4-(3-bromophenyl)-1,4-dihydropyridine-3,5-dicarboxylate] HPLC chromatogram	27
**S28**	Molecular Docking Analysis	28
**S29**	Validation of the molecular docking protocol by redocking of the co-crystallized inhibitor minalrestat	29

**Table**	Caption	Page
**S1**	Physicochemical Properties of Dihydropyridines	30
**S2**	Lipophilicity of Dihydropyridines	30
**S3**	Water Solubility of Dihydropyridines	31
**S4**	Pharmacokinetics of Dihydropyridines	31
**S5**	Druglikeness and Medicinal Chemistry of Dihydropyridines	32

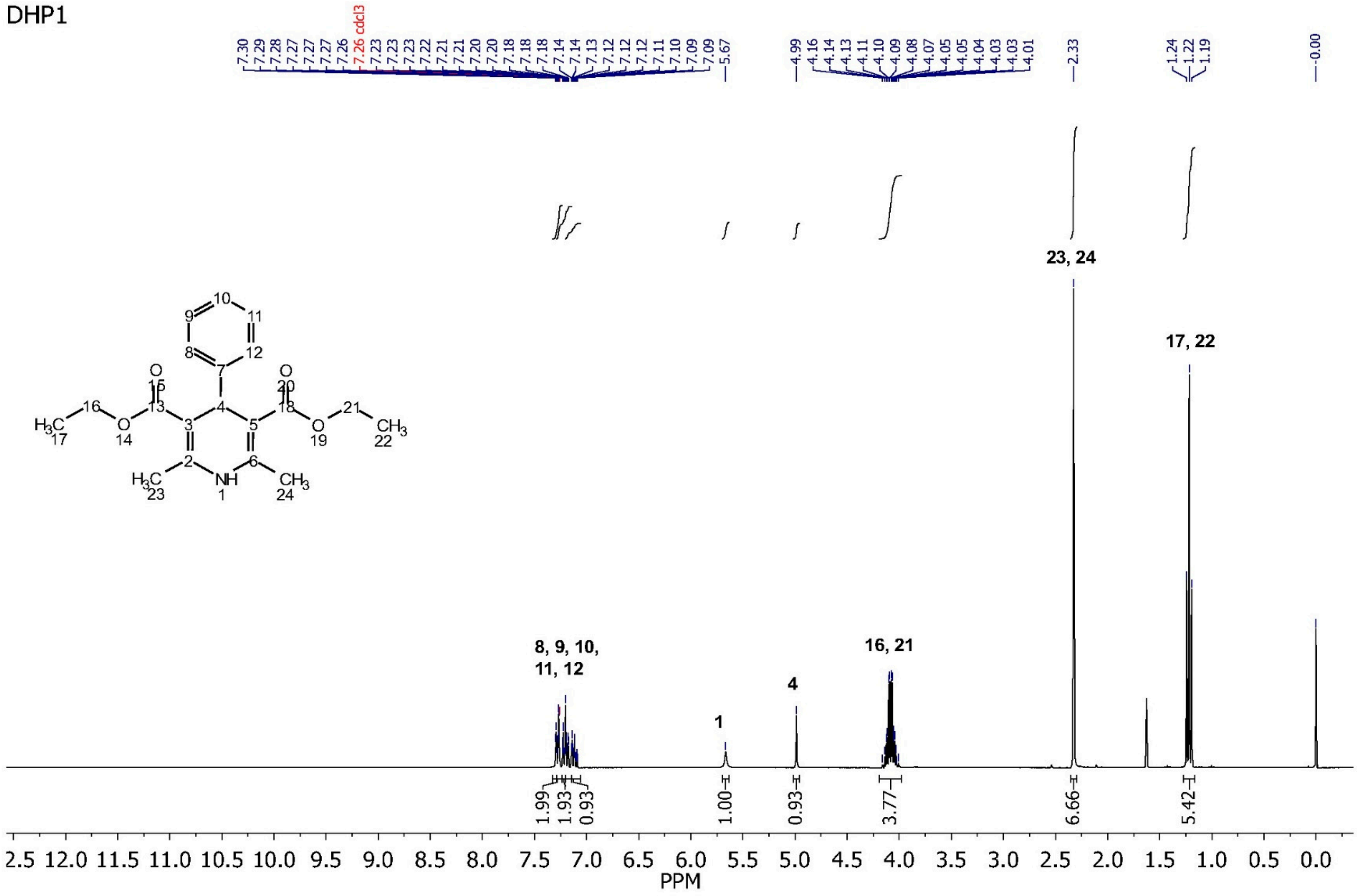

Figure S1. ^{1}H NMR spectrum of DHP1 [Diethyl 2,6-dimethyl-4-phenyl-1,4 dihydropyridine-3,5-dicarboxylate] compound (300 MHz, $CDCl_3$).

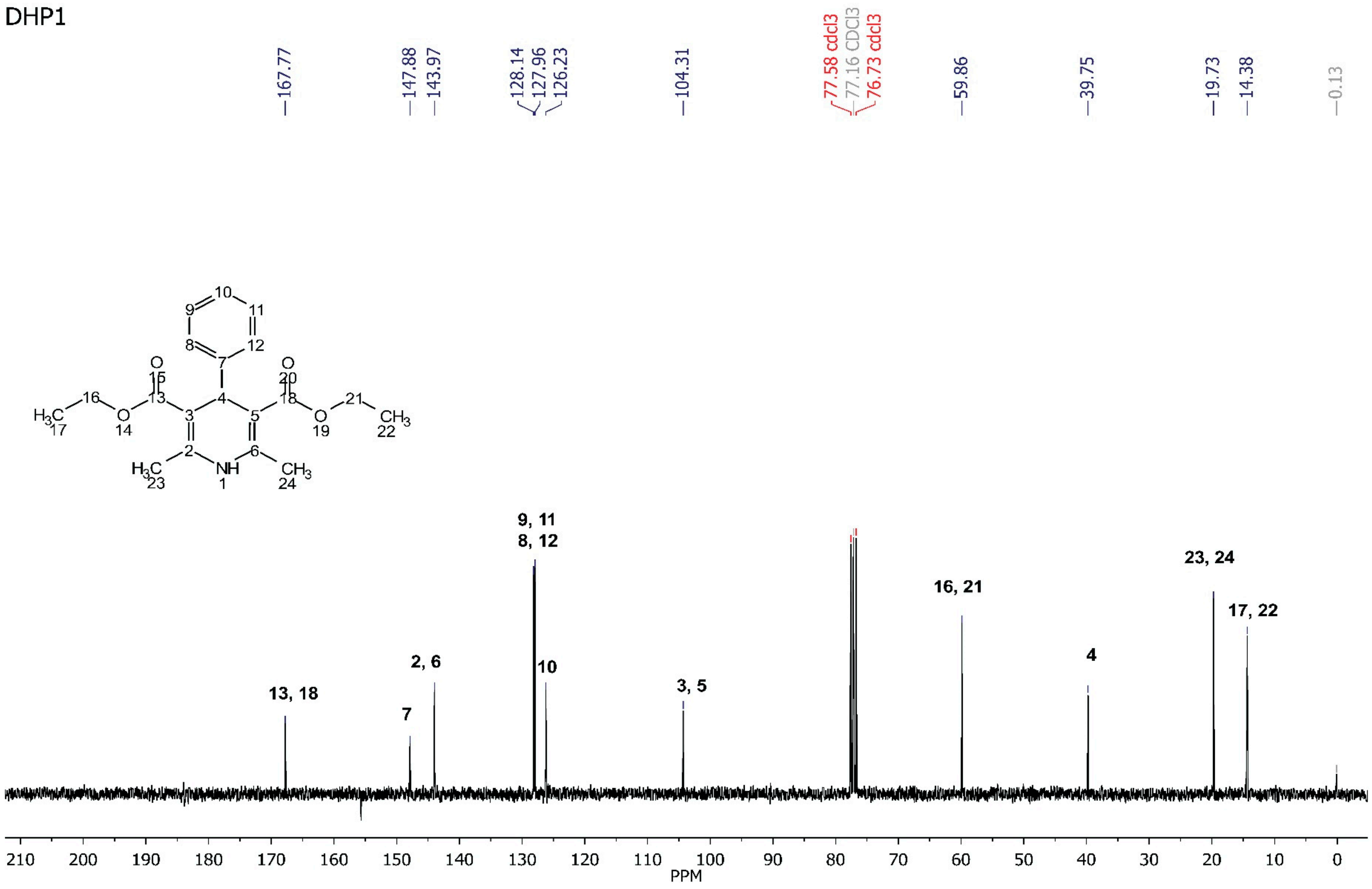

Figure S2. ^{13}C NMR spectrum of DHP1 [Diethyl 2,6-dimethyl-4-phenyl-1,4-dihydropyridine-3,5-dicarboxylate] compound (75 MHz, $CDCl_3$).

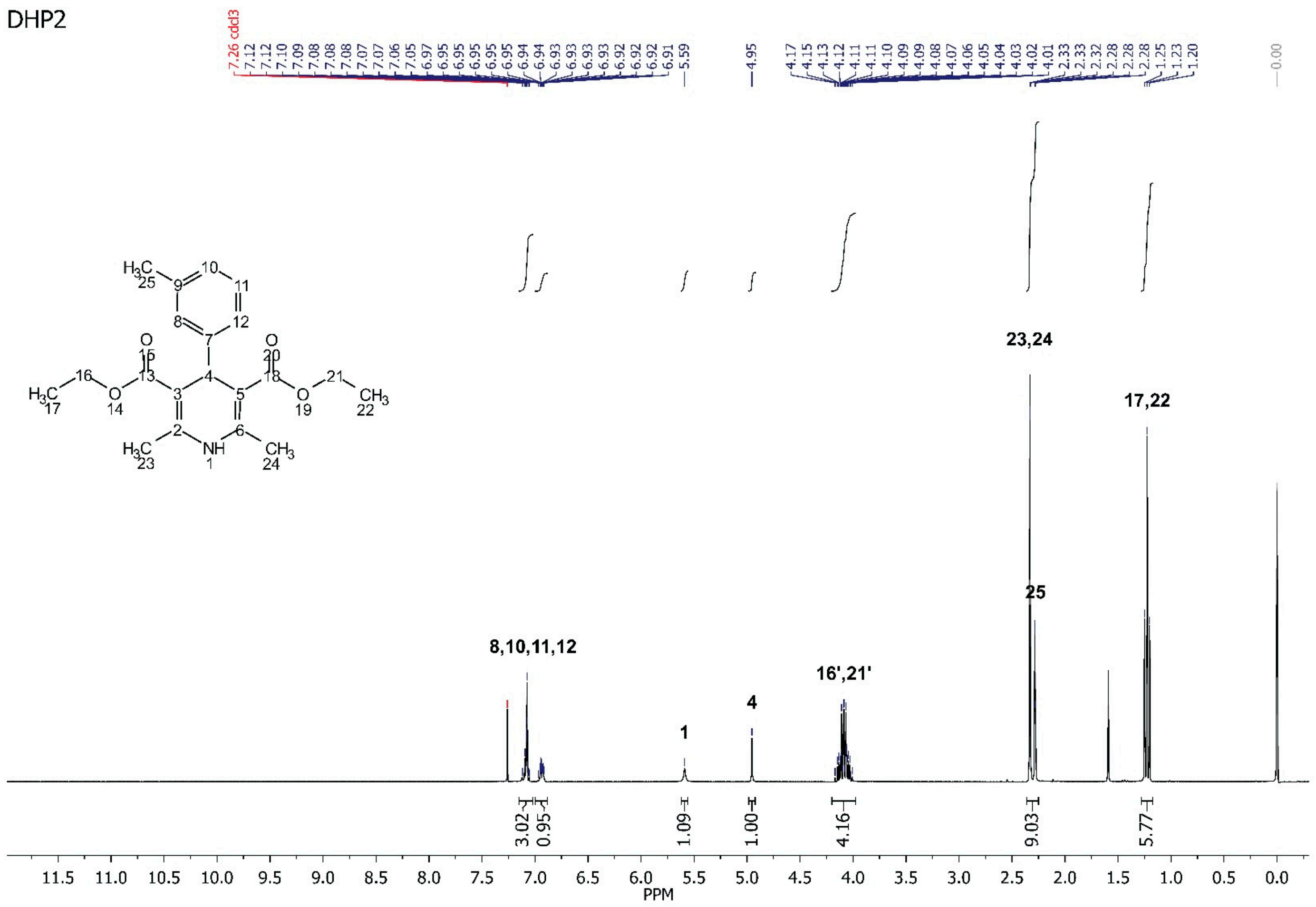

Figure S3. ^{1}H NMR spectrum of DHP2 [Diethyl 2,6-dimethyl-4-phenyl-1,4-dihydropyridine-3,5-dicarboxylate] compound (300 MHz, $CDCl_3$)

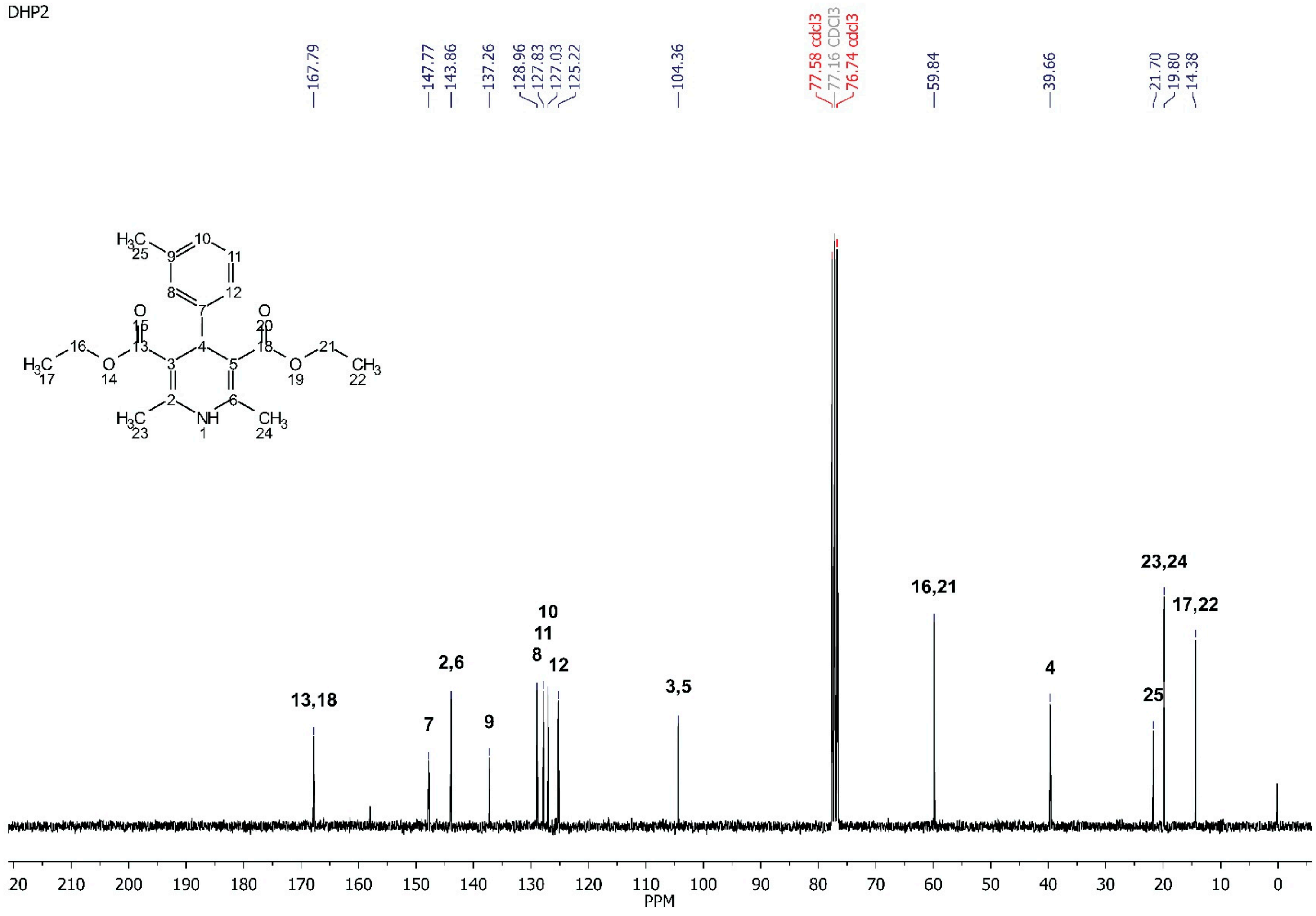

Figure S4. ^{13}C NMR spectrum of DHP2 [Diethyl 2,6-dimethyl-4-phenyl-1,4-dihydropyridine-3,5-dicarboxylate] compound (75 MHz, $CDCl_3$).

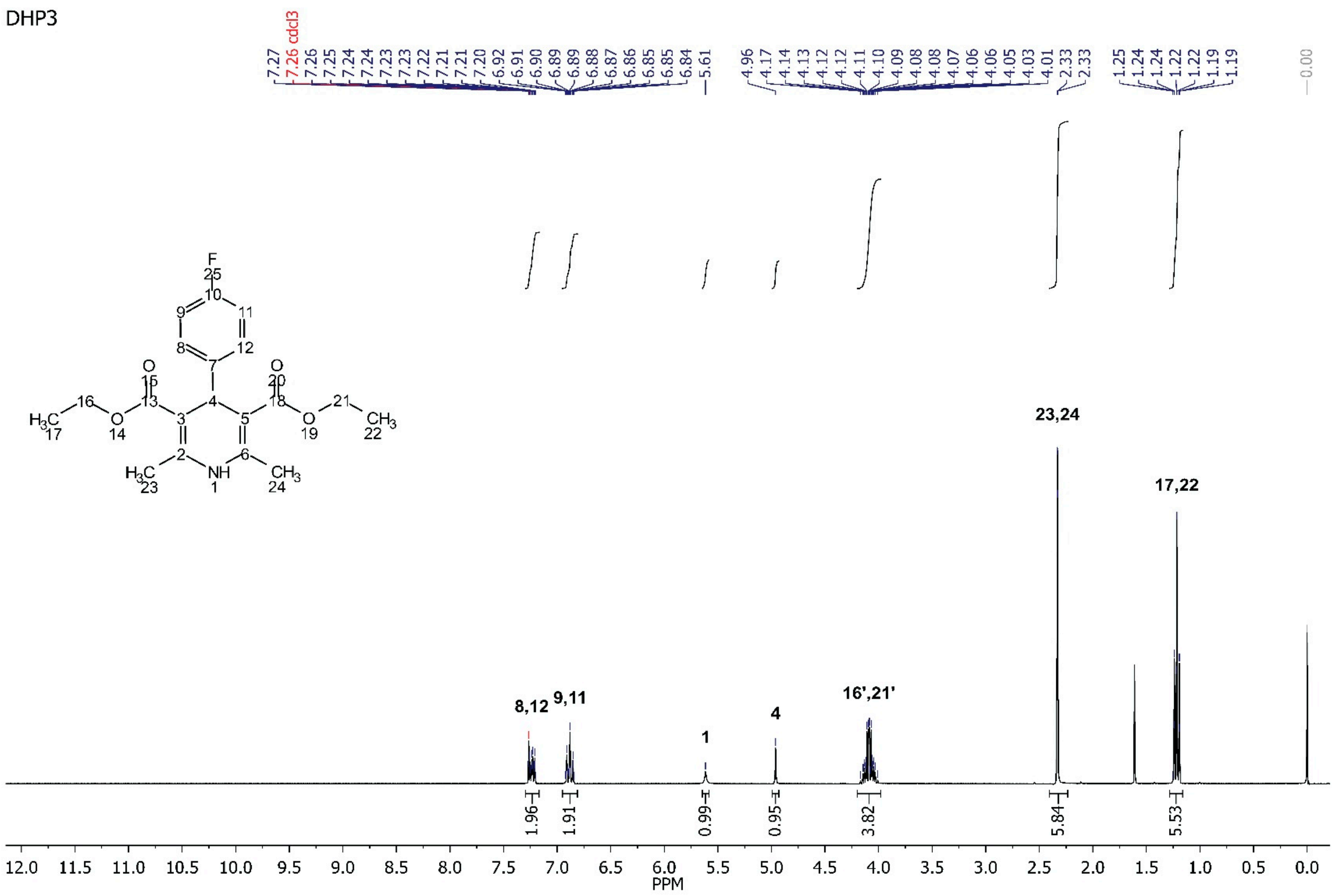

Figure S5. ^{1}H NMR spectrum of DHP3 [Diethyl 2,6-dimethyl-4-(4-fluorophenyl)-1,4-dihydropyridine-3,5-dicarboxylate] compound (300 MHz, $CDCl_3$)

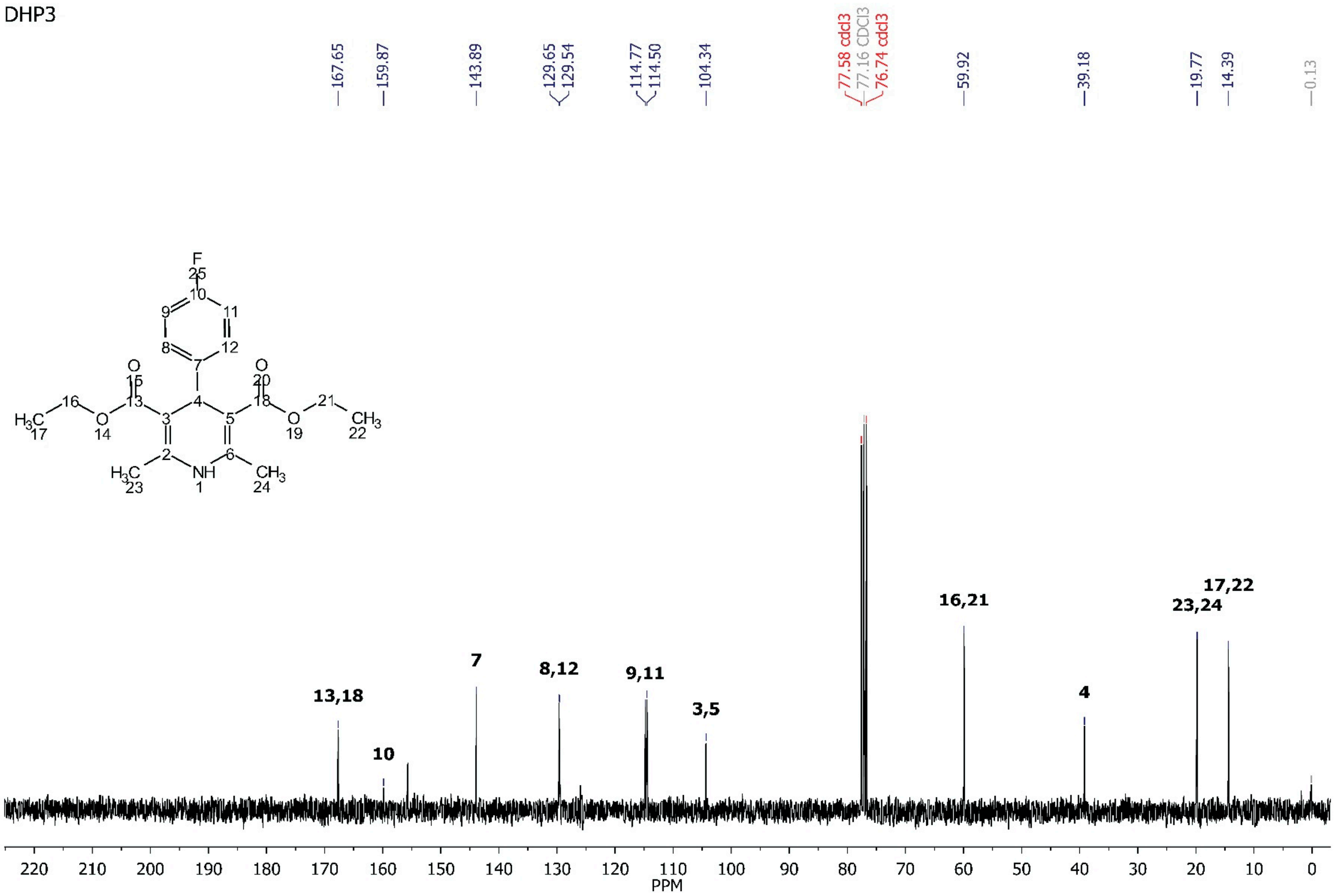

Figure S6. ^{13}C NMR spectrum of DHP3 [Diethyl 2,6-dimethyl-4-(4-fluorophenyl)-1,4-dihydropyridine-3,5-dicarboxylate] compound (75 MHz, $CDCl_3$).

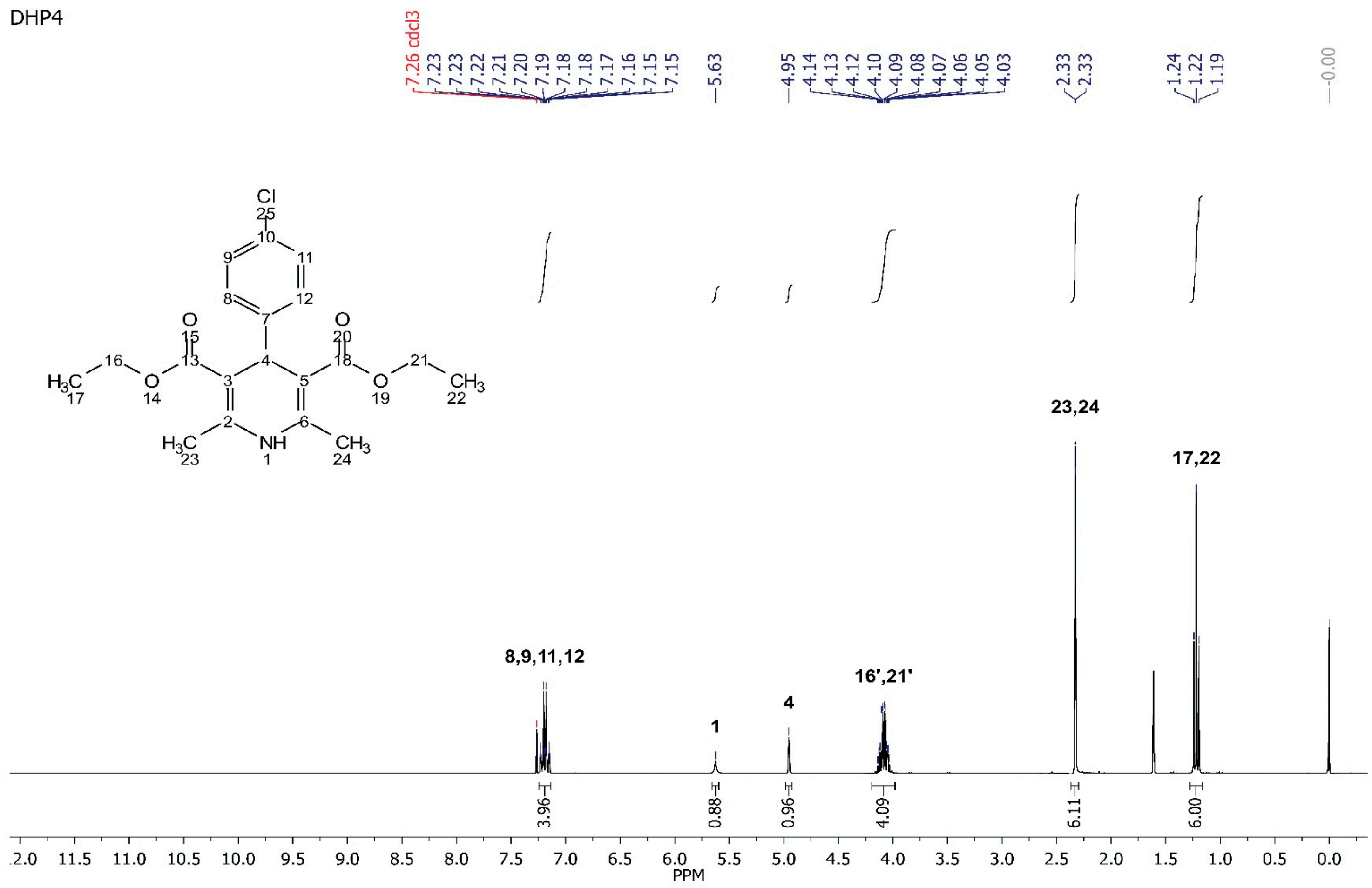

Figure S7. ^{1}H NMR spectrum of DHP4 [Diethyl 2,6-dimethyl-4-(4-chlorophenyl)-1,4-dihydropyridine-3,5-dicarboxylate] compound (300 MHz, $CDCl_3$)

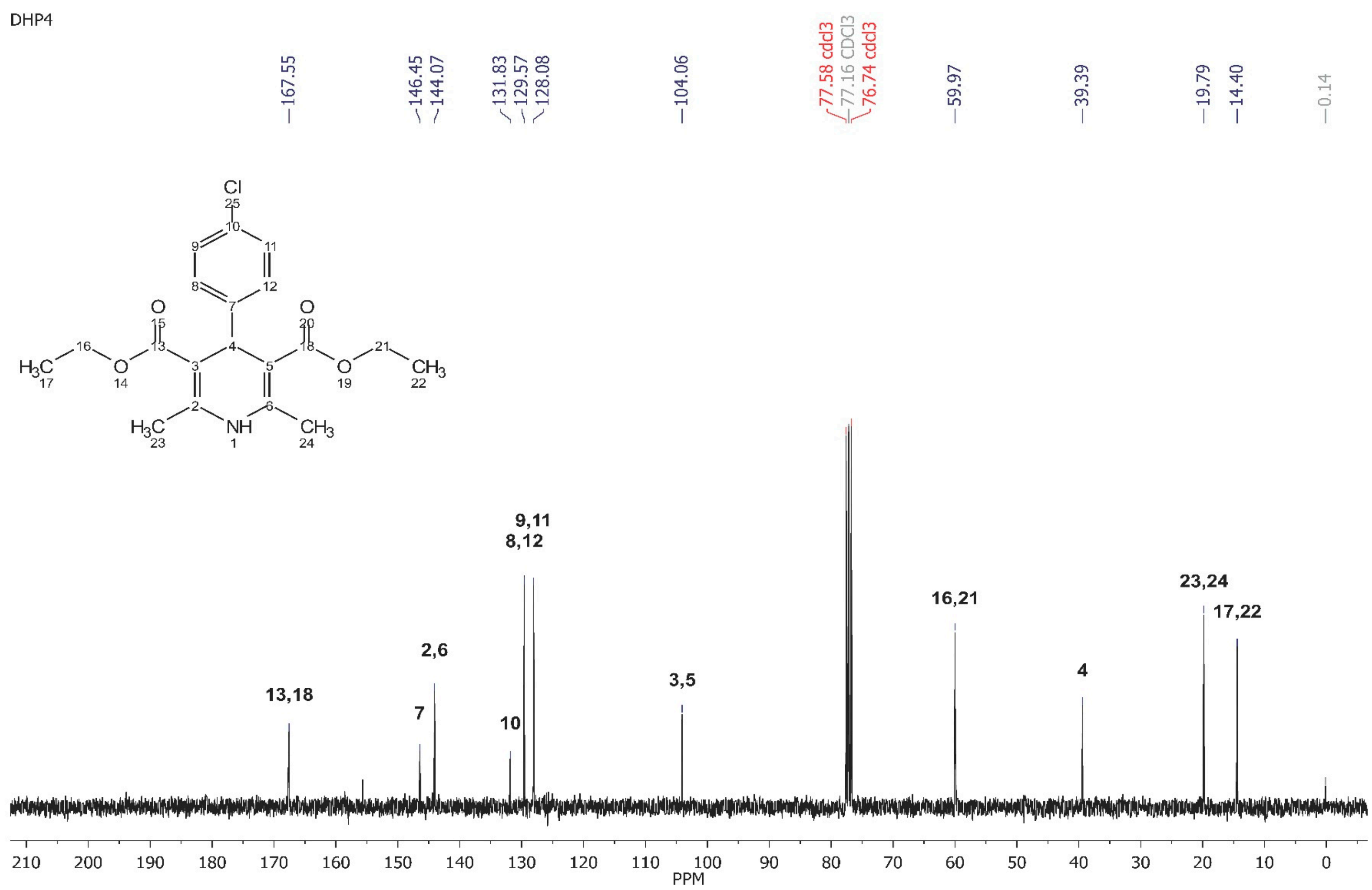

Figure S8. ^{13}C NMR spectrum of DHP4 [Diethyl 2,6-dimethyl-4-(4-chlorophenyl)-1,4-dihydropyridine-3,5-dicarboxylate] compound (75 MHz, $CDCl_3$).

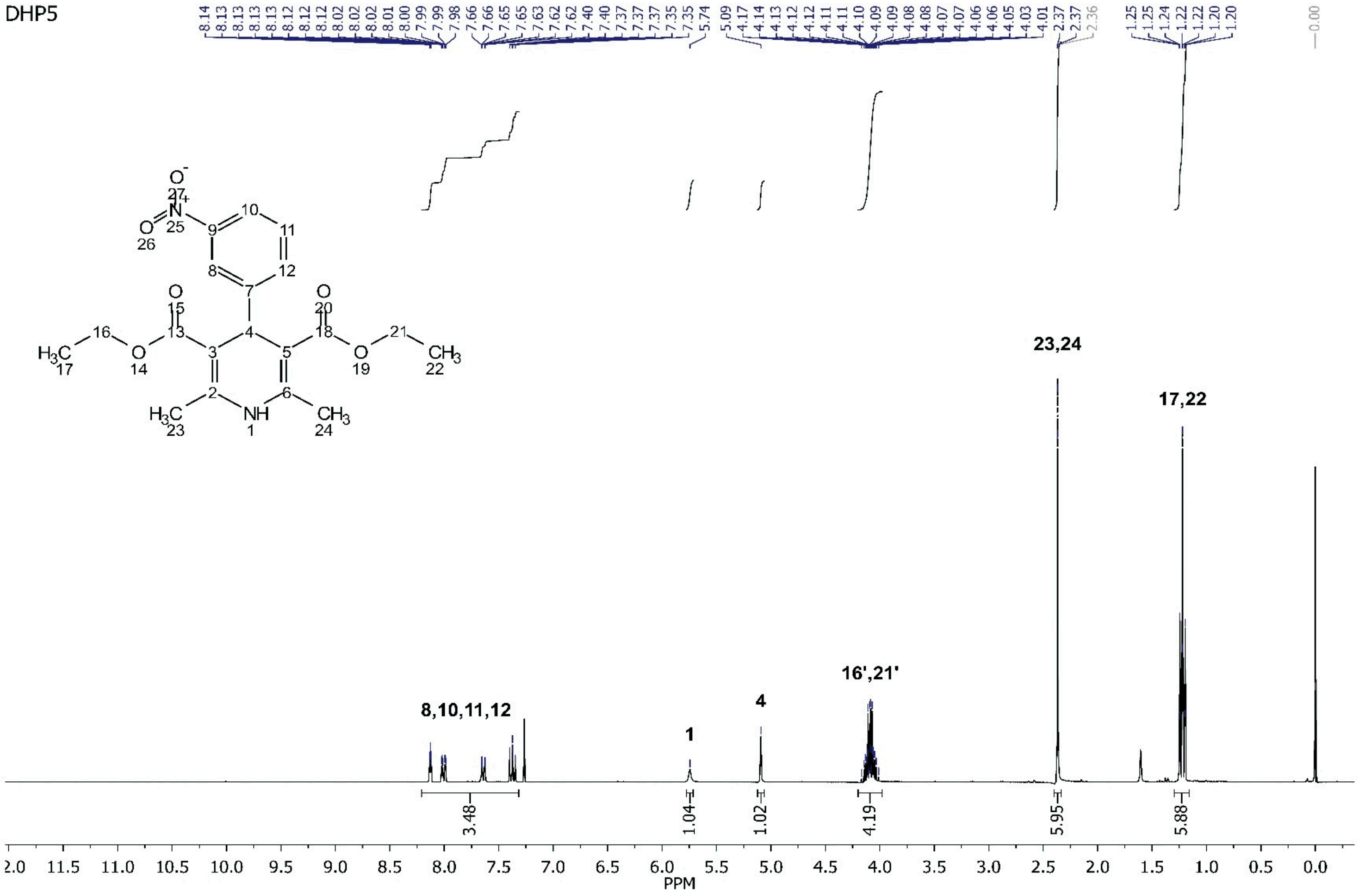

Figure S9. ^{1}H NMR spectrum of DHP5 [Diethyl 2,6-dimethyl-4-(3-nitrophenyl)-1,4-dihydropyridine-3,5-dicarboxylate] compound (300 MHz, $CDCl_3$).

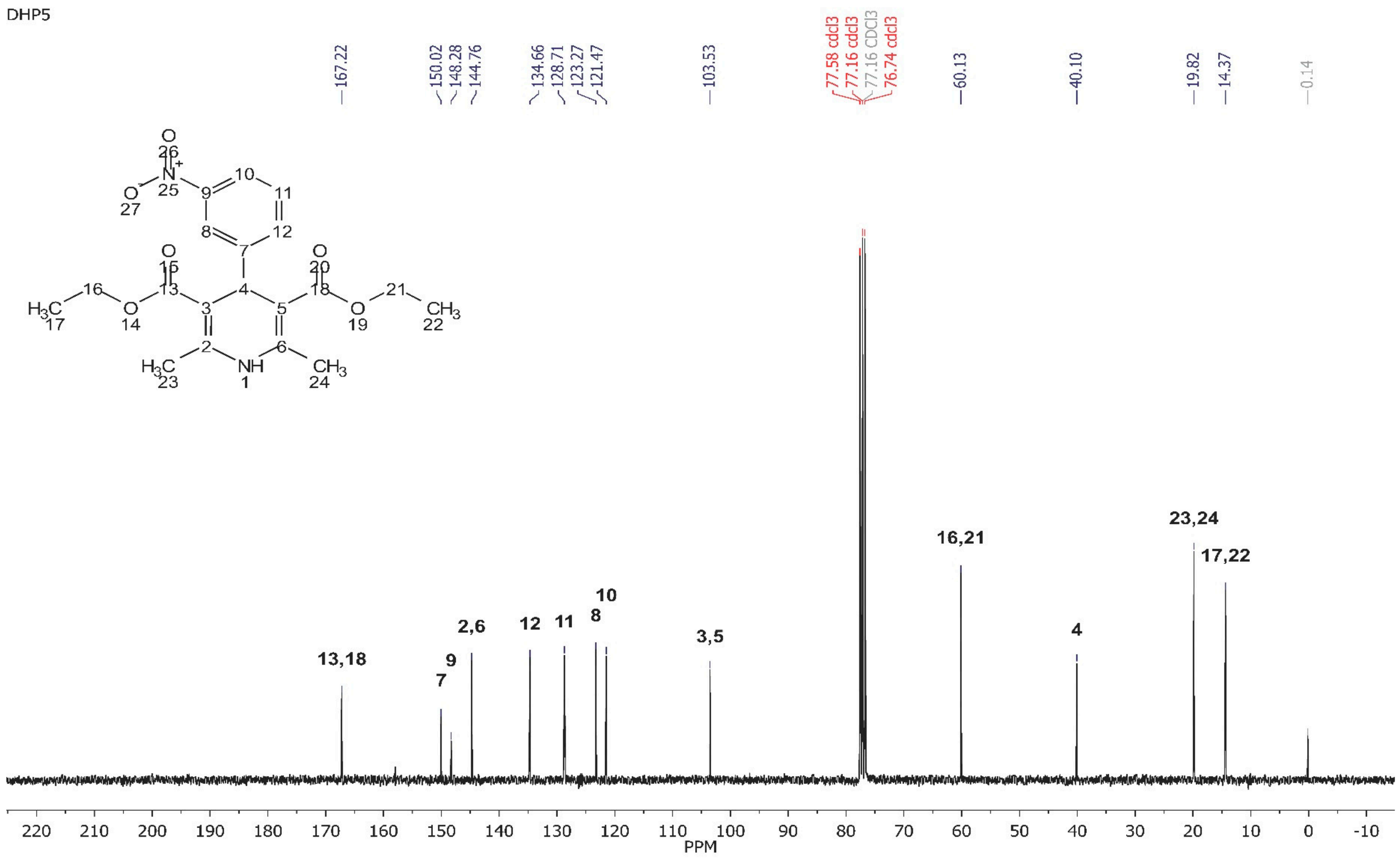

Figure S10. ^{13}C NMR spectrum of DHP5 [Diethyl 2,6-dimethyl-4-(3-nitrophenyl)-1,4-dihydropyridine-3,5-dicarboxylate] compound (75 MHz, $CDCl_3$).

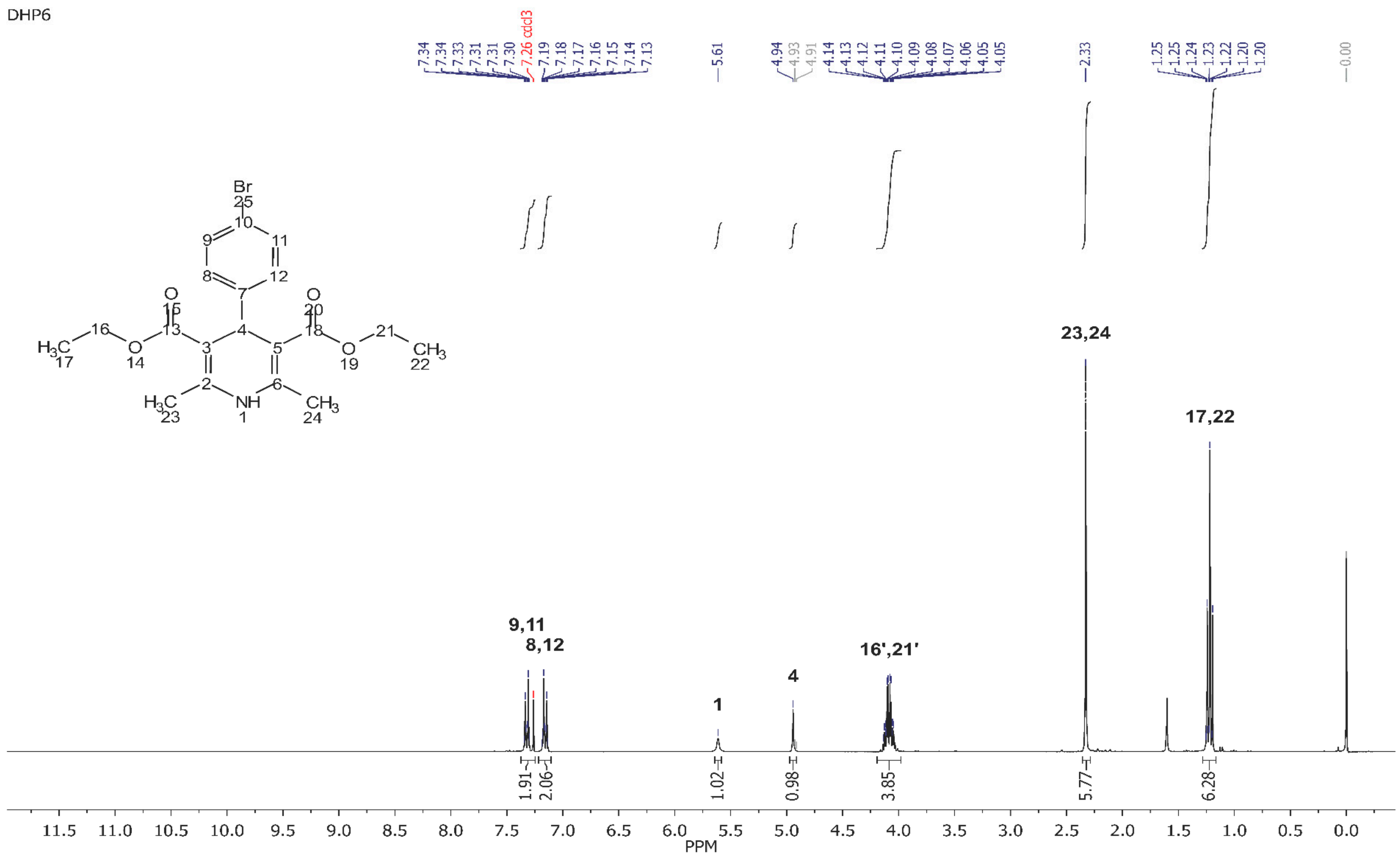

Figure S11. ^{1}H NMR spectrum of DHP6 [Diethyl 2,6-dimethyl-4-(4-bromophenyl)-1,4-dihydropyridine-3,5-dicarboxylate] compound (300 MHz, $CDCl_3$).

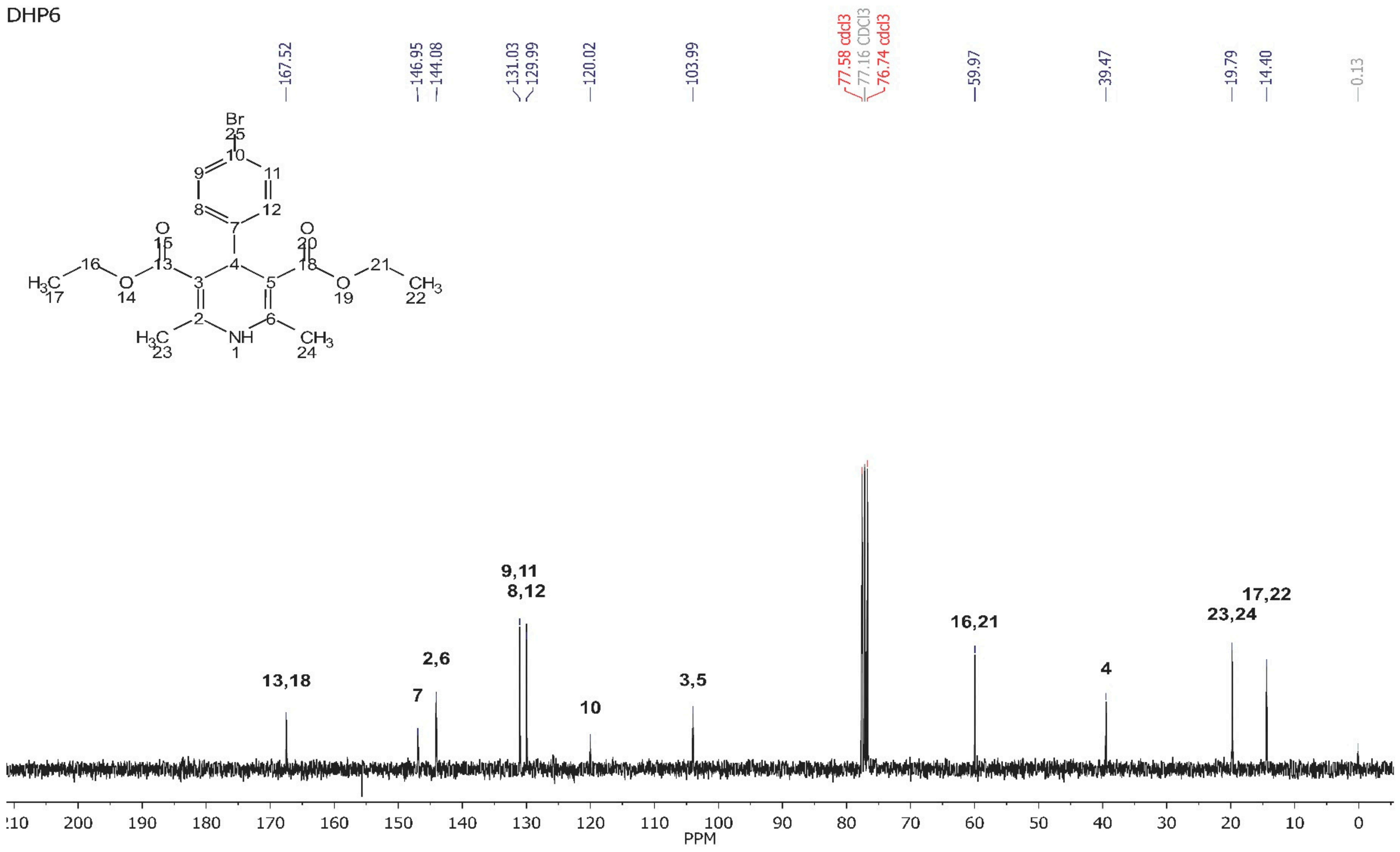

Figure S12. ^{13}C NMR spectrum of DHP6 [Diethyl 2,6-dimethyl-4-(4-bromophenyl)-1,4-dihydropyridine-3,5-dicarboxylate] compound (75 MHz, $CDCl_3$).

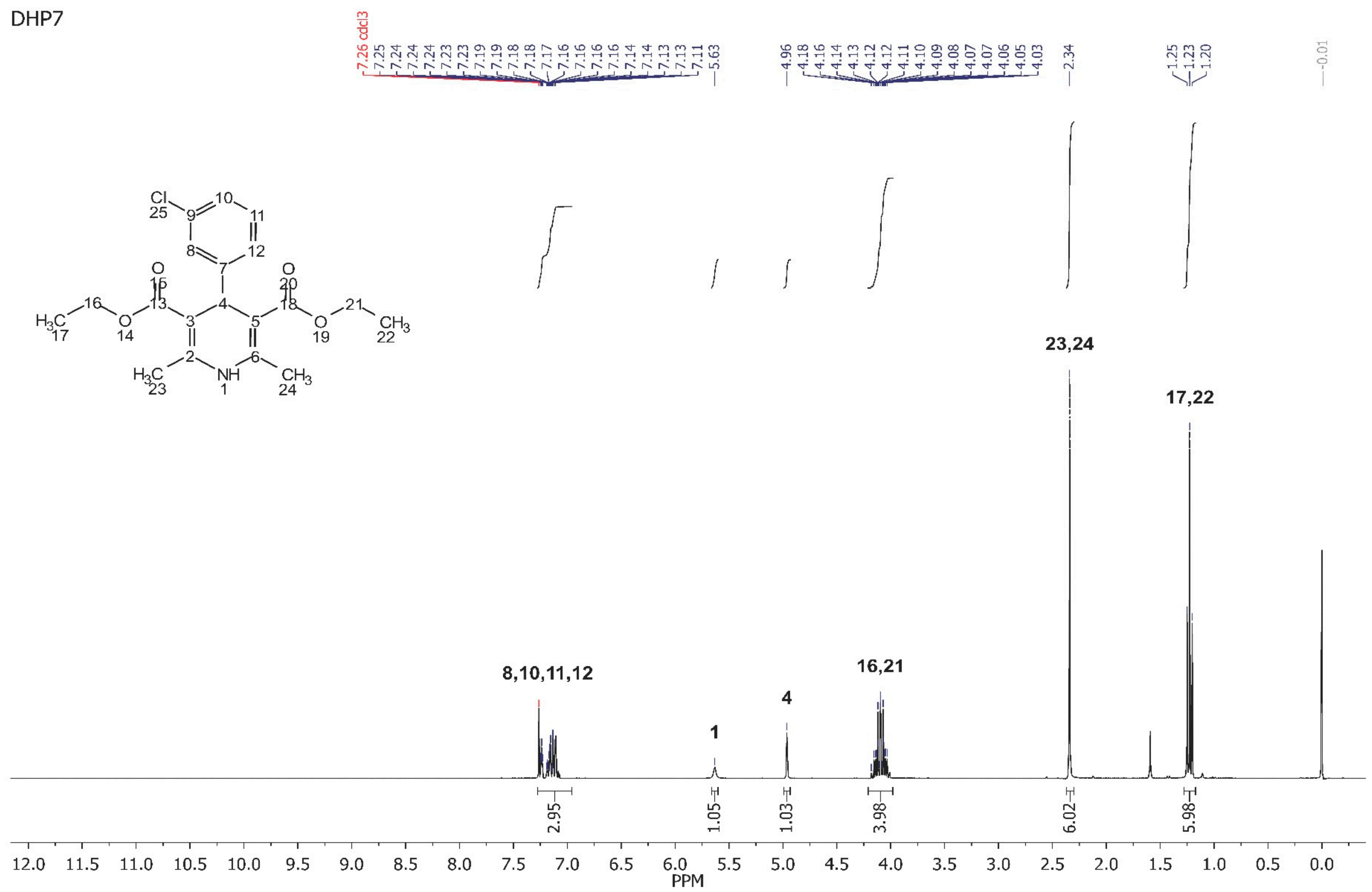

Figure S13. ^{1}H NMR spectrum of DHP7 [Diethyl 2,6-dimethyl-4-(3-chlorolphenyl)-1,4-dihydropyridine-3,5-dicarboxylate] compound (300 MHz, $CDCl_3$).

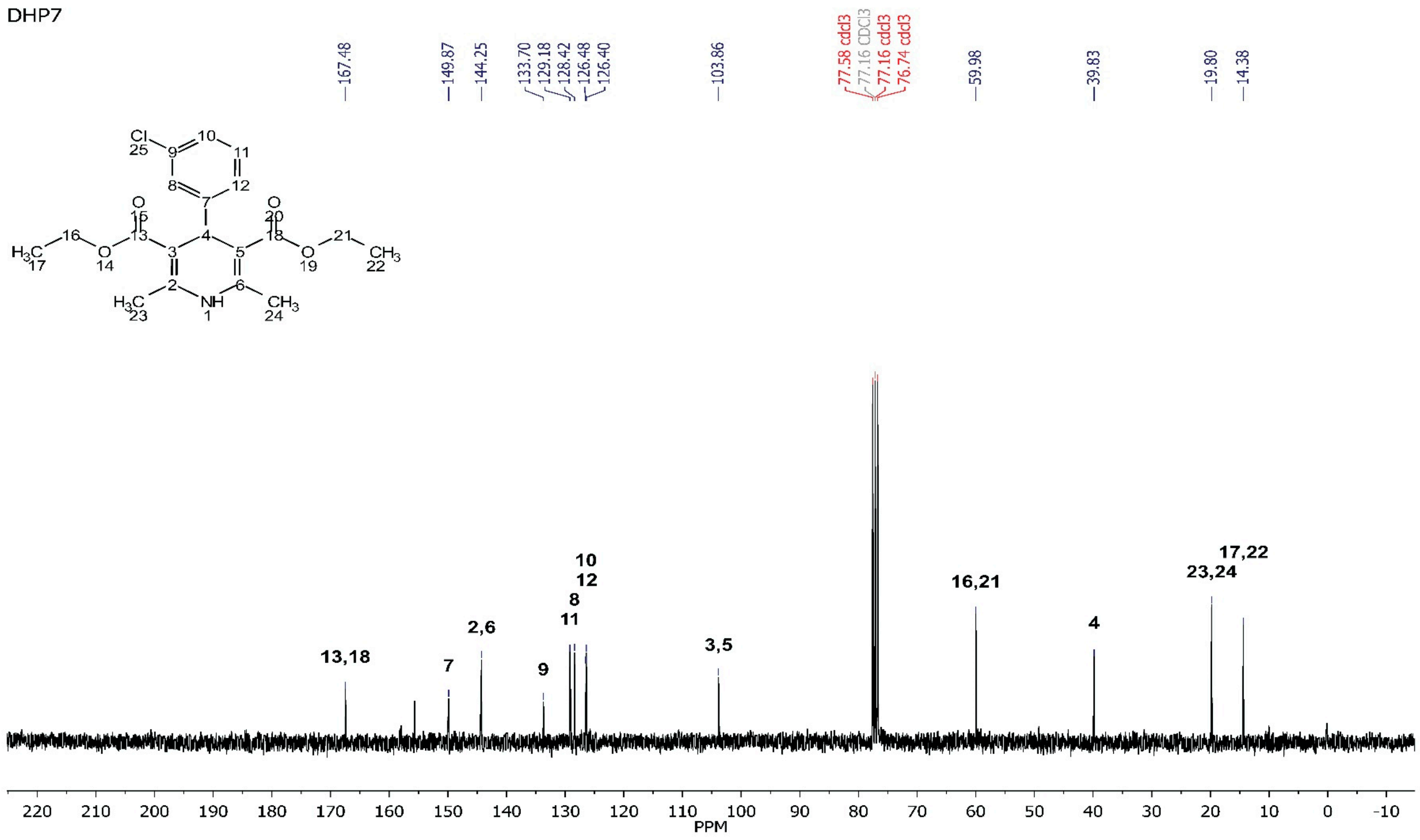

Figure S14. ^{13}C NMR spectrum of DHP7 [Diethyl 2,6-dimethyl-4-(3-chlorolphenyl)-1,4-dihydropyridine-3,5-dicarboxylate] compound (75 MHz, $CDCl_3$).

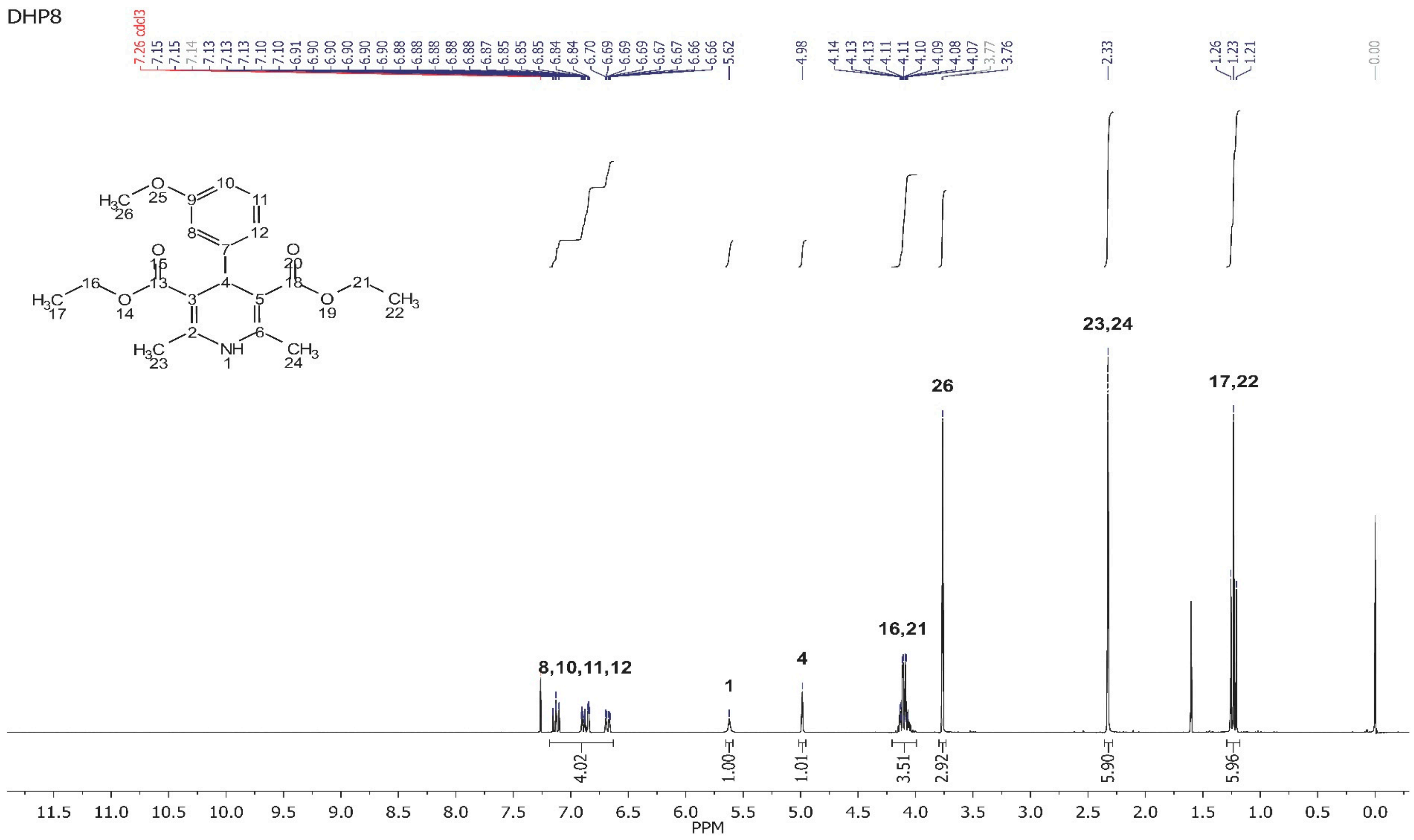

Figure S15. ^{1}H NMR spectrum of DHP8 [Diethyl 2,6-dimethyl-4-(3-methoxyphenyl)-1,4-dihydropyridine-3,5-dicarboxylate] compound (300 MHz, $CDCl_3$).

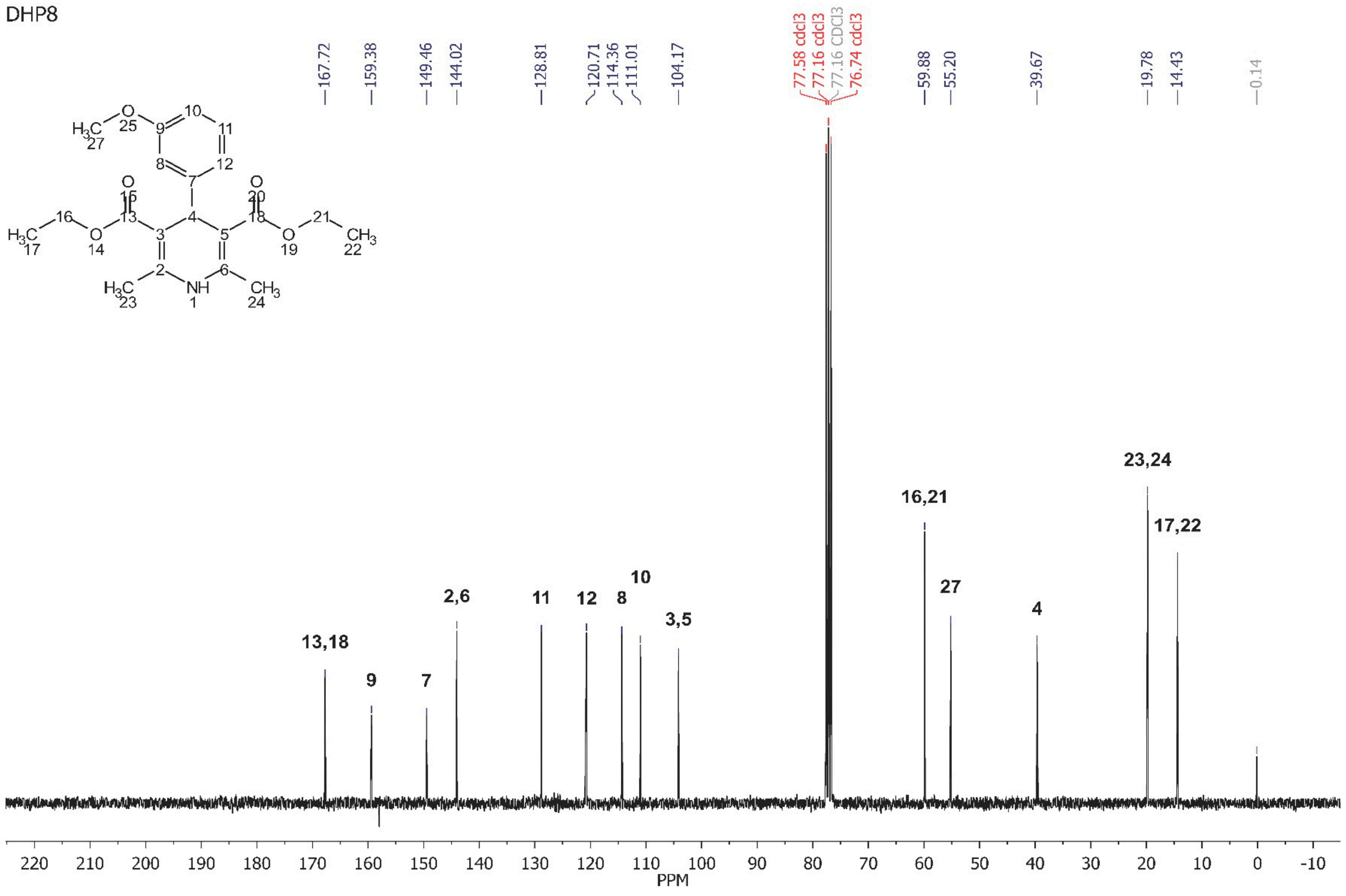

Figure S16. ^{13}C NMR spectrum of DHP8 [Diethyl 2,6-dimethyl-4-(3-methoxyphenyl)-1,4-dihydropyridine-3,5-dicarboxylate] compound (75 MHz, $CDCl_3$).

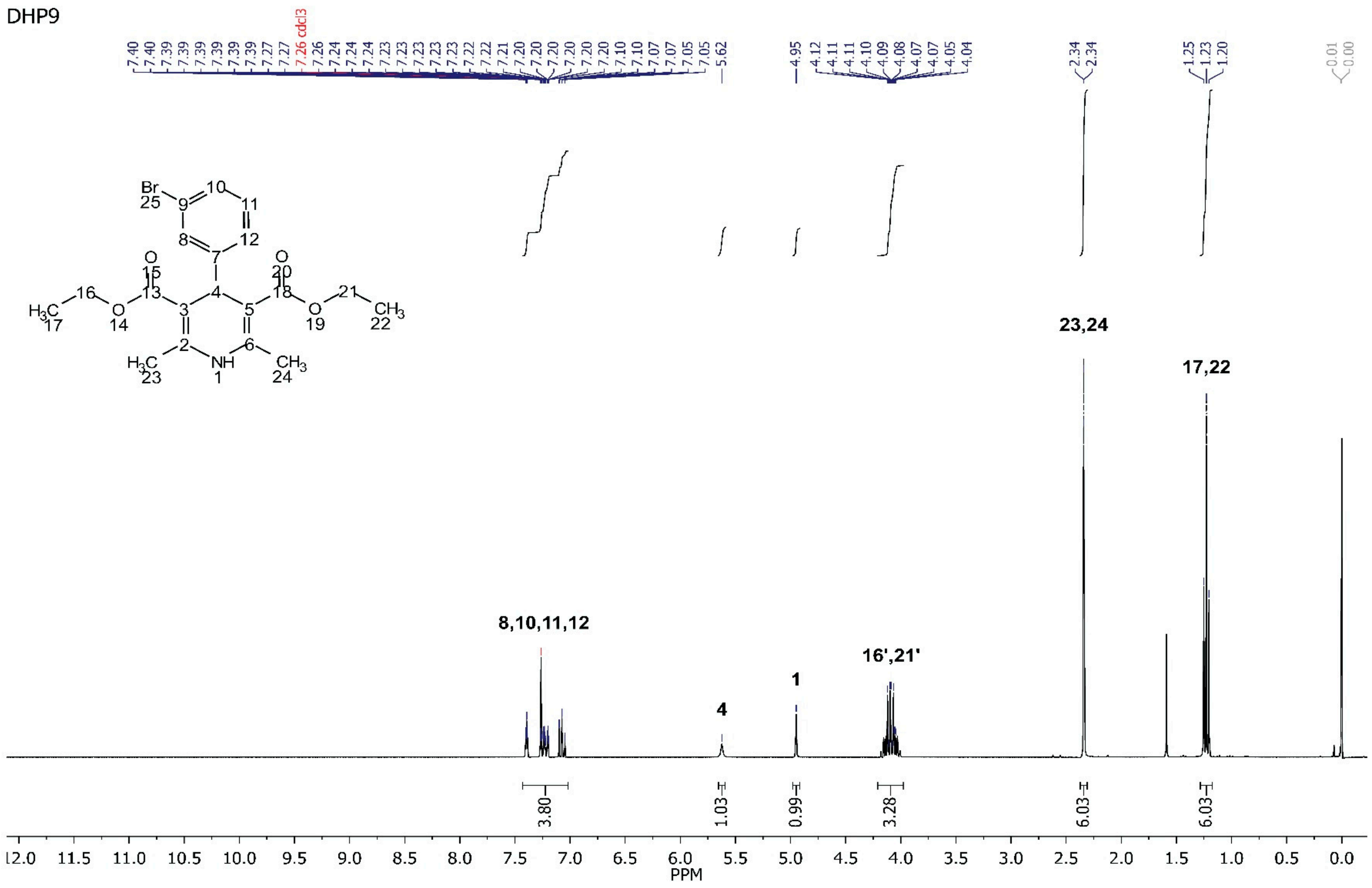

Figure S17. ^{1}H NMR spectrum of DHP9 [Diethyl 2,6-dimethyl-4-(3-bromophenyl)-1,4-dihydropyridine-3,5-dicarboxylate] compound (300 MHz, $CDCl_3$).

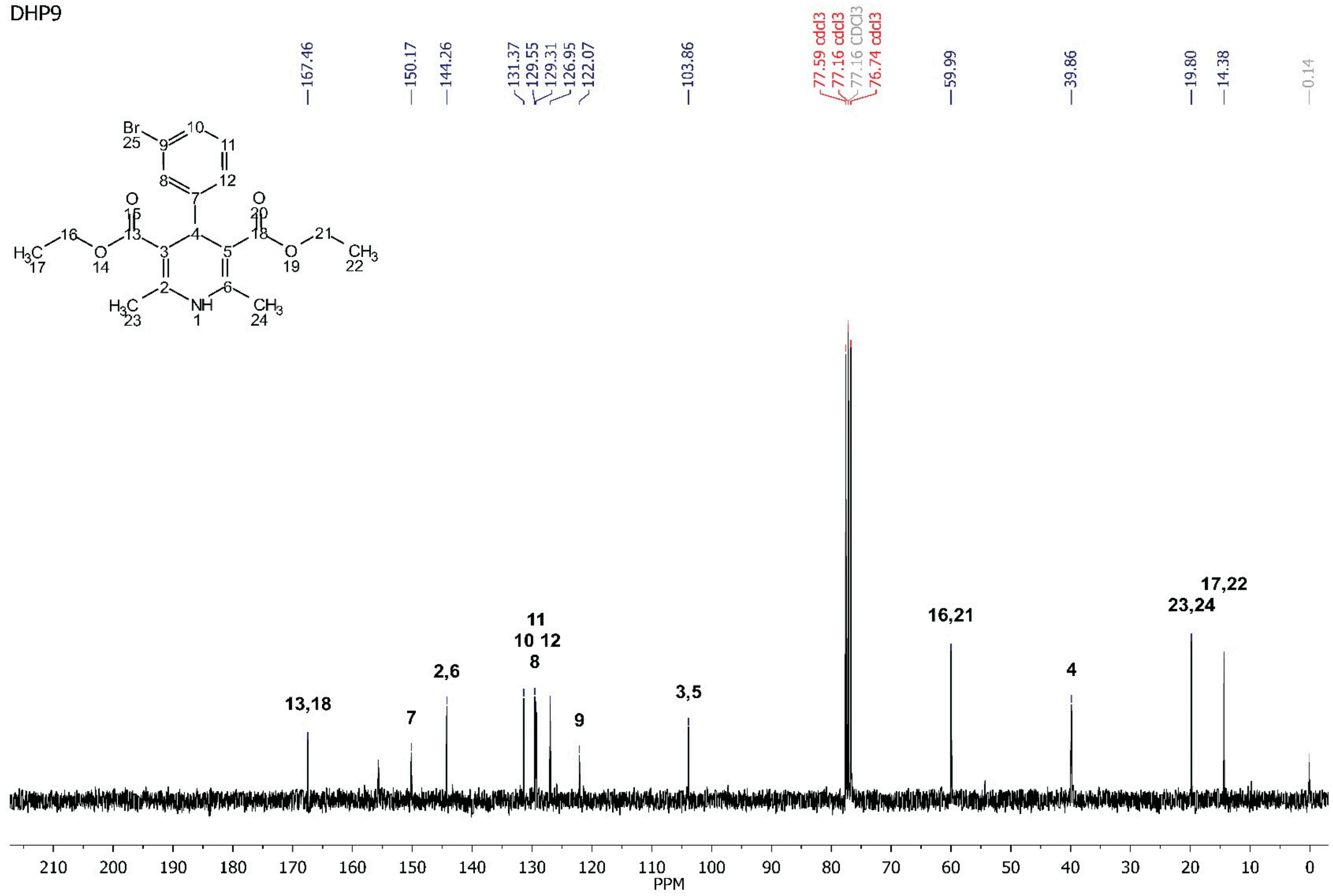

Figure S18. ^{13}C NMR spectrum of DHP9 [Diethyl 2,6-dimethyl-4-(3-bromophenyl)-1,4-dihydropyridine-3,5-dicarboxylate] compound (75 MHz, $CDCl_3$).

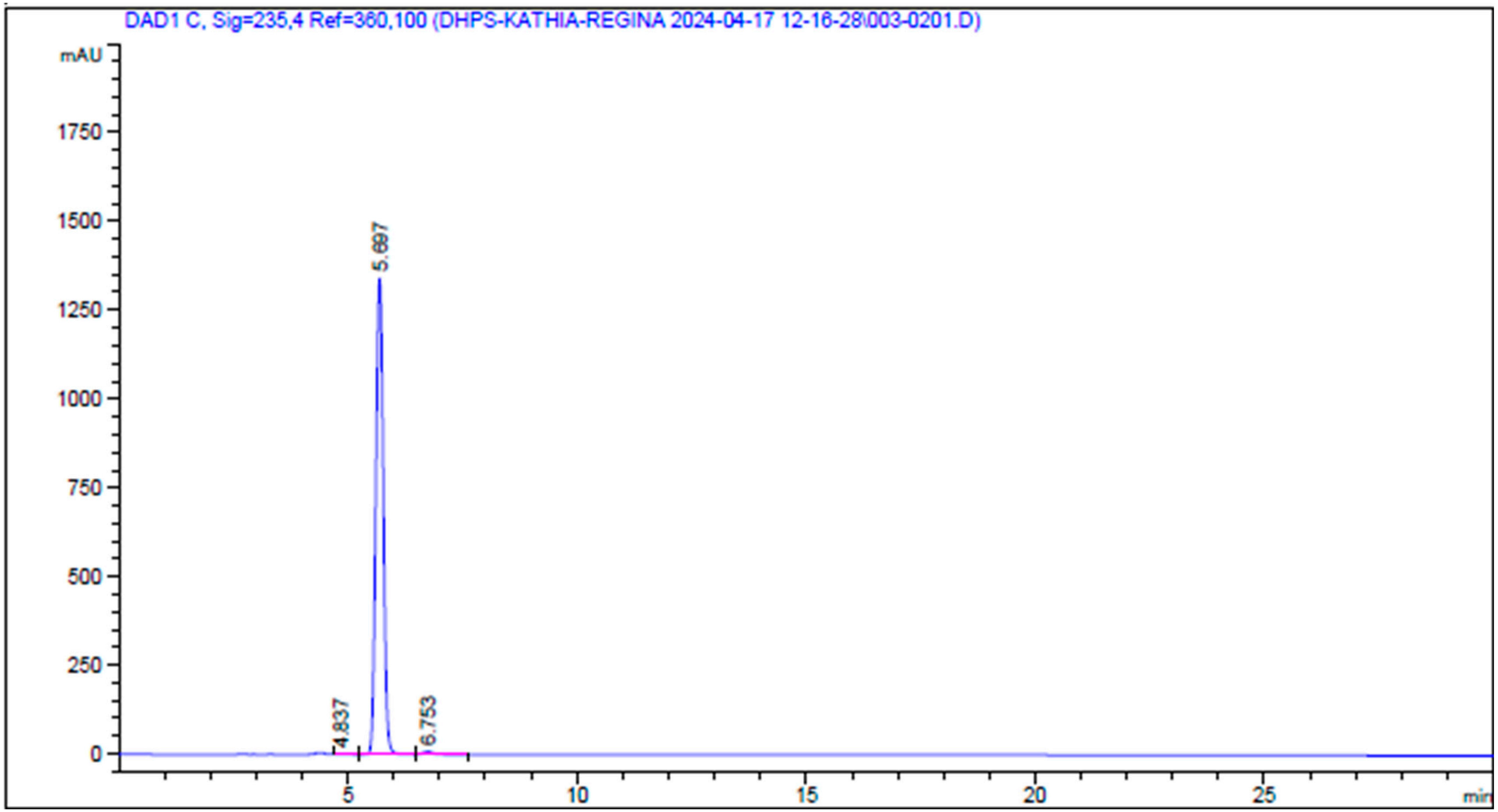

Figure S19. HPLC chromatogram of DHP1 compound (purity: 99.1%).

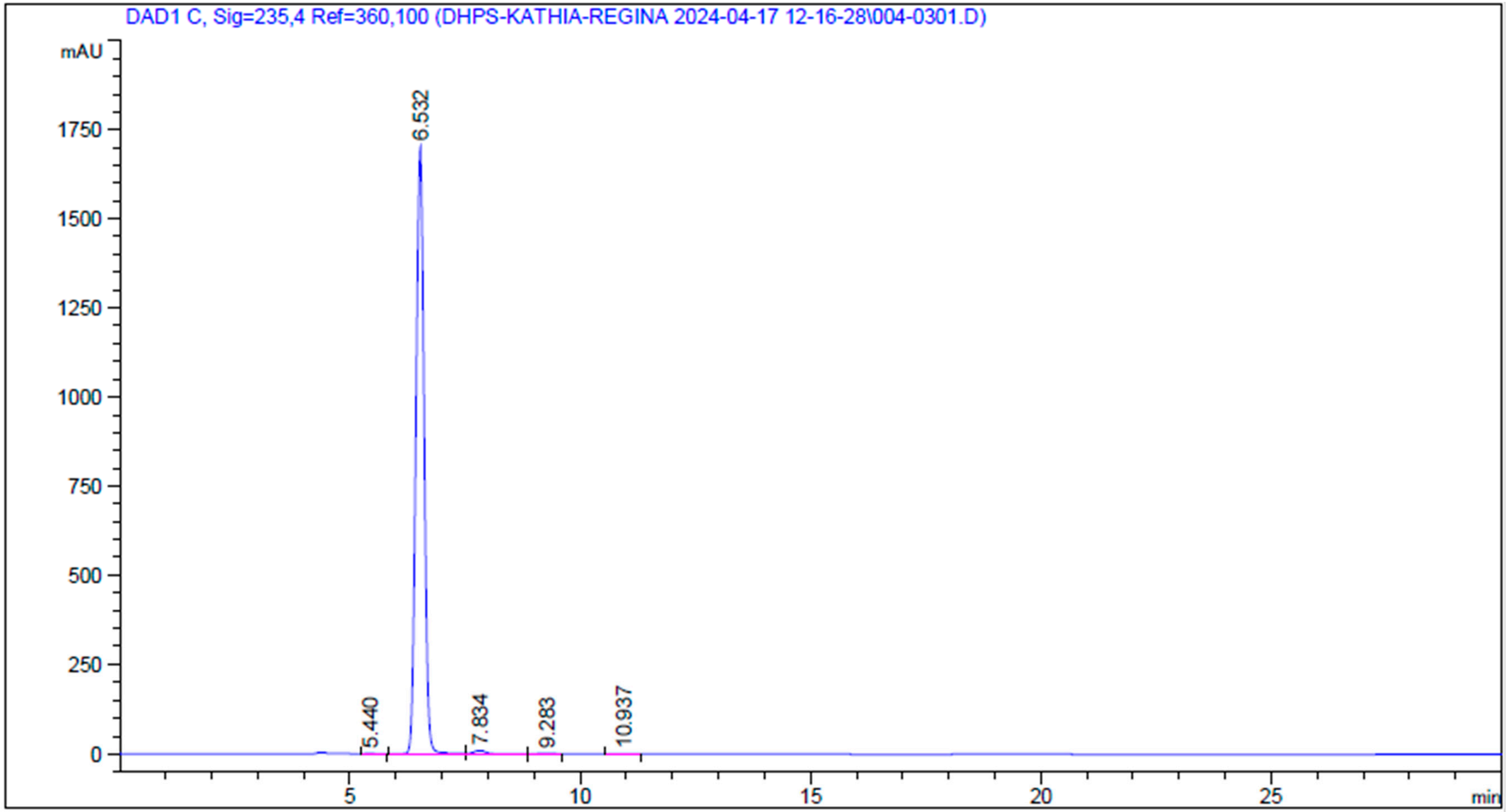

Figure S20. HPLC chromatogram of DHP2 compound (purity: 99.1%).

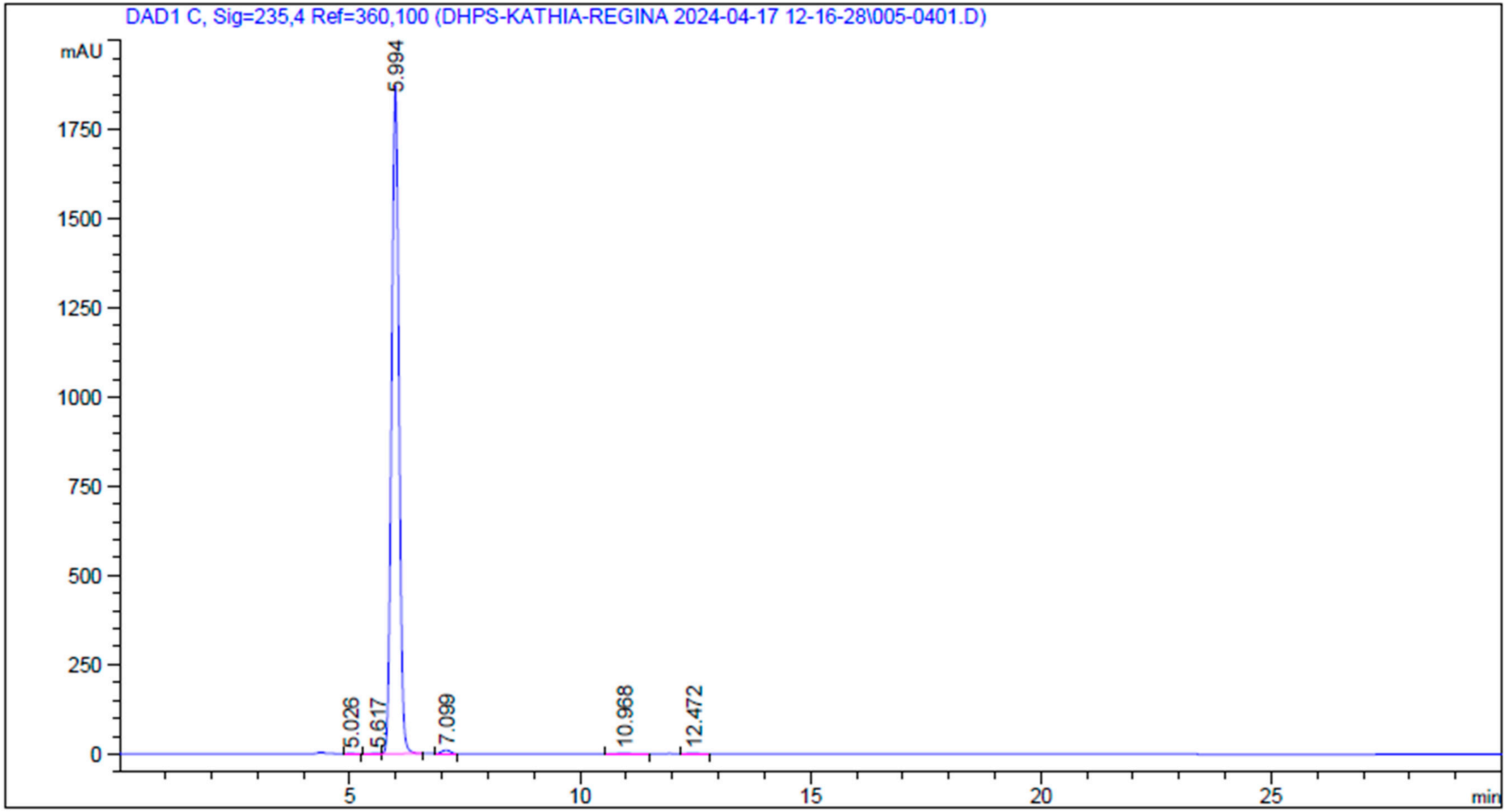

Figure S21. HPLC chromatogram of DHP3 compound (purity: 99.1%).

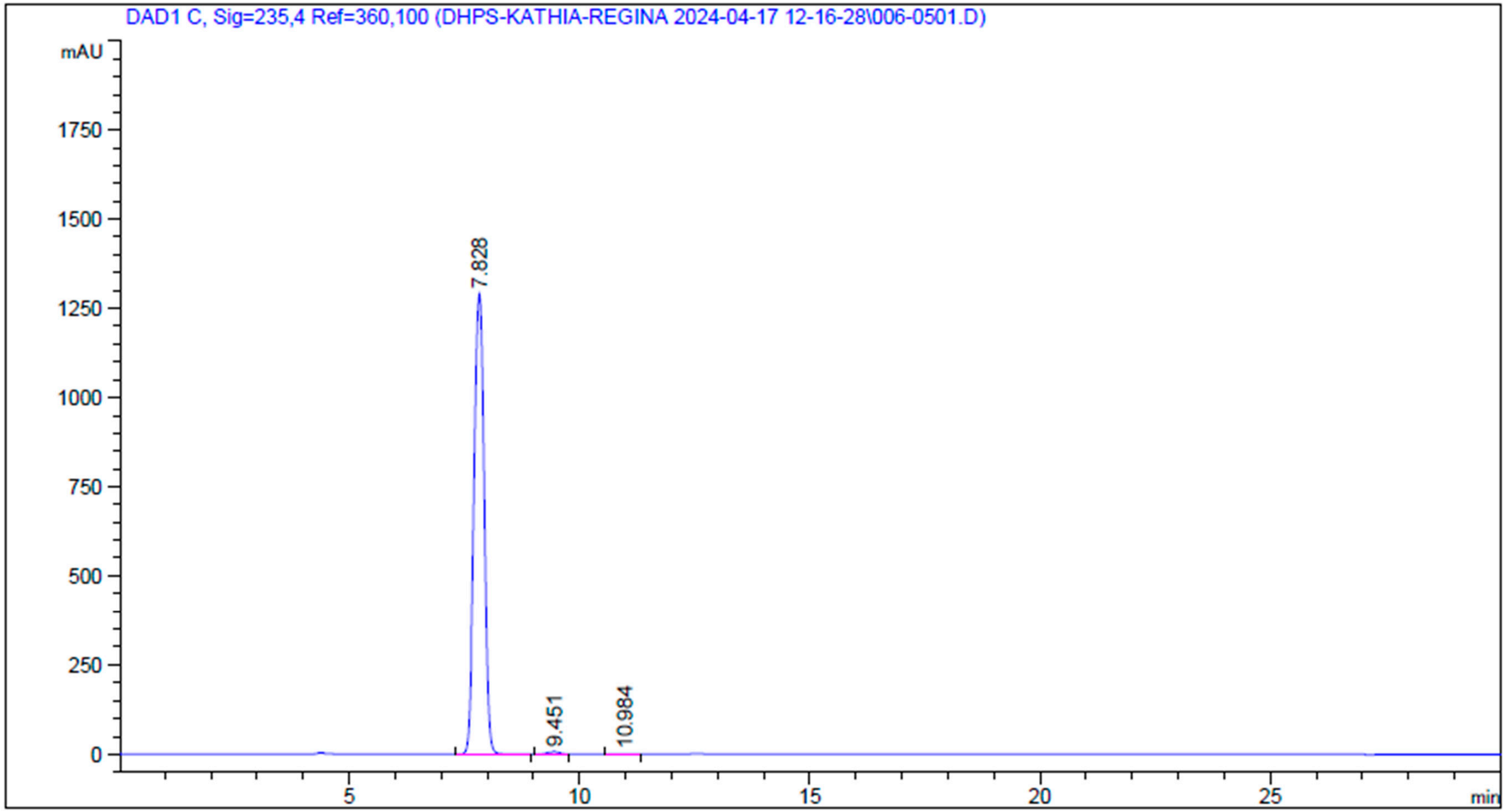

Figure S22. HPLC chromatogram of DHP4 compound (purity: 99.1%).

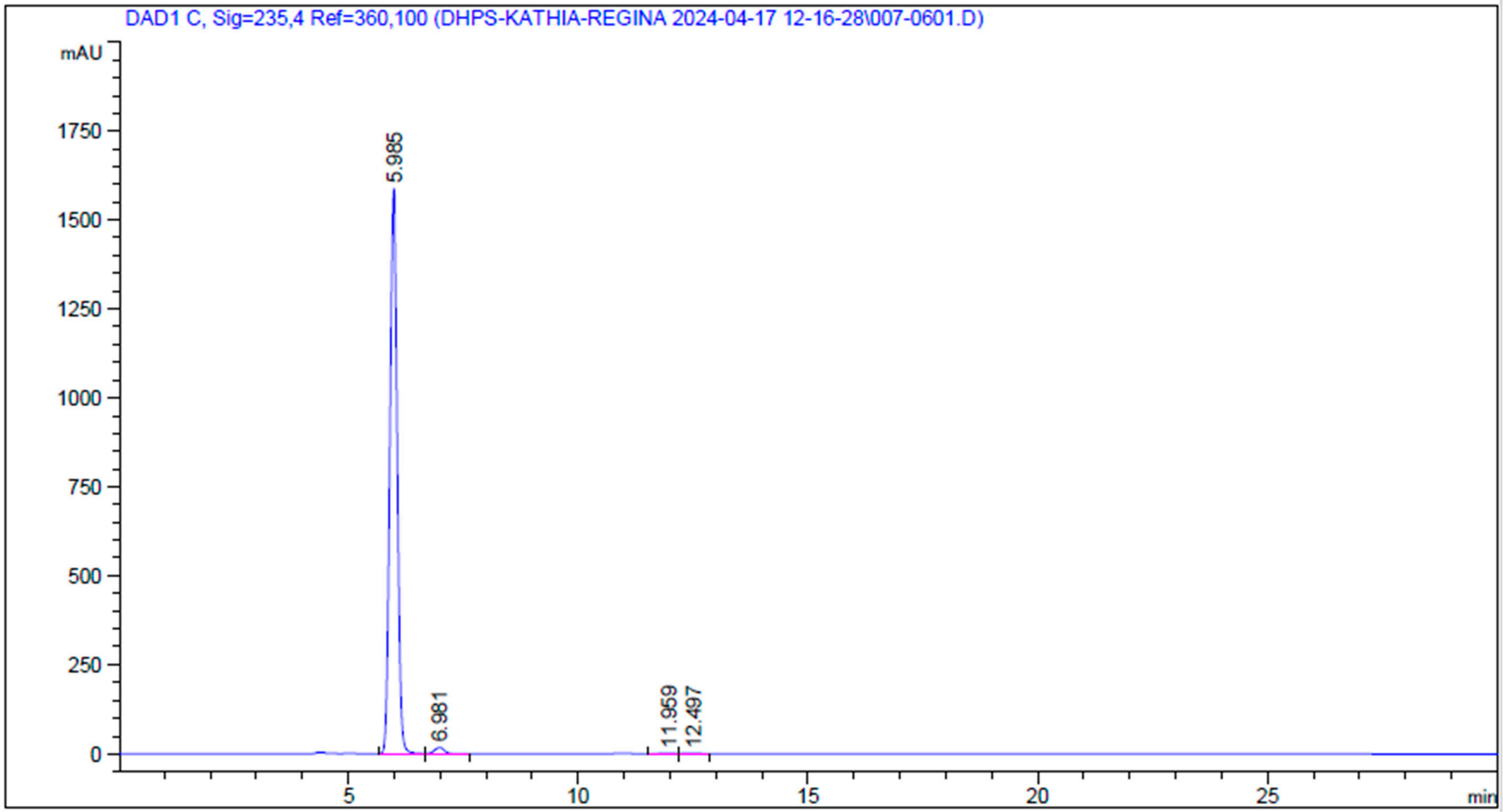

Figure S23. HPLC chromatogram of DHP5 compound (purity: 99.1%).

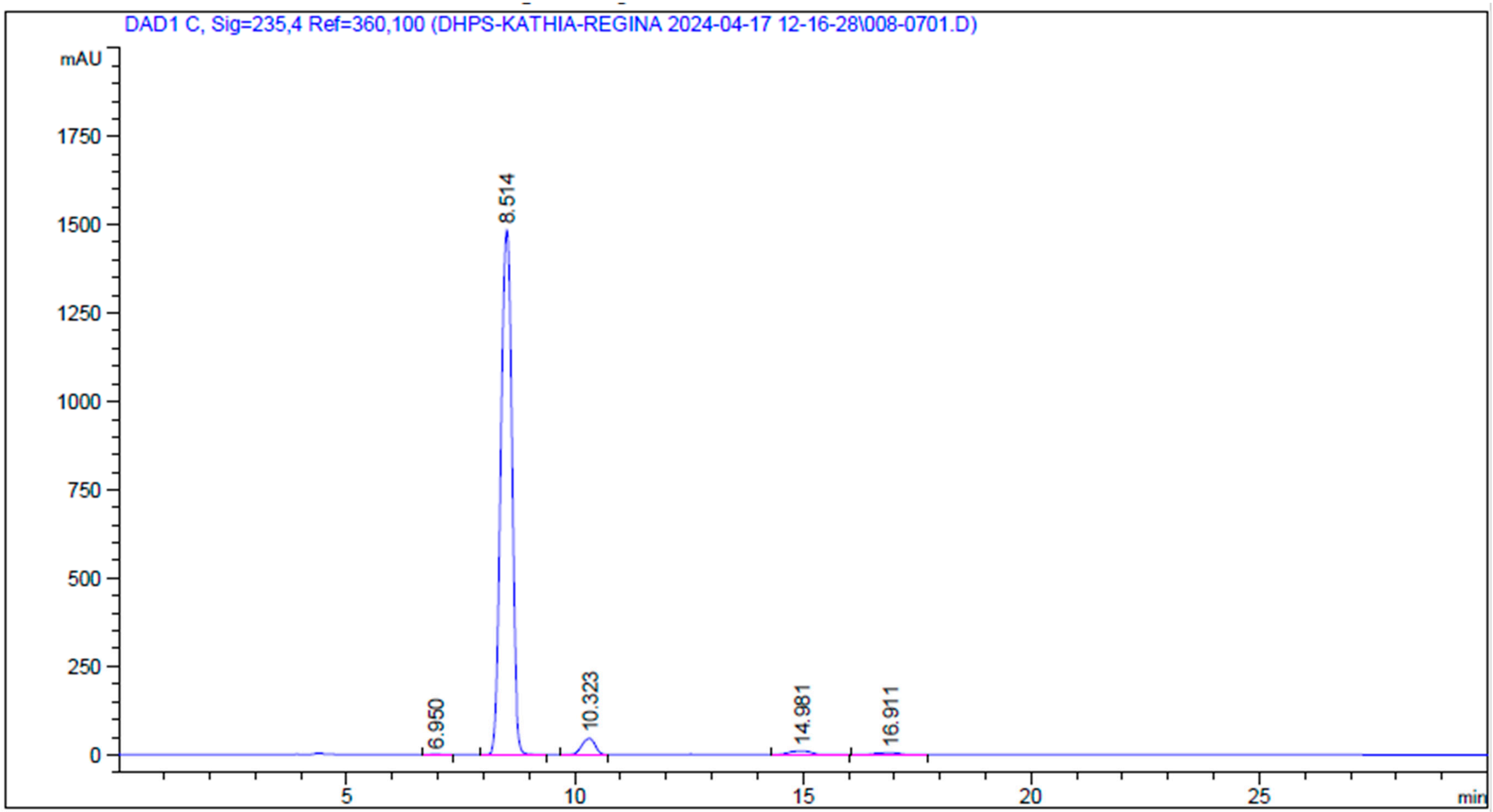

Figure S24. HPLC chromatogram of DHP6 compound (purity: 99.1%).

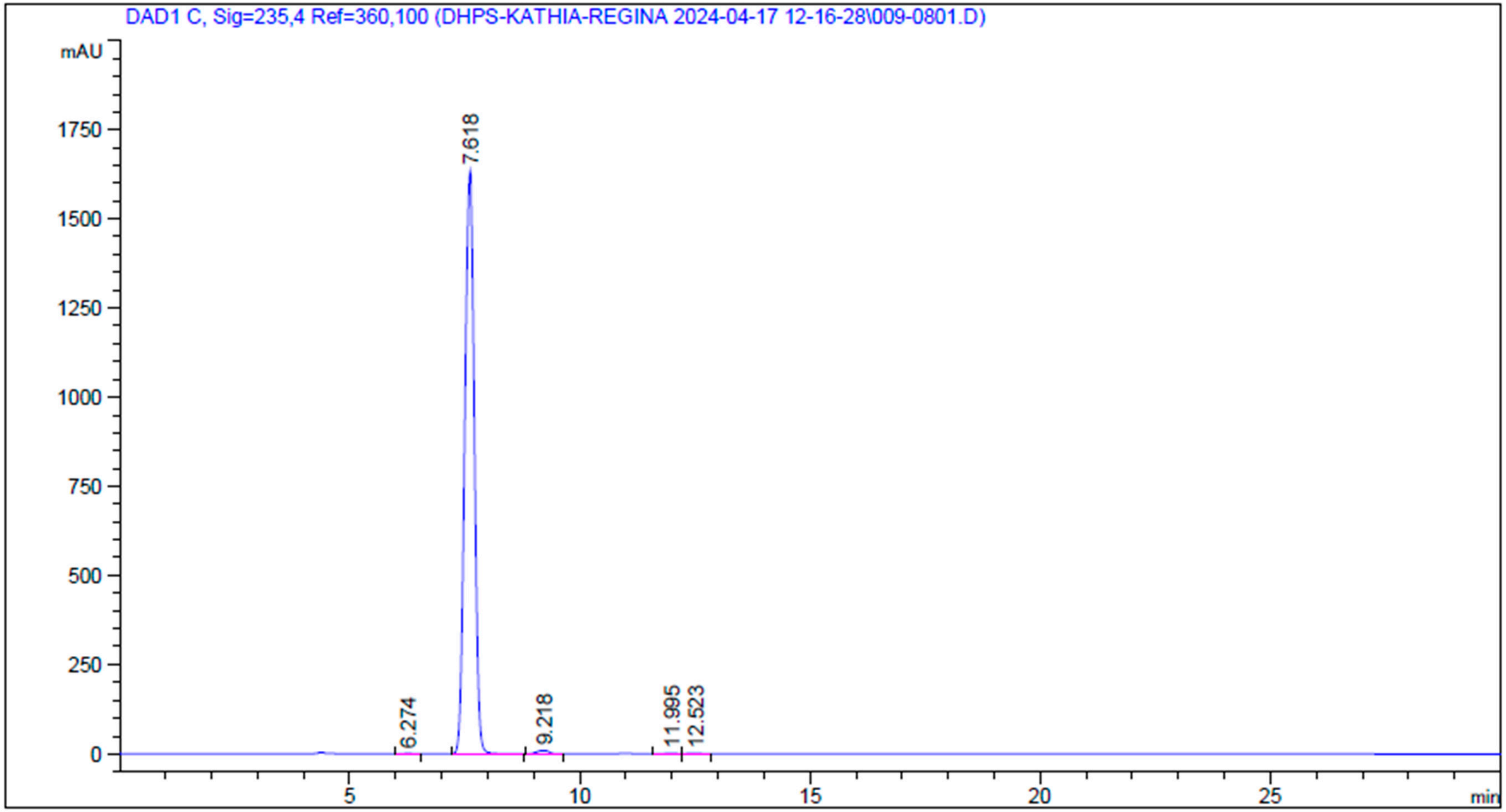

Figure S25. HPLC chromatogram of DHP7 compound (purity: 99.1%).

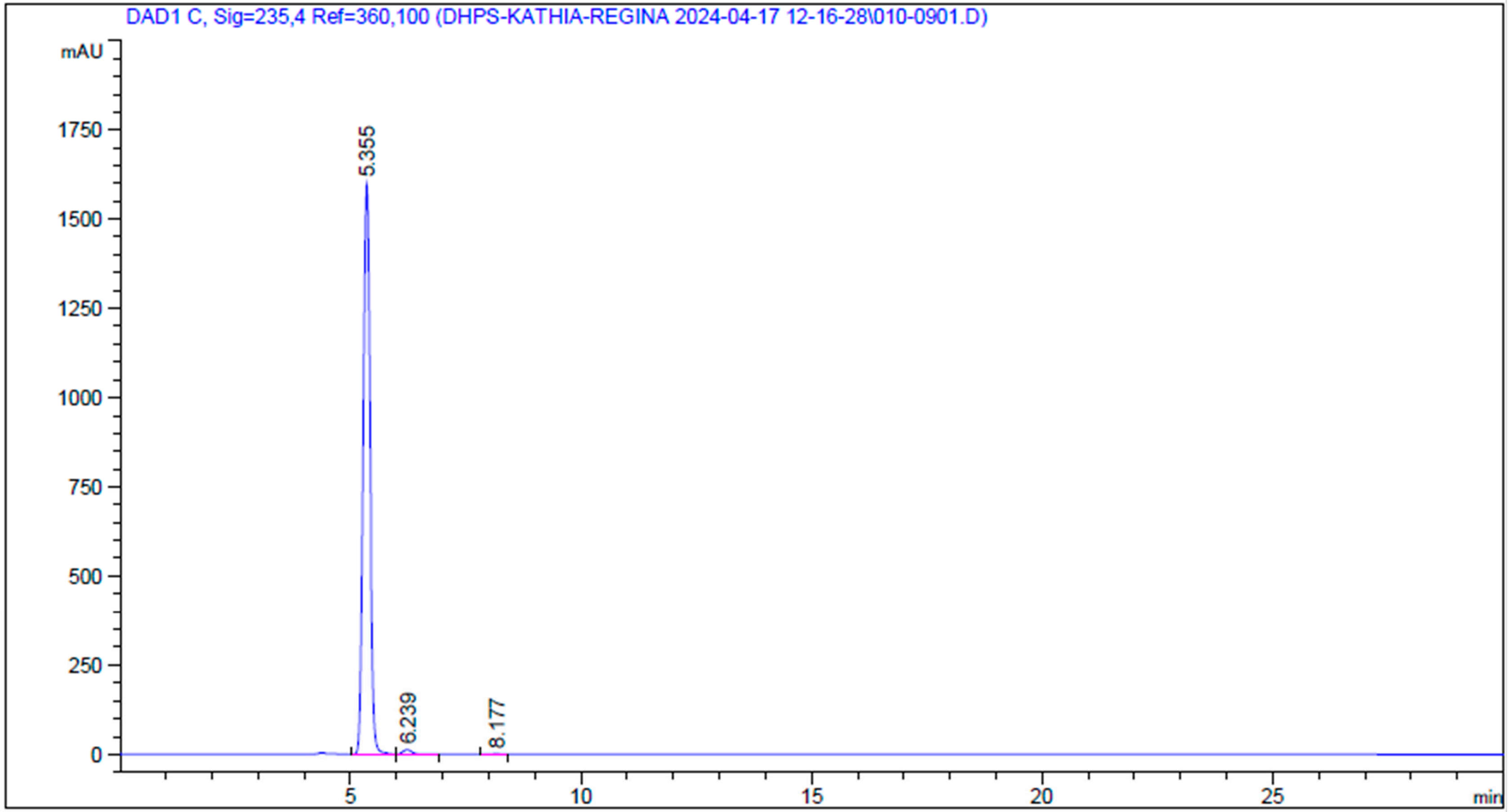

Figure S26. HPLC chromatogram of DHP8 compound (purity: 99.1%).

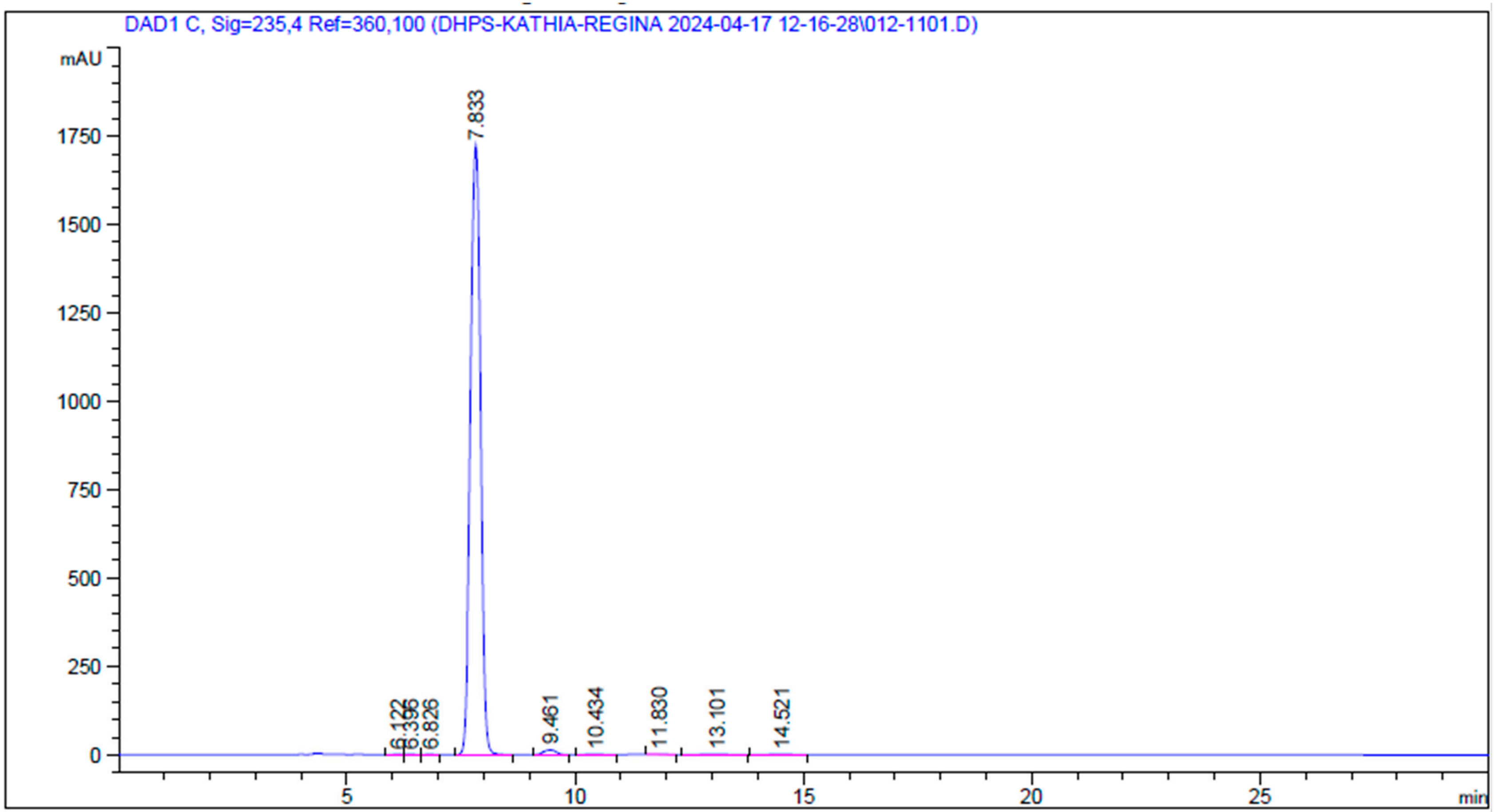

Figure S27. HPLC chromatogram of DHP9 compound (purity: 99.1%).

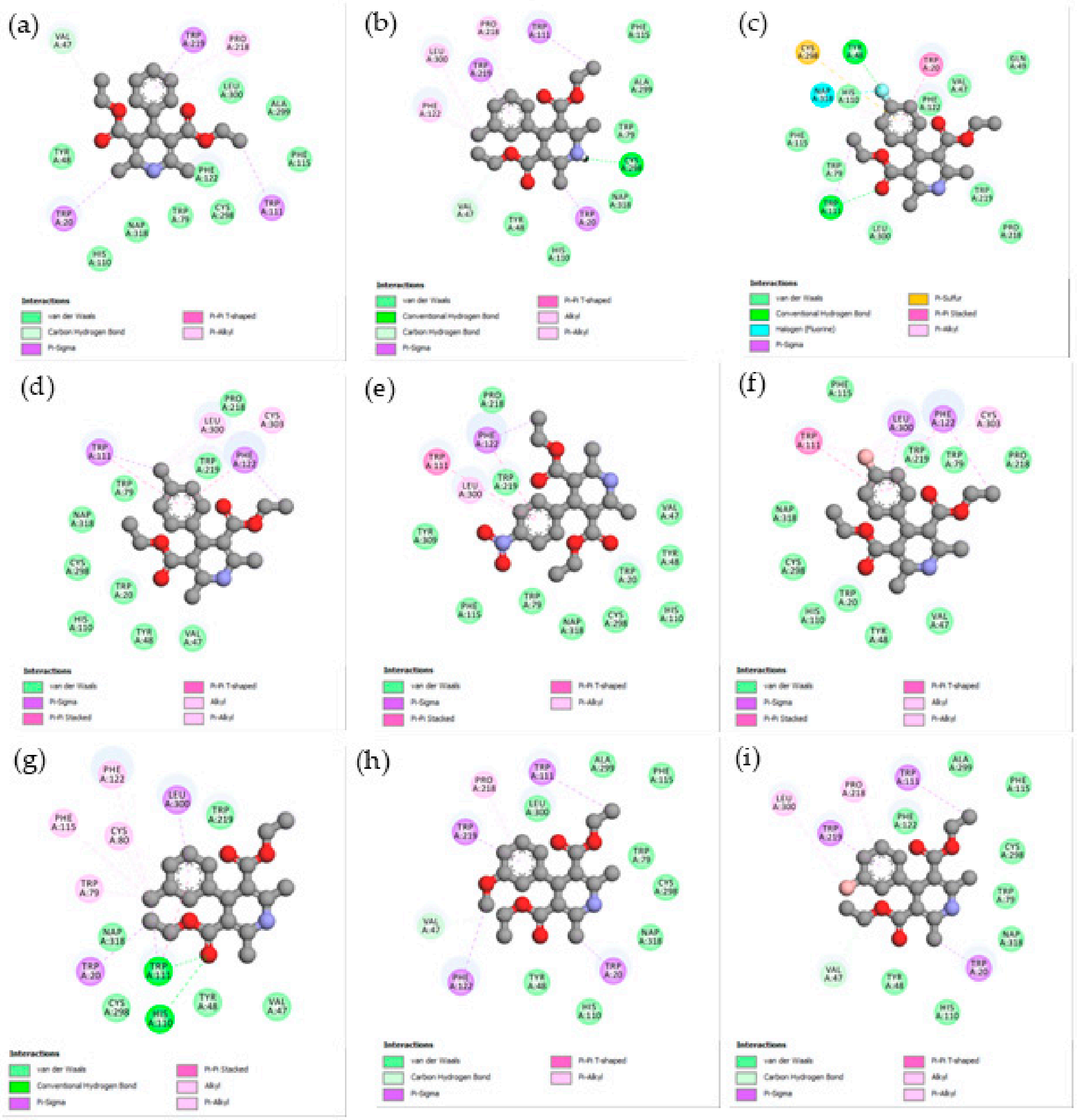

Figure S28. Two-dimensional interaction diagrams of the best-ranked docking poses of DHP1–DHP9 within the binding site of human aldose reductase. Panels correspond to (a) DHP1, (b) DHP2, (c) DHP3, (d) DHP4, (e) DHP5, (f) DHP6, (g) DHP7, (h) DHP8, and (i) DHP9. Docking was performed using the AR–NADP binary complex derived from PDB ID 1PWL. Protein–ligand interactions with surrounding amino acid residues and the NADP cofactor are shown according to the interaction color legend in each panel. Interaction diagrams were generated using Discovery Studio.

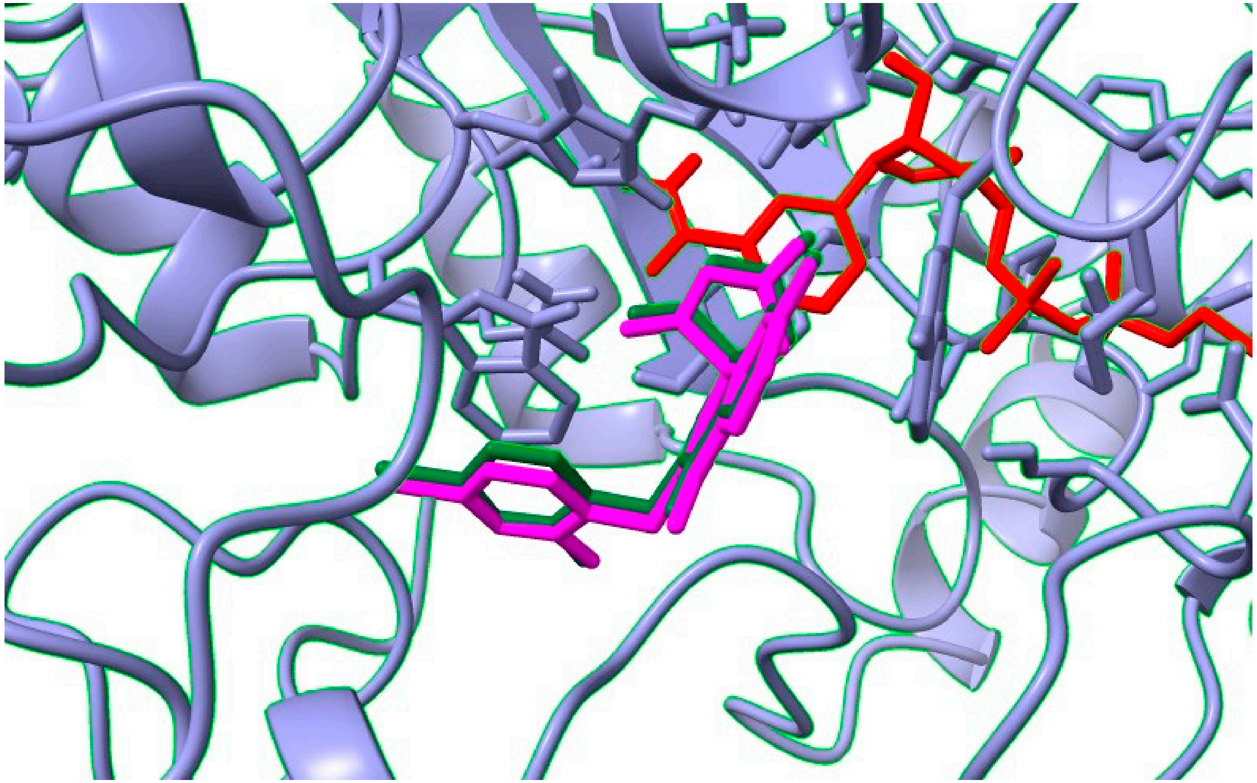

Figure S29. Validation of the molecular docking protocol by redocking of the co-crystallized inhibitor minalrestat. Superposition of the crystallographic pose of minalrestat (green) and the best-ranked redocked pose (magenta) within the active site of human aldose reductase. The NADP cofactor is shown in red, and the protein is represented as a light purple cartoon. The redocked pose closely reproduced the crystallographic binding orientation, with a root-mean-square deviation (RMSD) of 0.41 Å, supporting the reliability of the docking protocol used in this study.

Table S1. Physicochemical Properties of Dihydropyridines										
**Compound**	**Formula**	**Molecular weight (g/mol)**	**Heavy atoms**	**Aromatic heavy atoms**	**Fraction Csp3**	**Rotatable bonds**	**H-bond acceptors**	**H-donors**	**Molar Refractivity**	**TPSA (A)^2**
**DHP1**	$C_{19}H_{23}NO_4$	329.39	24	6	0.37	7	4	1	95.32	64.63
**DHP2**	$C_{20}H_{25}NO_4$	343.29	25	6	0.40	7	4	1	100.28	64.63
**DHP3**	$C_{19}H_{22}FNO_4$	347.38	25	6	0.37	7	5	1	95.27	64.63
**DHP4**	$C_{19}H_{22}ClNO_4$	363.84	25	6	0.37	7	4	1	100.33	64.63
**DHP5**	$C_{19}H_{22}N_2O_6$	374.39	27	6	0.37	8	6	1	104.14	110.45
**DHP6**	$C_{19}H_{22}BrNO_4$	408.29	25	6	0.37	7	4	1	103.02	64.63
**DHP7**	$C_{19}H_{22}ClNO_4$	363.84	25	6	0.37	7	4	1	100.33	64.63
**DHP8**	$C_{20}H_{25}NO_5$	359.42	26	6	0.40	8	5	1	101.81	73.86
**DHP9**	$C_{19}H_{22}BrNO_4$	408.29	25	6	0.37	7	4	1	103.02	64.63

Table S2. Lipophilicity of Dihydropyridines						
**Compound**	**iLOGP**	**XLOGP3**	**WLOGP**	**MLOGP**	**SILICOS-IT**	**Consensus Log P o/w**
**DHP1**	3.34	3.42	2.67	2.22	3.43	3.02
**DHP2**	3.59	3.78	2.98	2.45	3.97	3.35
**DHP3**	3.49	3.52	3.23	2.61	3.87	3.34
**DHP4**	3.59	4.05	3.32	2.72	4.09	3.55
**DHP5**	3.08	3.25	2.58	1.30	1.31	2.30
**DHP6**	3.73	4.11	3.43	2.83	4.12	3.64
**DHP7**	3.65	4.05	3.32	2.72	4.09	3.57
**DHP8**	3.70	3.39	2.68	1.91	3.52	3.04
**DHP9**	3.83	4.11	3.43	2.83	4.12	3.66

Table S3. Water Solubility of Dihydropyridines									
**Compound**	**Log S (ESOL)**	**Solubility (mg/ml)**	**Class**	**Log S (Ali)**	**Solubility (mg/ml)**	**Class**	**Log S (SILICOS-IT)**	**Solubility (mg/ml)**	**Class**
**DHP1**	-3.76	$5.73x10^{-02}$	Soluble	-4.46	$1.15x10^{-02}$	Moderately	-5.26	$1.82x10^{-03}$	Moderately
**DHP2**	-4.07	$2.95x10^{-02}$	Moderately	-4.83	$5.07x10^{-03}$	Moderately	-5.64	$7.9x10\text{-}^{04}$	Moderately
**DHP3**	-3.93	$4.11x10^{-02}$	Soluble	-4.56	$9.54x10^{-03}$	Moderately	-5.53	$1.04x10\text{-}^{03}$	Moderately
**DHP4**	-4.36	$1.58x10^{-02}$	Moderately	-5.11	$2.82x10^{-03}$	Moderately	-5.85	$5.13x10\text{-}^{04}$	Moderately
**DHP5**	-3.85	$5.35x10^{-02}$	Soluble	-5.24	$2.14x10^{-03}$	Moderately	-4.61	$9.25x10\text{-}^{03}$	Moderately
**DHP6**	-4.68	$8.60x10^{-03}$	Moderately	-5.17	$2.74x10^{-03}$	Moderately	-6.05	$3.63x10\text{-}^{04}$	Poorly
**DHP7**	-4.36	$1.58x10^{-02}$	Moderately	-5.11	$2.82x10^{-03}$	Moderately	-5.85	$5.13x10\text{-}^{04}$	Moderately
**DHP8**	-3.85	$5.11x10^{-02}$	Soluble	-4.62	$8.62x10^{-03}$	Moderately	-5.37	$1.55x10^{-03}$	Moderately
**DHP9**	-4.68	$8.60x10^{-03}$	Moderately	-5.17	$2.74x10^{-03}$	Moderately	-6.05	$3.63x10^{-04}$	Poorly

Table S4. Pharmacokinetics of Dihydropyridines									
**Compound**	**GI absorption**	**BBB permeant**	**P-gp substrate**	**CYP1A2 inhibitor**	**CYP2C19 inhibitor**	**CYP2C9 inhibitor**	**CYP2D6 inhibitor**	**CYP3A4 inhibitor**	**Log Kp (skin permeation) (cm/s)**
**DHP1**	High	Yes	No	Yes	Yes	Yes	No	Yes	-5.88
**DHP2**	High	Yes	No	Yes	Yes	Yes	NO	Yes	-5.71
**DHP3**	High	Yes	No	Yes	Yes	Yes	No	Yes	-5.92
**DHP4**	High	Yes	No	Yes	Yes	Yes	No	Yes	-5.64
**DHP5**	High	No	No	Yes	Yes	Yes	Yes	Yes	-6.28
**DHP6**	High	Yes	No	Yes	Yes	Yes	No	Yes	-5.87
**DHP7**	High	Yes	No	Yes	Yes	Yes	No	Yes	-5.64
**DHP8**	High	Yes	No	Yes	Yes	Yes	Yes	Yes	-6.09
**DHP9**	High	Yes	No	Yes	Yes	Yes	No	Yes	-5.87

Table S5. Druglikeness and Medicinal Chemistry of Dihydropyridines								
**Druglikeness**							**Medicinal Chemistry**	
**Compound**	**Lipinski**	**Ghose**	**Veber**	**Egan**	**Muegge**	**Bioavailability score**	**PAINS**	**Synthetic accessibility**
**DHP1**	Yes; 0 violations	Yes	Yes	Yes	Yes	0.55	0 alert	3.98
**DHP2**	Yes; 0 violations	Yes	Yes	Yes	Yes	0.55	0 alert	4.09
**DHP3**	Yes; 0 violations	Yes	Yes	Yes	Yes	0.55	0 alert	3.97
**DHP4**	Yes; 0 violations	Yes	Yes	Yes	Yes	0.55	0 alert	3.95
**DHP5**	Yes; 0 violations	Yes	Yes	Yes	Yes	0.55	0 alert	4.09
**DHP6**	Yes; 0 violations	Yes	Yes	Yes	Yes	0.55	0 alert	3.99
**DHP7**	Yes; 0 violations	Yes	Yes	Yes	Yes	0.55	0 alert	3.95
**DHP8**	Yes; 0 violations	Yes	Yes	Yes	Yes	0.55	0 alert	4.10
**DHP9**	Yes; 0 violations	Yes	Yes	Yes	Yes	0.55	0 alert	4.06

Materials and Methods.

Nuclear Magnetic Resonance Spectroscopy (NMR) Characterization of DHP1–DHP9.

DHP1: [Diethyl 2,6-dimethyl-4-phenyl-1,4-dihydropyridine-3,5-dicarboxylate]. ^{1}H NMR (300 MHz, $CDCl_3$) δ 7.33–7.06 (m, 5H, Ar–H), 5.67 (s, 1H, NH), 4.99 (s, 1H, H-4), 4.19–3.98 (m, 4H, 2 × OCH_2), 2.33 (s, 6H, 2 × CH_3), 1.22 (t, J = 7.1 Hz, 6H, 2 × CH_3). ^{13}C NMR (75 MHz, $CDCl_3$) δ 167.77, 147.88, 143.97, 128.14, 127.96, 126.23, 104.31, 59.86, 39.75, 19.73, 14.38.

DHP2: [Diethyl 2,6-dimethyl-4-(3-methylphenyl)-1,4-dihydropyridine-3,5-dicarboxylate]. ^{1}H NMR (300 MHz, $CDCl_3$) δ 7.14–6.90 (m, 4H, Ar–H), 5.59 (s, 1H, NH), 4.95 (s, 1H, H-4), 4.20–3.98 (m, 4H, 2 × OCH_2), 2.33 (s, 6H, 2 × CH_3), 2.28 (s, 3H, Ar–CH_3), 1.23 (t, J = 7.1 Hz, 6H, 2 × CH_3). ^{13}C NMR (75 MHz, $CDCl_3$) δ 167.79, 147.77, 143.86, 137.26, 128.96, 127.83, 127.03, 125.22, 104.36, 59.84, 39.66, 21.70, 19.80, 14.38.

DHP3: [Diethyl 2,6-dimethyl-4-(4-fluorophenyl)-1,4-dihydropyridine-3,5-dicarboxylate] . ^{1}H NMR (300 MHz, $CDCl_3$) δ 7.30–7.17 (m, 2H, Ar–H), 6.95–6.81 (m, 2H, Ar–H), 5.61 (s, 1H, NH), 4.96 (s, 1H, H-4), 4.20–3.98 (m, 4H, 2 × OCH_2), 2.33 (s, 6H, 2 × CH_3), 1.22 (t, J = 7.1 Hz, 6H, 2 × CH_3). ^{13}C NMR (75 MHz, $CDCl_3$) δ 167.65, 159.87, 143.89, 129.65, 129.54, 114.77, 114.50, 104.34, 59.92, 39.18, 19.77, 14.39.

DHP4: [Diethyl 2,6-dimethyl-4-(4-chlorophenyl)-1,4-dihydropyridine-3,5-dicarboxylate]. ^{1}H NMR (300 MHz, $CDCl_3$) δ 7.25–7.14 (m, 4H, Ar–H), 5.63 (s, 1H, NH), 4.95 (s, 1H, H-4), 4.19–3.98 (m, 4H, 2 × OCH_2), 2.33 (s, 6H, 2 × CH_3), 1.22 (t, J = 7.1 Hz, 6H, 2 × CH_3). ^{13}C NMR (75 MHz, $CDCl_3$) δ 167.55, 146.45, 144.07, 131.83, 129.57, 128.08, 104.06, 59.97, 39.39, 19.79, 14.40.

DHP5: [Diethyl 2,6-dimethyl-4-(3-nitrophenyl)-1,4-dihydropyridine-3,5-dicarboxylate]. ^{1}H NMR (300 MHz, $CDCl_3$) δ 8.21–7.32 (m, 4H, Ar–H), 5.74 (s, 1H, NH), 5.09 (s, 1H, H-4), 4.20–3.98 (m, 4H, 2 × OCH_2), 2.37 (s, 6H, 2 × CH_3), 1.22 (t, J = 7.1 Hz, 6H, 2 × CH_3). ^{13}C NMR (75 MHz, $CDCl_3$) δ 167.22, 150.02, 148.28, 144.76, 134.66, 128.71, 123.27, 121.47, 103.53, 60.13, 40.10, 19.82, 14.37.

DHP6: [Diethyl 2,6-dimethyl-4-(4-bromophenyl)-1,4-dihydropyridine-3,5-dicarboxylate]. ^{1}H NMR (300 MHz, $CDCl_3$) δ 7.37–7.25 (m, 2H, Ar–H), 7.22–7.10 (m, 2H, Ar–H), 5.61 (s, 1H, NH), 4.94 (s, 1H, H-4), 4.19–3.98 (m, 4H, 2 × OCH_2), 2.33 (s, 6H, 2 × CH_3), 1.22 (t, J = 7.1 Hz, 6H, 2 × CH_3). ^{13}C NMR (75 MHz, $CDCl_3$) δ 167.52, 146.95, 144.08, 131.03, 129.99, 120.02, 103.99, 59.97, 39.47, 19.79, 14.40.

DHP7: [Diethyl 2,6-dimethyl-4-(3-chlorolphenyl)-1,4-dihydropyridine-3,5-dicarboxylate]. ^{1}H NMR (300 MHz, $CDCl_3$) δ 7.28–6.96 (m, 4H, Ar–H), 5.63 (s, 1H, NH), 4.96 (s, 1H, H-4), 4.21–3.98 (m, 4H, 2 × OCH_2), 2.34 (s, 6H, 2 × CH_3), 1.23 (t, J = 7.1 Hz, 6H, 2 × CH_3). ^{13}C NMR (75 MHz, $CDCl_3$) δ 167.48, 149.87, 144.25, 133.70, 129.18, 128.42, 126.48, 126.40, 103.86, 59.98, 39.83, 19.80, 14.38.

DHP8: [Diethyl 2,6-dimethyl-4-(3-methoxyphenyl)-1,4-dihydropyridine-3,5-dicarboxylate]. ^{1}H NMR (300 MHz, $CDCl_3$) δ 7.18–6.63 (m, 4H, Ar–H), 5.62 (s, 1H, NH), 4.98 (s, 1H, H-4), 4.20–3.99 (m, 4H, 2 × OCH_2), 3.76 (s, 3H, Ar–OCH_3), 2.33 (s, 6H, 2 × CH_3), 1.23 (t, J = 7.1 Hz, 6H, 2 × CH_3). ^{13}C NMR (75 MHz, $CDCl_3$) δ 167.72, 159.38, 149.46, 144.02, 128.81, 120.71, 114.36, 111.01, 104.17, 59.88, 55.20, 39.67, 19.78, 14.43.

DHP9: [Diethyl 2,6-dimethyl-4-(3-bromophenyl)-1,4-dihydropyridine-3,5-dicarboxylate]. ^{1}H NMR (300 MHz, $CDCl_3$) δ 7.43–7.02 (m, 4H, Ar–H), 5.62 (s, 1H, NH), 4.95 (s, 1H, H-4), 4.21–3.98 (m, 4H, 2 × OCH_2), 2.34 (s, 6H, 2 × CH_3), 1.23 (t, J = 7.1 Hz, 6H, 2 × CH_3). ^{13}C NMR (75 MHz, $CDCl_3$) δ 167.46, 150.17, 144.26, 131.37, 129.55, 129.31, 126.95, 122.07, 103.86, 59.99, 39.86, 19.80, 14.38.

Raw and Processed NMR Data.

The original raw ^{1}H and ^{13}C NMR acquisition data for compounds DHP1–DHP9 are provided separately in the compressed archive **Raw_NMR_Data_DHP1-DHP9.zip**. In addition, the corresponding processed and editable NMR spectra are provided in MestReNova format as **Processed_NMR_Spectra_DHP1-DHP9.mnova**. The processed spectrum is also presented in the corresponding figures of this Supplementary Information file.

***In silico* Prediction of Physicochemical, Pharmacokinetic, and Drug-Likeness Properties.**

The physicochemical, lipophilicity, water-solubility, pharmacokinetic, drug-likeness, and medicinal chemistry properties of the synthesized 1,4-dihydropyridine derivatives were predicted using the SwissADME web platform (Swiss Institute of Bioinformatics, Lausanne, Switzerland; http://www.swissadme.ch/; accessed in March 2025). The molecular structures of the compounds were submitted individually as SMILES strings, and the corresponding predictions were obtained using the models implemented in SwissADME. Physicochemical descriptors included molecular weight, number of heavy atoms and aromatic heavy atoms, fraction of sp^3-hybridized carbons (fraction Csp^3), number of rotatable bonds, hydrogen-bond acceptors and donors, molar refractivity, and topological polar surface area (TPSA). Lipophilicity was evaluated using the iLOGP, XLOGP3, WLOGP, MLOGP, and SILICOS-IT models, together with the consensus Log P value, whereas water solubility was estimated using the ESOL, Ali, and SILICOS-IT models. Pharmacokinetic predictions included gastrointestinal absorption, blood–brain barrier permeation, P-glycoprotein substrate prediction, inhibition of CYP1A2, CYP2C19, CYP2C9, CYP2D6, and CYP3A4, and skin permeation (log Kp). Drug-likeness was assessed according to the Lipinski, Ghose, Veber, Egan, and Muegge filters. Medicinal chemistry parameters included PAINS and Brenk alerts, lead-likeness, and synthetic accessibility. The complete predicted parameters are presented in Tables S1–S5.
